# Ancient Human Genomes and Environmental DNA from the Cement Attaching 2,000-Year-Old Head Lice Nits

**DOI:** 10.1093/molbev/msab351

**Published:** 2021-12-28

**Authors:** Mikkel W Pedersen, Catia Antunes, Binia De Cahsan, J Víctor Moreno-Mayar, Martin Sikora, Lasse Vinner, Darren Mann, Pavel B Klimov, Stuart Black, Catalina Teresa Michieli, Henk R Braig, M Alejandra Perotti

**Affiliations:** 1 GLOBE Institute, Faculty of Health and Medical Science, University of Copenhagen, Copenhagen, Denmark; 2 Ecology and Evolutionary Biology Section, School of Biological Sciences, University of Reading, Reading, United Kingdom; 3 Oxford University Museum of Natural History, Oxford, United Kingdom; 4 School of Natural Sciences, Bangor University, Bangor, Wales, United Kingdom; 5 Department of Ecology and Evolutionary Biology, University of Michigan, Museum of Zoology, Ann Arbor, MI, USA; 6 Department of Geography and Environmental Science, Wager Building, University of Reading, Reading, United Kingdom; 7 Instituto de Investigaciones Arqueológicas y Museo “Prof. Mariano Gambier”, Universidad Nacional de San Juan, San Juan, Argentina; 8 Institute and Museum of Natural Sciences, Faculty of Exact, Physical and Natural Sciences, National University of San Juan, San Juan, Argentina

**Keywords:** ancient host genomes, ancient head lice, Merkel cell polyomavirus, aDNA

## Abstract

Over the past few decades, there has been a growing demand for genome analysis of ancient human remains. Destructive sampling is increasingly difficult to obtain for ethical reasons, and standard methods of breaking the skull to access the petrous bone or sampling remaining teeth are often forbidden for curatorial reasons. However, most ancient humans carried head lice and their eggs abound in historical hair specimens. Here we show that host DNA is protected by the cement that glues head lice nits to the hair of ancient Argentinian mummies, 1,500–2,000 years old. The genetic affinities deciphered from genome-wide analyses of this DNA inform that this population migrated from north-west Amazonia to the Andes of central-west Argentina; a result confirmed using the mitochondria of the host lice. The cement preserves ancient environmental DNA of the skin, including the earliest recorded case of Merkel cell polyomavirus. We found that the percentage of human DNA obtained from nit cement equals human DNA obtained from the tooth, yield 2-fold compared with a petrous bone, and 4-fold to a bloodmeal of adult lice a millennium younger. In metric studies of sheaths, the length of the cement negatively correlates with the age of the specimens, whereas hair linear distance between nit and scalp informs about the environmental conditions at the time before death. Ectoparasitic lice sheaths can offer an alternative, nondestructive source of high-quality ancient DNA from a variety of host taxa where bones and teeth are not available and reveal complementary details of their history.

## Introduction

Over the last 30 years, ancient DNA (aDNA) has proven to be a key source of information about past organism assemblages, diversity, and evolution. To date, aDNA has been obtained from a variety of materials including bones and teeth (Poinar et al. 2006; Shapiro and Hofreiter 2012; Feldman et al. 2016; Massilani et al. 2020; Curry et al. 2021; Woods et al. 2021), bird toe pads (Shapiro and Hofreiter 2012), coprolites (Hofreiter et al. 2000), keratin and chitin (Shapiro and Hofreiter 2012; Gazda et al. 2020), eggshells (Shapiro and Hofreiter 2012), chewed birch pitch (Jensen et al. 2019), and sediments (Shapiro and Hofreiter 2012; Slon et al. 2017; Rohland et al. 2018; Zhang et al. 2020) from archaeological sites, geological settings, and museum collections. The best-preserved DNA from ancient humans and animals is recovered from petrous bones and teeth due to their dense structure (Hansen et al. 2017; Sirak et al. 2017; Pilli et al. 2018; Pinhasi et al. 2019). However, extracting DNA from these materials is destructive and often results in irreparable damage to unique archaeological specimens and in addition, depletes the sample for future physical, biological, and taphonomic analyses (Geigl and Grange 2018; Ponce de Leon et al. 2018; Prendergast and Sawchuk 2018; Palsdottir et al. 2019; Sirak et al. 2020).

As the demand for human bones and teeth for destructive analyses have increased over the past 20 years, so have cases of unreported, indiscriminate exploitation of historical and unique specimens (Makarewicz et al. 2017; Ponce de Leon et al. 2018). Concerns have also been raised over the lack of adequate documentation for the procedures resulting in damaged and depleted material or specimens. Only very recently museums in South America have produced guidelines and best practice protocols, adhering to ethical as well as local, regional, national, and international legislation, which require applications to the Ministry of Culture and Antiquities in Latin American countries (O’Rourke et al. 2000; Claw et al. 2017; Geigl and Grange 2018; Fox and Hawks 2019; Palsdottir et al. 2019; Sawchuk and Prendergast 2019; Wagner et al. 2020). Therefore, there is a demand for alternative, nondestructive procedures to the current methods and to explore other material sources such as dental calculus, a source of host and/or microbial and dietary DNA (Warinner et al. 2014; Ozga et al. 2016; Nieves-Colón et al. 2020). Another underexploited, noninvasive source are parasites, as they often contain and preserve ancient host DNA. Additionally, they hold the potential to not only inform about past dispersal and host interactions but unravel host health conditions and even the cause of death of individuals (Panagiotakopulu et al. 2007, 2013; Dittmar 2009; Dittmar 2014; Koungoulos and Contos 2019).

To date, parasites found in 19th century collections of natural history museums as well as archaeological sites have mainly been studied morphologically. Only a few, more recent studies have gathered short fragments of DNA from ancient parasites (Dittmar et al. 2003; Raoult et al. 2008; Dittmar 2009; Wood 2018). Dittmar et al. (2003) used a single nuclear DNA marker extracted from *Pulex* fleas from animal mummies (dogs and guinea pigs) belonging to the Chiribaya culture, South Peru (dated 1,120 years BP [Before Present]). In addition, Raoult et al. (2008) successfully retrieved mitochondrial DNA fragments of ancient human head lice also from the Chiribaya culture (dated 978 years BP).

**Fig. 1.**
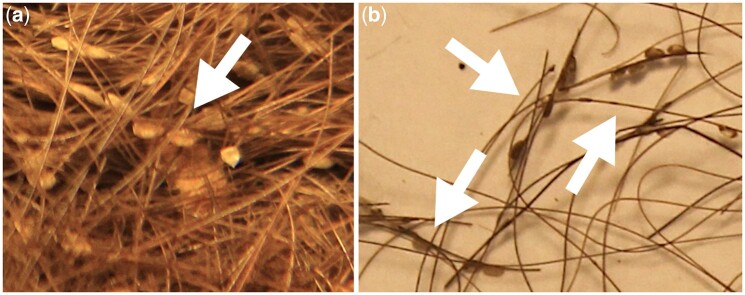
Ancient, crowded infestations. (*a*) From sample Chi-8-Nit (∼300 BP), a textile made of human hair; (*b*) hair from the mummy sample SJArg-4-Nit. Both panels show chains of Nits along the same hair shaft, equating to nit clusters, which are markers of severe infestations.

The louse, *Pediculus humanus*, is the most ubiquitous human ectoparasite. It is an obligatory bloodsucking parasite (Ferris 1951) that has accompanied hominins since the split from our primate relatives (Kittler et al. 2003; Reed et al. 2004, 2007; Durden et al. 2020). These human lice can be found in the form of two ecotypes or subspecies, one nesting in the head hair, *Pediculus humanus capitis*, and the other in the clothing fabric, *P. humanus humanus*. Lice can produce supernumerary infestations (Alexander 1984; Lambiase and Perotti 2019), characterized by the occurrence of a large number of nits (lice eggs) homogeneously distributed on head hairs and, especially concentrated on the hair shaft toward the root end (Lambiase and Perotti 2019). Crowded infestations have frequently been recorded from ancient human mummies, with great numbers of nits attached not only to hair (see fig. 1) but to their clothing as well (Araujo et al. 2000; Reinhard and Buikstra 2003; Arriaza et al. 2012; Dutra et al. 2014; Lynnerup 2015).

Lice are highly prevalent not just on human but on most mammal hairs, especially on late Holocene samples of hair or animal skins. A good reservoir of ancient lice still exists in historical museum collections, with a number of new species of ectoparasites being described from preserved hair, skins, furs, and feathers kept in these collections (Ewing 1938; Eichler 1971; Eberhard 2002; de la Cruz et al. 2003; Huchet et al. 2013; Sakalauskaitė-Juodeikienė et al. 2018). Pioneered by Ewing (Ewing 1924, 1926, 1938), morphological examination and identification of human head lice found on ancient mummies from America and Egypt showed that lice passively dispersed around the world alongside humans, shedding light on human evolution, past migrations, and dispersal. Ice or permafrost mummies carry diverse and abundant ectoparasitic faunas too (Panagiotakopulu et al. 2007; Tridico et al. 2014), with Greenland human and animal mummies and remains showing high prevalence of severe infestations, to such an extent that adult lice have even been found inside the gut of mummies (Lynnerup 2015). Other early records from archaeological sites come not just from the Americas, but also from human remains near the Dead Sea, dating to 9,000 years BP (Zias and Mumcuoglu 1991), the Loulan mummies of China (3,800 years BP) (Wen et al. 1987) or the hair glued to the human remains found in Herculaneum, Italy (∼2,000 years BP) (Capasso and Di Tota 1998).

Louse nits and the empty sheaths can be as informative as adult lice about their host’s history (Ewing 1938; Reed et al. 2004, 2007; Arriaza et al. 2008). Nits are individually attached to the hair shaft at approx. 5 mm from the root, with a cement or glue that forms the nit sheath (Lambiase and Perotti 2019). This cement is a rich proteinaceous substance, with high content of saturated fatty acids and aliphatic components, secreted by the accessory glands of the reproductive organs of the female (Burkhart et al. 1999; Burkhart and Burkhart 2005; Park et al. 2019). The hardiness of the compounds not only secures the nits to hair for up to 10,000 years but also manages to preserve detached sheaths beyond the hair’s life (Linardi et al. 1988; Araujo et al. 2000; Arriaza et al. 2014). Considering the preservation capabilities of this strong natural substance, we hypothesized that nit cement is able to trap and preserve host DNA.

In this work, we investigated the potential for ancient nit sheaths (*Pediculus* Linnaeus) to yield human DNA from host cells trapped inside their cement. This allowed us to explore the suitability of this DNA for genome-wide analysis, to compare with current sources of ancient human DNA (e.g., teeth, bones), and to study aspects of preservation, genome coverage, haplotyping, sexing of individuals, and phylogeographic history. Eight ancient South American human remains were sampled for hair containing nits. The hair of two of these specimens, SJArg-1-Nit and SJArg-2-Nit, showed the presence of a few nits; a maximum of six from each individual were subjected to DNA extraction. For comparison of DNA preservation and coverage, we obtained one tooth (SJArg-1-Tooth) from the same sedimentary layer as SJArg-1-Nit, and a petrous bone (SJArg-3-Petrous) from an individual (SJArg-3) from the same culture as SJArg-1 and SJArg-2 but located at an upper layer. For comparison of host DNA from nits with host DNA from lice bloodmeal (inside their gut), two adult lice from the Denny’s historical Anoplura collection from the Oxford University Museum of Natural History were included. They were recovered ∼170 years ago (between 1849 and 1859) from a Dayak/Dyak/Dayuh native of Borneo (sample Dyak-Louse). Because lice are species specific parasites that parallel the history of their hosts, *Pediculus* genetic affinities were also investigated using mitochondrial genes. In addition to the genetic studies on nits, three of the nit samples were studied using cytogenetic methods to detect and validate DNA trapped in cement, and we compared these findings to the genomic data generated. Lastly, we investigated the level of infestation to gather information on the health status, carried out morphometric characterizations such as length of cement coverage to assess preservation, and nit/sheath distance to scalp to unravel environmental conditions such as variations in ambient temperature just before the death of these ancient individuals.

## Results

### Radiocarbon Dating

A total of ten hand-picked and cleaned hair fragments from SJArg-1-Nit were radiocarbon dated at the Scottish Universities Environmental Research Centre (SUERC) Radiocarbon laboratory (SUERC-81423) to 1,461 cal. years BP (median value; 1,389–1,520 cal. years BP; 95.4% probability). This is near to the end of Early Period in Argentina (500 BCE–650 CE) and is equivalent to the early intermediate period in the Central Andes (0–750 CE), which has one of the best constructed archaeological chronologies. It is also equivalent to Nazca and Tiwanaku cultures in Peru and Bolivia, respectively. This radiocarbon date is in good agreement with the previous age estimates (∼1,460 cal. years BP) based on radiocarbon dating of associated bulk sediments. We therefore did not re-date the other three specimens used for DNA analysis, SJArg-1-Tooth, SJArg-2-Nit, and SJArg-3-Petrous, for which radiocarbon ages had also been obtained by bulk sediments and which were found to be 1,461 (1,345–1,586), 1,934 (1,689–2,314), and 1,018 (926–1,175) cal. years BP with 95.4% probability. Radiocarbon ages of all eight mummies together with other age estimates based on cultural chronologies can be found in supplementary table S1, Supplementary Material online.

### Sheath Cement Length and Nit Distance to Scalp

Sheaths from all eight specimens, six Argentinean: SJArg-1, 2, 4, 5, 6, 7-Nit and two Chilean: Chi-8, 9-Nit, were analyzed for cement coverage (details of the eight specimens are found in supplementary table S2, Supplementary Material online). This important parameter relates to the preservation status of the mummies, even if they are of the same age, they may have been exposed to different environmental and storage conditions, and therefore, potentially could relate to DNA damage and preservation. Cement length was homogeneous within ages and within seven out of the eight mummies; with the youngest remains, Chi-8-Nit showing most variation between cement lengths. We found across all samples an average cement length of 570.45 µm, with the smallest length of 385 µm in SJArg-2-Nit (1,934 cal. years BP), with the maximum length of 841.0 µm found in Chi-8-Nit (∼300 years old). Therefore, the older the remains the shorter the fragment of cement protecting the hair shaft (*r* = −0.73). The variations between samples of different ages were found significant (Tukey’s pairwise mean comparisons, *P* < 0.01), with the oldest samples from SJArg-2-Nit differing from most of the ages (supplementary tables S3 and S4, Supplementary Material online).

To obtain information of the environmental conditions at the time before death, we measured the shaft distance from nit (with embryo) to root in three mummies (SJArg-1-Nit, SJArg-2-Nit, and SJArg-4-Nit), as the colder the air temperature the closer to the scalp the louse lays its eggs (see Supplementary Material online). We found the distance between the nits and hair root for each of the three mummies to average 4.25 (±2.5), 4.7 (±2.1), and 6.8 (±1.8) mm, for SJArg-4-Nit, SJArg-1-Nit, and SJArg-2-Nit, respectively. Analysis of distance from root within the hosts indicated that for SJArg-1-Nit and SJArg-2-Nit, distances were homogeneous (Shapiro–Wilk *W* = 0.95 and 0.86, and *P* = 0.70 and 0.096, respectively), whereas this distance in SJArg-4-Nit varied significantly (Shapiro–Wilk *W* = 0.78, *P* < 0.001). Nits carrying embryos are eggs laid shortly before the host’s death and were found at a shorter distance than the generally expected ∼7 mm from the root (∼5 mm from scalp), occurring at high frequency at an unusually shorter distance of 2–4 mm (supplementary tables S5 and S6, Supplementary Material online).

### Host Cell Entrapment by Nit Cement

**Fig. 2.**
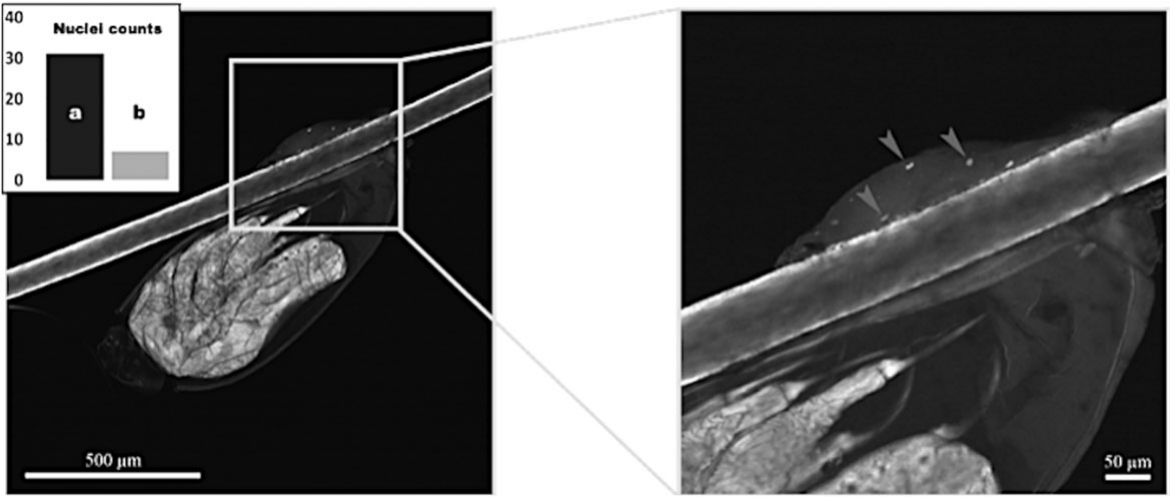
Fluorescence confocal scanning of sheath showing DNA trapped inside the cement tube. Microphotograph of a sheath from SJArg-2-Nit, magnifying on the right side a scan stack of the cement, trapping nuclei shown some with gray arrows. Inset: bar chart showing the different number of host nuclei observed/captured by scanning at ∼1 µm stacks in (*a*) SJArg-2-Nit sheaths (*N*_nits_ = 7) and (*b*) SJArg-1-Nit (*N*_nits_ = 6). DNA captured with DAPI fluorescent signal.

For SJArg-1-Nit and SJArg-2-Nit, confocal microscopy analysis showed preservation of cuticle of hair with a similar quality to that of a fresh, healthy hair, when covered by cement. However, for SJArg-1-Nit, cuticle scales showed signs of deterioration, for example, being broken or detached in the cement-uncovered areas and showing rupture reaching the cortex or the medulla. Whereas SJArg-2-Nit showed much better preservation of the hair outside this segment, despite being about 500 years older. In addition, we noticed that the cuticle types of SJArg-1-Nit hair were different in shape, with large variation in the arrangement and size of the scales (supplementary fig. S1, Supplementary Material online). Host nuclei were only recorded on the inside of the wrapping tube of cement (fig. 2, supplementary fig. S1, Supplementary Material online, and Materials and Methods). Host nuclei trapped in cement were highly positive with two fluorescent DNA dyes, DAPI or propidium iodide, and showed the typical regular and flattened shape (size of ∼8–12 µm) (Neelam et al. 2016). Of the two mummies, SJArg-2-Nit presented abundant nuclei trapped in the cement, whereas these were rarer for SJArg-1-Nit. Counts of nuclei from sheath specimens of each mummy were significantly different (fig. 2 inset), indicating a variation in the number of nuclei trapped inside the cement of the nit. Nits collected from the older SJArg-2-Nit mummy showed an average of five nuclei per nit sheath, whereas only up to one per nit cement was counted for nits from SJArg-1-Nit specimens. Additionally, at the time of collection of samples we noted that SJArg-2-Nit mummy had likely suffered from dandruff, indicated by the profuse shedding of scalp skin.

Across all samples, we were unable to identify mitochondria in scans due to the limited magnification of confocal scanning microscopy, despite fully scanning the diameter of the hair and comparing between the sections covered by cement with the cement-free sections of the hair surface. None of the cement-free sections of the scanned shafts were positive for the fluorescent stains. Inside the cement, signals were observed from trapped material, such as dirt, likely bacterial, or fungal cells (based on shape). These particles, together with the shedding skin cells were collected or retained inside the liquid cement when it was secreted by the mother louse to glue the new egg to the hair, providing additional protection not only to the DNA of host cells, but to potentially also cells and extracellular DNA from other organisms.

### DNA Sequencing

**Fig. 3.**
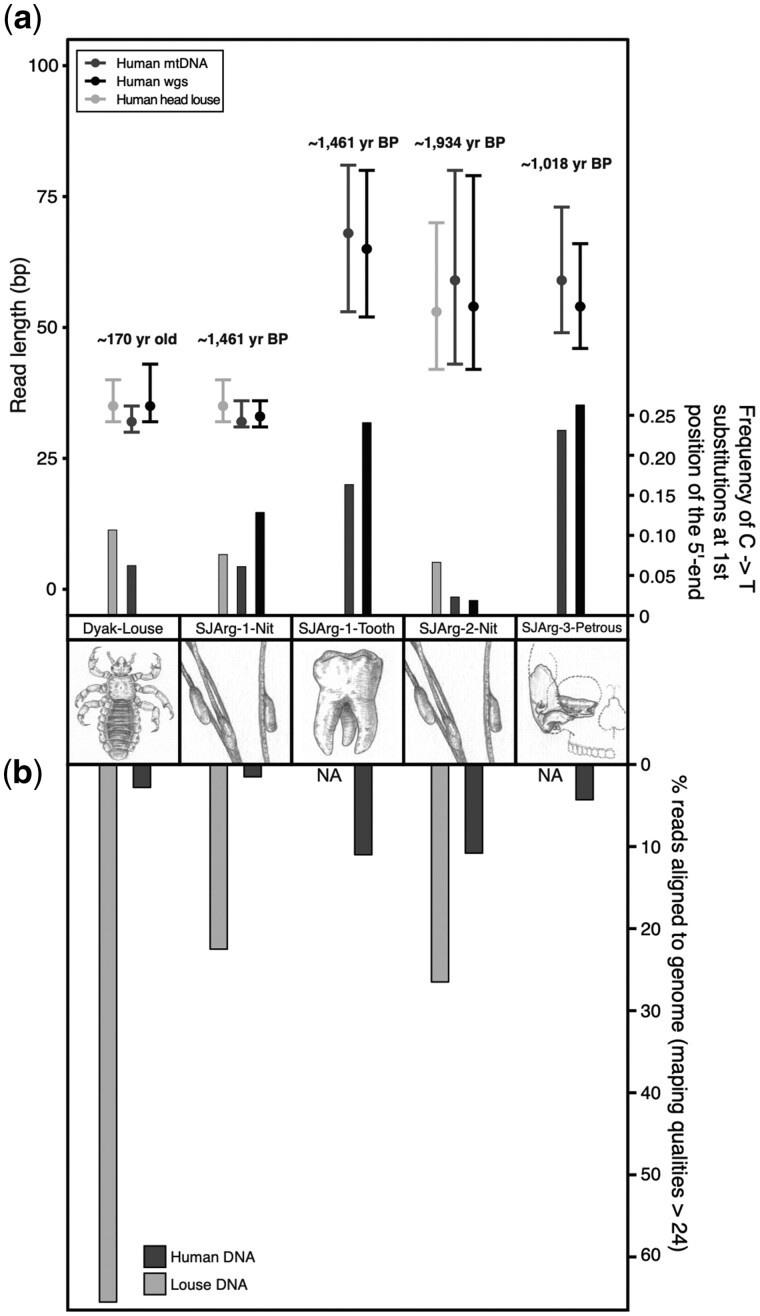
DNA properties: (*a*) upper part showing box plots of read lengths of reads aligned to the human mitochondrial DNA (mtDNA), the whole genome (wgs), and the louse genome (*Pediculus*), dots are medians and bars present minimum and maximum range [yr BP: Median values, calibrated years Before Present, details in supplementary table S1, Supplementary Material online]; (*b*) lower part: DNA damage from reads aligning to the respective genomes are plotted as bars. Comparison of the proportion of reads aligning to the human and the head louse genome with mapping qualities 25 or higher (see Materials and Methods).

We extracted and sequenced ∼285 million (raw) reads, which yielded 21 million reads upon trimming, duplicate removal, and quality filtering (supplementary table S7, Supplementary Material online, and Materials and Methods). All reads were mapped against the human genome build 37 and resulted in between 1.6% and 11% of the reads aligning for the five different samples (fig. 3, supplementary table S7, Supplementary Material online, and Materials and Methods). We found a mean coverage across the nuclear genome of 0.04×, 0.006×, 0.3×, 0.1×, and 0.09× for the Dyak-Louse, SJArg-1-Nit, SJArg-1-Tooth, SJArg-2-Nit, and SJArg-3-Petrous samples, respectively. Across the mitochondrial genome, for Dyak-Louse, SJArg-1-Nit, SJArg-1-Tooth, SJArg-2-Nit, and SJArg-3-Petrous samples, we found a mean coverage of 0.1×, 1.7×, 29.9×, 0.8×, and 3.9× respectively (fig. 3, supplementary table S7, Supplementary Material online, and Materials and Methods). To test for endogenous content, we compared these results with the amount of retrieved louse DNA from both the louse and nit cement by mapping all reads against the human body louse (*P. humanus corporis* assembly GCA_000006295.1, in absence of a human head louse genome [Kirkness et al. 2010]). Louse DNA was more abundant in each of the samples, with 65.2% of the total reads for the Dyak-Louse aligning. For SJArg-Nit-1 and SJArg-Nit-2, it yielded 22.5% and 26.5% (supplementary table S7, Supplementary Material online, and Materials and Methods).

### Ancient DNA Authentication

The above results indicate that endogenous human DNA was preserved and extracted from all samples, however, we still sought to confirm its ancient origin by calculating the proportion of nucleotide misincorporations arising from DNA damage for aligning to both the human and louse genome using mapDamage (Jónsson et al. 2013; see supplementary information, Supplementary Material online). We found no intra-sample variation in the fragmentation and amount of DNA damage accumulated between the louse and human DNA. We also found no relationship between the age of a sample and the amount of accumulated DNA damage. In contrast, the 170 years-old Dyak-Louse exhibited relatively high DNA damage (∼0.06 C->T frequency, fig. 3), similar to that found in the almost 1,500 year older SJArg-1-Nit (1,461 cal. BP). We also found the DNA damage to be highly elevated in the SJArg-3-Petrous and SJArg-1-Tooth compared with all other samples. These remains were exposed to the elements and not inside a cave as SJArg-2-Nit, they were found under a terrace; the terrace location of SJArg-1-Tooth is shown in supplementary figure S2, Supplementary Material online. The DNA damage of SJArg-2-Nit (∼1,934 cal. BP) is low for both human and louse DNA (∼0.02 and ∼0.06, respectively). We therefore calculated the length of all reads aligning to both genomes for each sample, as a measure of the degree of fragmentation as well as a check for introduction of longer modern contamination by human DNA (fig. 3 and supplementary figs. S11–S23, Supplementary Material online). We found no difference in mean length of the reads between the louse and human DNA within each sample, and therefore, no indication for contamination with modern DNA.

### Contamination Estimates

We used contamMix (Fu et al. 2013) to quantify the proportion of mtDNA contamination in the sequencing data (supplementary figs. S11–S23, Supplementary Material online). We estimated low levels of contamination for SJArg-1-Tooth (0.07%, 95% CI: 0.01–1.46%, supplementary fig. S3, Supplementary Material online), SJArg-3-Petrous (1.29%, 95% CI: 0.15–7.53%, supplementary fig. S4, Supplementary Material online), and SJArg-1-Nit (4.02%, 95% CI: 1.33–9.34%, supplementary fig. S5, Supplementary Material online). In contrast, contamination estimates were larger for SJArg-2-Nit (14.57%, 95% CI: 6.61–28.62%, supplementary fig. S6, Supplementary Material online) and Dyak-Louse (62.69%, 95% CI: 12.4–92.25%, supplementary fig. S7, Supplementary Material online). It was noted that the latter estimates are associated with wide confidence intervals caused by low average depth of coverage across the mtDNA (0.8× for SJArg-2-Nit and 0.1× for Dyak-Louse). Importantly, all American individuals were found to belong to haplogroup A2, suggesting that additional data would likely result in lower contamination estimates. As such, we consider that broad ancestry investigation of these individuals is not likely to be substantially affected by contamination.

### Chromosomal Sex Determination, Mitochondrial and Y Chromosome Haplogroup

The fraction of Y chromosome alignments compared with the total number of alignments to the X and Y chromosome was used to determine the chromosomal sex of each sample (Skoglund et al. 2013) (supplementary fig. S7, Supplementary Material online). The archaeological excavation records dating back over 60 years indicated the sex of just two specimens, SJArg-2-Nit to be male (XY) and SJArg-3-Petrous female (XX). The sex of the Dyak-Louse host was also unknown. The genetic analysis showed SJArg-1-Tooth to be XX while SJArg-1-Nit has a ratio (R_y_) similar to males. We then calculated the fraction of Y chromosome in the extraction blank to ensure that the SJArg-1-Nit results were not due to background contamination and found the extraction blank to be female. Therefore, we concluded that the male proportion of the SJArg-1-Nit was not caused by contamination from laboratory reagents. The analysis also confirmed SJArg-3-Petrous to be XX and SJArg-2-Nit XY, and revealed Dyak-Louse host as XY.

We next sought to determine the Y chromosomal haplogroup. Although the coverage for Dyak-Louse and SJArg-1-Nit were found too low for calling the haplotypes, SJArg-2-Nit (Y-coverage 0.038×) was characterized as Q1b1a1a1 or Q-M3 (M848), which today is the largest Native American haplogroup (carried by 90%, supplementary table S8, Supplementary Material online). It is, however, important to note that this call is based on four SNPs of which three are transitions and the final SNP is at the third position from the end of a read. But considering SJArg-2-Nits, low degree of DNA damage adds more confidence to SNPs based on transitions. Lastly, we determined the mitochondrial haplogroups for each of the samples using HaploGrep2.0 and found all SJArg samples to be A2+ (64). The Dyak/Dayak lice were collected from an individual in Sarawak, Northwest Kalimantan Dayak, Sea Dayak, or Iban (Simonson et al. 2011). Despite the low coverage, it was possible to place the bloodmeal of the Dyak-Louse in the H2a2a haplogroup.

### Genome–Wide Affinities

**Fig. 4.**
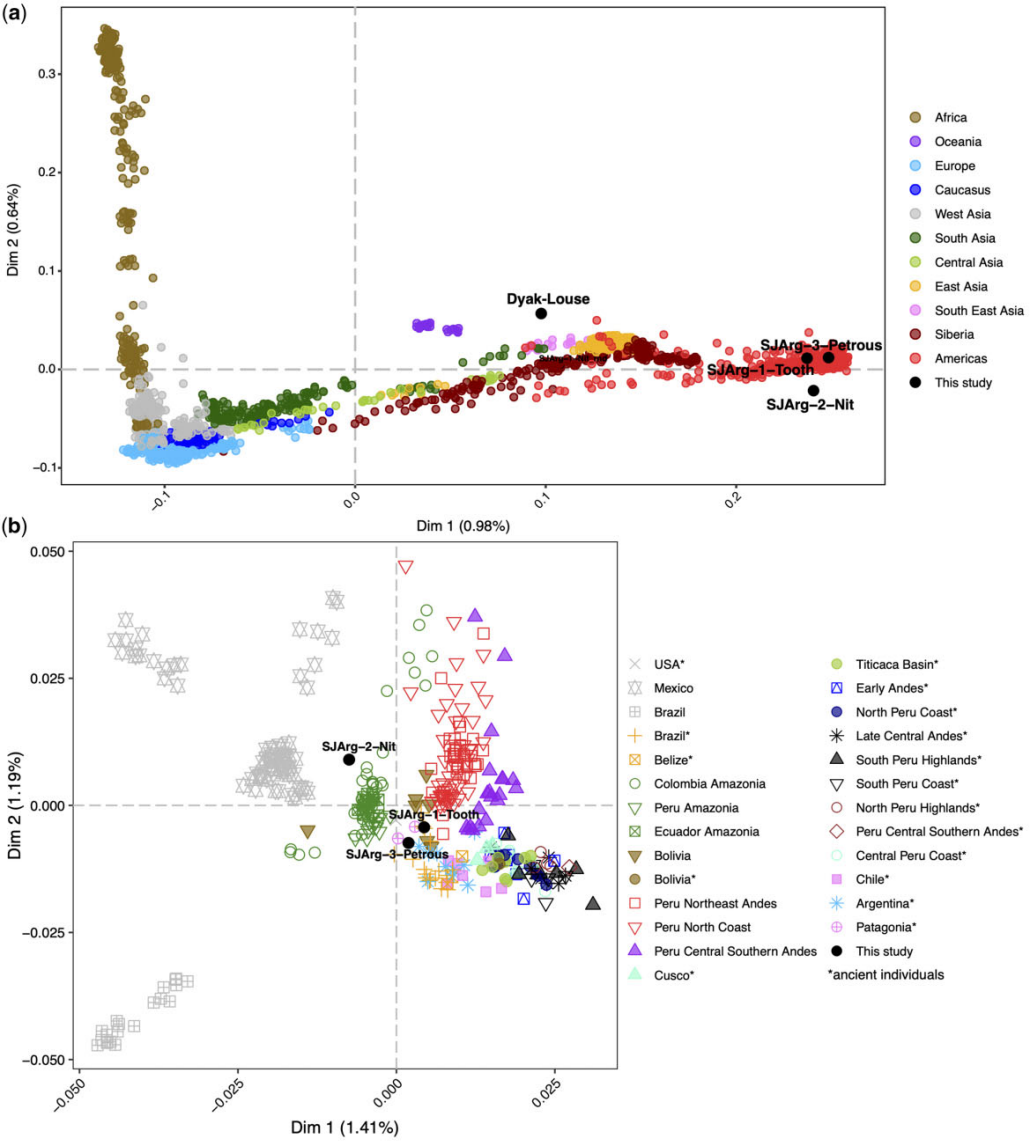
MDS plots of nuclear DNA of (*a*) worldwide present-day and ancient individuals projecting the samples from this study and (*b*) the indigenous American genetic diversity. Samples: Dyak-Louse, SJArg-1-Tooth, SJArg-2-Nit, and SJArg-3-Petrous in bold.

We used multidimensional scaling (MDS) to investigate the broad population genetic affinities of the ancient individuals (fig. 4*a*). We merged the genome data from the Dayak-Louse and the three best Argentinean samples (SJArg-1-Tooth, SJArg-2-Nit, and SJArg-3-Petrous) with a genotype panel containing data from 2,462 worldwide present-day and ancient individuals genotyped for 621,194 single-nucleotide polymorphisms (Lazaridis et al. 2014; Posth et al. 2018; Barbieri et al. 2019; Nakatsuka et al. 2020). To minimize potential batch effects associated with coanalyzing SNP-array, SNP-capture, and whole-genome sequencing data, we considered pseudohaploid genotypes for every individual in the data set (Malaspinas et al. 2014). The number of sites with nonmissing data for each individual were 775, 165,559, 7,975, and 54,078 for Dyak-Louse, SJArg-1-Tooth, SJArg-2-Nit, and SJArg-3-Petrous, respectively. In the MDSs, the Dyak-Louse is placed with Asian populations (fig. 4*a*). In accordance with the archaeological and historical record, the three Argentinean individuals with the highest genome coverage (SJArg-1-Tooth, SJArg-2-Nit, and SJArg-3-Petrous) were placed within Indigenous American genetic diversity (fig. 4*b*). Although SJArg-1-Nit was included in an initial MDS analysis (not shown) and was placed close to Asian, we deem the results for this individual too noisy to derive a meaningful interpretation due to its low genome coverage. We confirmed these results by carrying out a principal components analysis (PCA) where the ancient low-coverage samples were projected onto the genotype reference data set (Patterson et al. 2006) (supplementary fig. S8, Supplementary Material online). Next, we assessed the particular genetic affinities of the three individuals with the highest genome coverage using a subset of the reference data set restricted to Indigenous American populations (present and ancient, fig. 4*b*). In this case, the three individuals clustered separately from North and Central American individuals and are placed together with the Southern Cone individuals (fig. 4*b*). Interestingly, the younger specimens SJArg-3-Petrous and SJArg-1-Tooth are placed slightly separated from the older SJArg-2-Nit. More data would be necessary to achieve greater resolution, we emphasize that these results are based on low coverage data from a unique substrate.

### Ancient Environmental DNA

Lastly, we parsed all reads from both nits and the louse sample through the metagenomic classifier Holi (Pedersen et al. 2016) to taxonomically classify reads not assigned to human or louse (Materials and Methods). For this, it was specified that each taxon should have similar DNA damage to the louse and human DNA in each sample (0.07 for Dyak-Louse and SJArg-1-Nit, and 0.03 for SJArg-2-Nit), parsing only taxa with >50 reads to that respective taxonomic level. We found overall a low complexity for all samples with a few abundant taxa, in addition to the human and louse DNA (supplementary fig. S9, Supplementary Material online, and Materials and Methods). In both the Dyak-Louse and SJArg-2-Nit, lice endosymbiotic bacteria, *Candidatus* Riesia (Enterobacteriaceae), were found with ∼79% and ∼28% of the reads classified, respectively. *Ca.* Riesia was not found in SJArg-1-Nit, instead, the bacterial class Actinobacteria was found to be most abundant (>70% of all reads) with more than 50% assigned at the family level Nocardiosaceae or lower; together with the intestinal associated Enterococcus where 5% of the reads were assigned. In the Dyak-Louse, we also identified ∼800 reads (0.7%) assigned to the fruit fly *Rhagoletis* sp. In the SJArg-2-Nit sample, we found 9% of the reads assigned to the commensal bacterial genus *Proteus* which holds several opportunistic pathogenic species including *P. mirabilis*. Interestingly, we also found 115 reads (0.5%) uniquely assigned to the cancerogenic Merkel cell polyomavirus MCPyV (supplementary fig. S9, Supplementary Material online), known to cause an aggressive form of skin cancer (Pietropaolo et al. 2020). To further verify the identification of MCPyV, we computed genome-wide statistics, and found 74% of the genome are covered with a mean depth of 1.4× and with an average read length similar to that found in the human and louse reads.

### Louse DNA Mitochondrial Haplotypes

**Fig. 5.**
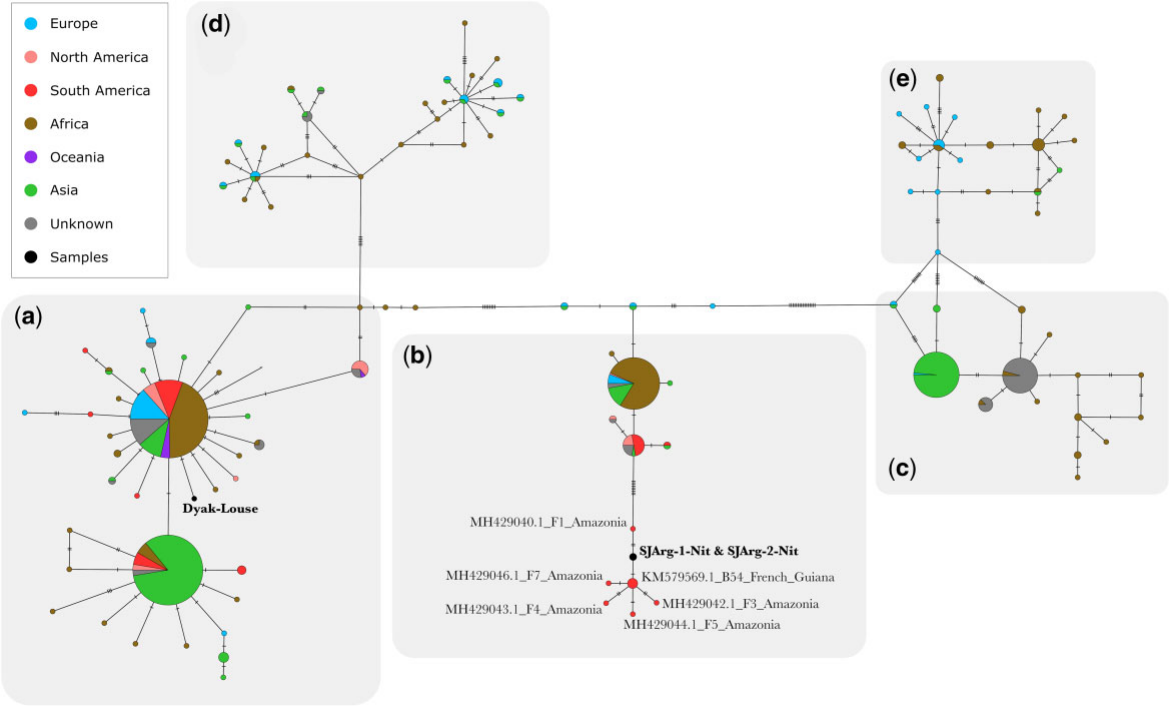
Louse (*Pediculus humanus*) cytochrome *b* haplotype network showing that the two nit samples from mummies (in bold) belong to a mitochondrial network that, even with present-day samples still has South American members only. The Dyak-Louse (in bold) is part of a group distributed world-wide. Haplotypes A–E are given in bold, black capitalized letters.

To investigate the placement of the louse/nits DNA among populations worldwide, we mapped isolated louse reads from SJArg-1-Nit, SJArg-2-Nit, and Dyak-Louse independently to two consensus sequences retrieved from GenBank references (supplementary table S9, Supplementary Material online). These are the mitochondrial cytochrome *b* and COI genes of human head and body lice. We then compared the resultant sequences to the reference data set for each gene and built two haplotype networks. For the cytochrome *b* gene, we found that SJArg-1-Nit and SJArg-2-Nit fall within the haplogroup B, more specifically they are part of a distinct South American cluster consisting of lice exclusively collected from the Amazon and French Guiana (fig. 5). In our cytochrome *b* analysis, the Dyak-Louse is part of the haplogroup A, which is found worldwide. When building the consensus sequence for the COI gene, a greater number of low quality and poorly mapped reads were removed and this introduced long stretches of N’s, making it impossible to confidently place our samples within the COI reference data set. We therefore excluded the COI haplotype network. The genetic analysis of lice matches the morphological and taxonomic study of the nits. These nits belong to *Pediculus* lice of Amerindians as well as Peruvian mummies.

## Discussion

We report here evidence that ancient human, pathogen, and environmental DNA can be obtained from cells trapped in the cement of nits. This allowed us to determine the human chromosomal sex of the samples, revealing the sex of the host of SJArg-1-Nit and Dyak-Louse samples to be XY, and the mummy SJArg-1-Tooth to be XX, thus providing valuable information that was not recorded upon sampling. All our South American individuals were found to belong to mitochondrial haplogroup A2, consistent with previous studies that show A2 to be one of a few founding maternal lineages in the Americas. Furthermore, we found that the three individuals with highest genome coverage clustered separately from North and Central American individuals and closest to the Southern Cone individuals. Our historical sample from the Dyak/Dayak/Dayuh individual falls consistently with Asian which is consistent with its geographical location, Borneo. Lastly, we demonstrate that cement also traps cells or extracellular DNA from the human microbiome and virome, and by analyzing lice mtDNA, we verified that they accurately mirror their human host history.

Aside from gaining human and louse genetic information, nit samples can offer unique details about a number of circumstances surrounding the life and death of their hosts. We established that these mummies showed an unusual pattern in the location of the last nits deposited, that can only be explained by exposure to very low, cold ambient temperature at the time of death. Short distances, 2–4 mm to the root are observed in high frequencies for SJ-Arg-4-Nit and SJ-Arg-1-Nit. Previous studies on lice biology confirm that the last oviposited head louse nits are always glued at a minimum average of 5 mm from the scalp (∼7 mm from the root), hereby exposed at an ideal temperature for development (Buxton 1940; Leeson 1941; Busvine 1948; Lang 1975; Maunder 1983; Lambiase and Perotti 2019). This is the case for SJ-Arg-2-Nit, where most of the nits were glued at ∼7 mm from the root and beyond (supplementary tables S5 and S6, Supplementary Material online). This suggests that death likely happened during a winter of extremely low temperatures (Gambier 1977) for SJArg-1-Nit and SJArg-4-Nit hosts, whereas SJ-Arg-2 seems to have died of natural causes and not due to extreme temperatures. We also identified bacterial species that can be opportunistic pathogens but ubiquitous too. More importantly, we found that SJArg-2-Nit carried Merkel cell polyomavirus (MCPyV, Human Polyomavirus 5, a dsDNA virus), which currently provides the earliest direct evidence for this human viral pathogen. MCPyV was only discovered in 2008. It is normally shed by healthy adult human skin. The virus can very rarely integrate into the human genome and may cause aggressive skin cancer, known as Merkel cell carcinoma (Feng et al. 2008). It is uncertain whether the virus was present on the hair or on the louse cementing the egg. Although cord blood shows a seroprevalence of up to 90% (Karachaliou et al. 2016), by age 3 or 4, the prevalence has dropped to 30–40% (Cason et al. 2018), from where MCPyV spreads most readily in children at the very age when hair lice infestations are more frequent (Martel-Jantin et al. 2013; van der Meijden et al. 2013). This opens up the possibility that head lice might be a mechanical vector for MCPyV in addition to other routes of infection. A multitude of infection routes is characteristic for evolutionary recent host-pathogen associations.

Notably, nit sample SJArg-2-Nit contains a similar proportion of host human DNA to the tooth, and more than two times what was found in the petrous bone, whereas the proportions of louse DNA were also found to be similar between both nit samples ∼22.5–26.5%. Differences in the total content of DNA between samples can arise from different rates of degradation due to environmental variability, such as temperature, UV light exposure, and humidity. In the four South American samples from Argentina, SJArg-1,2-Nit, SJArg-1-Tooth, and SJArg-3-Petrous, we observed a high variability between the amount of DNA damage (fig. 3), where damage is found two and ten times higher in the tooth and the petrous, compared with that in nits. This suggests that DNA incorporated into the proteinaceous cement (Park et al. 2019) is better protected from deamination processes. In contrast, we find the average DNA read length of SJArg-1-Nit (a hair specimen detached from an unknown individual) to be 50% shorter than for the other South American samples which could be explained by taphonomic processes. This preservation difference in the DNA is further corroborated by the confocal microscopy scanning of hairs containing nits, which showed that the ∼500 year older individual, SJArg-2-Nit, was in better condition than the younger SJArg-1-Nit. This is likely due to the condition and position of the remains at the archaeological site. SJArg-2-Nit was found as a complete mummified body, in excellent preservation condition deeper inside a cave, whereas hair sample SJArg-1-Nit was located in an exposed rock shelter (open atrium, not a cave), and therefore endured exposure to more harsh environmental conditions of the Cordillera de los Andes. Previous work on hair preservation of Chihuahua mummies from caves (∼830 cal. years BP) concluded that over time the cuticle scale pattern of head hair becomes irregularly disordered and distorted (Mansilla et al. 2011), similar to the patterns observed in the hair of SJArg-1-Nit (supplementary information and supplementary fig. S1, Supplementary Material online). The same study compared hair from ancient mummies to that of modern humans, who presented a smooth and regular arrangement of the scales (Mansilla et al. 2011), almost identical to the pattern shown in the hair of our SJArg-2-Nit mummy (supplementary fig. S1, Supplementary Material online), which is over 1,000 years older that the Chihuahua ones.

We found the proportion of host DNA from the gut of the Dyak-Louse sample to be lower than that in nit cements. The DNA yield of host DNA from the adult lice was low, especially considering that the endogenous louse DNA content was high (65.5%). There can be multiple reasons for the poor DNA yield. The first reason might be that the bloodmeal is already at an advanced stage of physiological digestion. The second, the conditions in museums which often keep entomological collections at room temperature and occasionally use pesticides (Espeland et al. 2010). The third, which is the most parsimonious explanation, is that the small amount of bloodmeal contains few cells with nuclear DNA (Lord et al. 1998; Mumcuoglu et al. 2004). Both lice and nits not only provided lice genomic data but also retrieved the endosymbiotic bacteria of lice (Perotti et al. 2004, 2006, 2007, 2008; Allen et al. 2007; Allen et al. 2009). In spite of the low coverage, the Dyak/Dayak/Dayuh individual was found to belong to H2a2a haplogroup, which fits well with B4 + 16262 (H2): 16182C, 16183C, 16189C, 16217C, 16261T, and 16519C reported from South Kalimantan Dayak, now a minor haplotype shared with a frequency of 0.04688 with Malagasy and Indonesian populations of Borneo (Kusuma et al. 2015).

Using the human mitochondrial information of the nits, we showed that haplotyping can be carried out from minute samples such as six nit sheaths. For both SJArg-1-Nit and SJArg-2-Nit, we found that they belong to the mitochondrial haplogroup A2 (+64), and the same haplogroup was assigned to the tooth and the petrous bone. This is one of the main founder haplogroups of the Americas (Scheib et al. 2018), and has been characterized from other remains of similar age, mid to late Holocene, in both Peru and north-west Argentina (Coutinho et al. 2014; Postillone et al. 2017). SJ-Arg-2-Nit is a complete mummified body, found in good condition inside a cave; whereas SJ-Arg-1-Nit belong to a hair sample found in a rock shelter close by. The fact that they all carry the same haplotype, despite the difference in both locality and time, informs us of a continuum in the region’s maternal lineage. This could be explained by matrilocal practices that have also been suggested from previous kinship analyses of ancient local populations in north-west Argentina during the later pre-Columbian period (Mendisco et al. 2018). At present, haplogroup A2 and A2+(64) are prevalent in the modern populations with native ancestry from the north of Chile and Argentina to Bolivia and Peru (Taboada-Echalar et al. 2013; Llamas et al. 2016; Harris et al. 2018). It is still unresolved whether A2 or A2+ (64) is ancestral, A2 might be a back mutation. Although A2+ (64) is almost absent from native Patagonians south of Chile and Argentina (Vidal et al. 2019; Postillone et al. 2020), it interestingly shows a decreasing latitudinal gradient from 9% in the north to 1% toward the south of Chile (Gomez-Carballa et al. 2016). Archaeological and genetic studies of sites in NW and Central Western Region of Argentina (CWA) suggest that the region was characterized by great mobility of temporary settlers and travelers, which could explain the fragmented occupancy of the shelters in this study, only separated by a few hundreds of years (Gambier 1977; Postillone et al. 2017; Russo et al. 2017). Both the mitochondrial A2 haplogroup and the SJArg-2-Nit Y chromosome haplogroup (Q-M3 [M848]) are the largest Native South American haplogroups. They are highly prevalent in isolated, almost unknown native populations of Amazonian Ecuador, Colombia, and Peru that have only been included or studied recently (Barbieri et al. 2019). From a genome-wide population perspective, all three genomes, SJArg-1-Tooth, SJArg-2-Nit, and SJArg-3-Petrous, center around the Americas (MDS, fig. 4*a*). Interestingly, the analysis of their genetic affinities with present and ancient American genomes (MDS, fig. 4*b*) places the older specimen SJArg-2-Nit (∼2,000 years BP) together with present-day individuals from Peruvian, Ecuadorian, and Colombian Amazonia, away from the 500-year younger tooth and petrous specimens, which cluster with Argentinean, Patagonian ancient samples and with present Bolivians. These results should be considered cautiously and could have been caused by a combination of low genome coverage and intrapopulation variability. However, the genome-wide analysis confirms the journey of the mitochondrial haplogroups. In southern South America, different ancient migration routes were already suggested by Gambier (1977). Recent studies provided strong linguistic and genetic evidence of a major migration wave, occurring around 2,000 years BP from the ancient Proto Tupi/Tupí of the Amazonian lowlands (northwest Amazon) toward south Amazonia and the east Coast, spanning ∼4,000 km (Silva Noelli 2008; Castro et al. 2020). One of the hypotheses explaining this move is based on the premise that Proto Tupis/Tupís (considered agriculturalists and ceramists) reached a demographic bottleneck, requiring migrations toward new lands for agriculture (Castro et al. 2020); whereas others propose that it was in response to a changing drier climate at that time (Bustamante et al. 2016).

In the latest genetic analyses using current time populations from the same region of this study, Calingasta (San Juan, Argentina), Luisi et al. (2020) showed the greatest genetic affinity with people on the Pacific coast of central Chile. Current Sanjuaninos are unrelated to the original Huarpes, and are of a very recent ancestry, traced back to two or three generations only. In general, most remote native populations of Argentina are underrepresented in genetic studies, as the indigenous people were eradicated by the 20th century. In the early 1800s, all native Huarpes of San Juan were “deported” to Chile, due to the need for an indigenous workforce in Santiago de Chile (Michieli 1983). Luisi et al. (2020) have shown that the lack of ancient samples from CWA, specifically San Juan province, prevents resolution of the origin of their indigenous people. The three Calingasta ancient mummies analyzed in this study show for the first time that the original peoples of San Juan, Calingasta, came from Amazonia about 2,000 years BP—SJArg-2-Nit, and relate to Patagonian ancient samples about 1,500-1,000 years BP—SJArg-1-Tooth. Therefore, the DNA trapped in the nit cement of SJArg-2-Nit, has helped decipher a piece of the puzzle of pre-Columbian ancient migrations within South America, of an impressive move spanning 5,000–6,500 km.

The cytochrome *b* gene network constructed with the Dyak-Louse sample locates these lice as *P.humanus capitis* from South East Asia, within the haplogroup A, of worldwide distribution and highly prevalent in Asia and Africa. Remarkably, the network for the two ancient nit samples from Argentina, SJArg-1-Nit and SJArg-2-Nit, traces back their ancestry to *P. humanus capitis* from the Amazon lowlands, the same location found as for the human host DNA: the mitochondrial haplogroup analyses and MDS analysis for SJArg-2-Nit. A pre-Columbian origin of this human louse clade has been proposed and that it may be prevalent in the indigenous American lice populations (Amanzougaghene et al. 2019). Currently, Wayampi/Wayãpi Amerindians from southwest of French Guinea, and Argentineans (central Argentina) plus Mexicans carry lice with the same cytochrome *b* gene haplotype, which branches from Clade B, and is now recognized as a new, separate Clade F (Amanzougaghene et al. 2019). According to linguistic phylogenies, Wayampi/Wayãpi Amerindians are direct descendants of the original Tupi/Tupí of the Amazonia, who extensively migrated ∼2,000 years BP toward the south Amazonia lowlands (Silva Noelli 2008). The genetic analysis of the nits are confirmed by their taxonomic and morphological study, matching *P.humanus* nits already identified in the early 1900s by Ewing from hair of indigenous Americans and of a few ancient mummies from Peru (Ewing 1933). The absence of the primary, obligatory symbiont of lice, *Ca.* Riesia in specimen SJArg-1-Nit, whereas being abound in SJArg-2-Nit, can be either due to the bad preservation status of this mummy, or due to the use of very young louse embryos, which carry only an incipient basket mycetome with a handful of Riesia cells (Perotti et al. 2007).

This study shows for the first time that ancient nit sheaths provide a reliable source of host and nonhost genomic DNA. The DNA of the host is trapped inside the cement secreted by the female louse to attach the eggs to the hair, which becomes a sealant preserving the DNA for at least 2,000 years. Most human mummies carry nits attached to their hair, offering a ubiquitous and alternative, nondestructive source of aDNA, that becomes particularly relevant when teeth or petrous bones are not available. It also shows that hair samples without a skeleton can still provide mitochondrial and nuclear DNA through nits. This work demonstrates that nit samples allow genetic characterizations of ancient hosts, despite the limitations due to preservation of the remains, which are compensated by extra information from the analyses of lice biology, opening a small window into their past life and the environment in which they lived.

## Materials and Methods

### Specimen Sampling

Sampling of nits consisted of pulling off a handful of hairs from the preserved scalp of the mummies, except for SJArg-1-Nit, which was a specimen of hair detached from an unknown individual.

Cytogenetic analyses were conducted to verify the content of DNA inside the cement tube of five and seven nits attached to hair, from SJArg-1-Nit (∼1,461 [median] ±131 cal. BP) and SJArg-2-Nit (∼1,934 [median] ± 625 cal. BP), respectively.

For whole genome-wide studies of the DNA trapped in sheaths’ cement, only six nits from each specimen SJArg-1-Nit and SJArg-2-Nit were available for this work, and the nits cut off the hair. The remaining few nits attached to the hair of the specimens were left untouched for future research. One tooth, SJArg-1-Tooth (∼1568 ± 24 cal. years BP), originating from the same layer of the rock shelter than SJArg-1-Nit, and one petrous bone, SJArg-3-Petrous (∼1,018 ± 249 cal. years BP) from the same archaeological site were used for whole genome sequencing, and to validate the quality of the DNA obtained for sheath cement. Sample Dyak-Louse corresponds to two adult lice that were pooled for the DNA extraction, and the resulting genetic analyses were also used for comparison with the genome-wide data obtained from the cells trapped in sheath cement. We also checked for the presence of DNA in the fractions at both ends of the sheath attachments on shafts and did not detect any DNA in hair shafts. This was checked using DNA binding dyes and performing DNA extractions that resulted in no DNA. The lack of DNA in shafts can be also visualized in figure 2 and supplementary figure S1, Supplementary Material online.

For morphological measures on hair with sheaths, we considered the following specimens (Supplementary Material online): Samples of three Argentinian mummies, SJArg-1-Nit, SJArg-2-Nit, and SJArg-4-Nit (∼1,192 ± 370 cal. years BP), 10, 14 and 20 nits, respectively, were measured in the museum, in situ, over the head of each mummy for distance of nits to scalp, which were the last oviposited nits before death of the host. The length of sheath cement was measured on SJArg-1-Nit 5 nits, SJArg-2-Nit 7 nits, SJArg-4-Nit 8 nits, SJArg-5-Nit 3 nits (∼590 years BP), SJArg-6-Nit 2 nits (∼880 years BP), and SJArg-7-Nit 3 nits (590 years BP), plus Chi-8-Nit 5 nits (∼300 years BP) and Chi-9-Nit 3 nits (∼400 years BP) at Reading University.

Detailed information on the data procedures and statistical analyses of morphometric data (cement length and distance of nit from scalp) are found in the Supplementary Material online, and data in supplementary tables S3 and S5, Supplementary Material online.

### Sample Origin, Ethics, and Permits

Permits and clearance for conducting morphological and genetic studies on ancient remains for international transport and for destructive sampling (when/where required) were obtained before the start date (list of certificates and permits in Supplementary Material online). We collected minute fragments of ancient hair containing nits, a tooth, and a petrous bone available from collections at the Instituto de Investigaciones Arqueológicas y Museo “Prof. Mariano Gambier” (San Juan, Argentina) and at the Museo Chileno de Arte Precolombino (Santiago de Chile). The specimens of Argentina originated in Calingasta Caves, San Juan province, and Argentina were assigned to the Ansilta culture. The samples from Chile (both used for morphometrics), one was from a textile object made of human hair, originated in North of Atacama, Chile, having no cultural association; the other specimen from the Amazonian Andes of Ecuador, from a shrunken human head of the Jivaroan peoples. Adult lice were sourced from Denny’s historical Anoplura collection (OUNHM, Oxford University), which dates ∼100 years BP, and the two specimens were from one human host, a Dyak/Dayak/Dayuh native of Borneo (Sarawak). Supplementary table S2, Supplementary Material online, includes the catalogue numbers for each specimen as in the collections of origin.

### Radiocarbon Dating

At the time of the finding of the mummies in the 1900s, radiocarbon dating was performed on the sediments where each of the remains was found (supplementary table S1, Supplementary Material online), thus, dating the corresponding mummy by its association to the relevant sedimentary layer/and/or burial. To test the accuracy of this original dating, which was performed over 50 years ago, mummy SJ-Arg-1 was re-dated for this study at the SUERC. For these analyses, hair fragments from SJ-Arg-1-nit were used, and after hand picking under a microscope and cleaning, gave a radiocarbon age of 1,568 ± 24 (this study; 421–547 cal AD, 95.4% probability). The calibration was undertaken using OxCal v4.3.2 (Bronk Ramsey 2017) using IntCal20.14c atmospheric curve (Reimer et al. 2020). This radiocarbon age is very close to the original dating by association of 1,580 ± 60 years BP from the bulk sediments (Gambier 1977).

### Cytogenetics and Confocal Fluorescence Microscopy

Six dry nits attached to hair from each of the two specimens, SJ-Arg-1-Nit and SJ-Arg-2-Nit were processed for cytogenetic analysis to detect the presence of DNA, like nuclei trapped inside the cement tube. The samples were re-hydrated and overnight incubated in 500 µl of PBS after which they were placed in 500 µl of methanol/heptane (1:1) for 2 min whilst shaking in order to fenestrate the vitelline membrane of the nits. They were then washed with cold methanol twice and kept in it for 24 h at 4 °C for fixation. The methanol was replaced with chilled (−20 °C) acetone and left for 1 min. This was followed by double washing with room temperature PBS and incubation for 15 min in PBST (with 1% Triton X-100). PBST was replaced with PBS (3% BSA + 0.1% Tween) and incubated at room temperature for 1 h. Double washing with room temperature PBS was repeated followed by overnight staining with either propidium iodide or DAPI (4′,6-diamidino-2-phenylindole) (10 µg/ml). A final double wash with PBS was carried out followed by mounting in glycerol/PBS (v:v, 1:1). The samples were observed with a Zeiss LSM 710 (inverted) confocal microscope using a 561-nm laser and filter for propidium iodide, and UV laser and filter for DAPI. All images were acquired and analyzed with the associated software Zen 3.1.

### DNA Extraction, Libraries, and Sequencing

aDNA work was performed in dedicated clean laboratory facilities at the Lundbeck Foundation Centre for GeoGenetics, GLOBE institute, University of Copenhagen.

### DNA Preparation of Nits and Lice

Each set of nits including cement or lice were transferred to a low-bind Eppendorf tube and incubated in a 5% bleach solution for 5 min to remove potential surface contaminants. The bleach was hereafter pipetted out, and samples washed twice in 500 μl lysis buffer (10 mM Tris/HCI, 140 mM NaCI, 3 mM CaCl_2_, 50 mM DTT, 1% SDS, 0.1 mg Proteinase K), pH 8.0, at 37 °C (Gilbert et al. 2007). All samples were then added 400 µl of lysis buffer and incubated gently rotating overnight at 37 °C. The following day, the samples were added to the ∼0.5 g lysis matrix E and disrupted using a MP FastPrep—in order to aid the breakdown of the remaining structures. Additional 0.1 mg of proteinase K was added to each sample and incubated rotating overnight. The samples were then mixed with 8 ml of binding buffer (2 M guanidine hydrochloride, 70% vol/vol isopropanol, 0.05% Tween 20) (Glocke and Meyer 2017) and spun through a High Pure Viral Nucleic Acid Large Volume Kit spin column at 5,000 g for 2 min. The flow through was hereafter removed, and the filters washed with 750 µl Qiagen PE buffer and spun down for 2 min at 5,000 g. The flow through was removed and the residual PE buffer on the filter was removed by spinning for 2 min at 5,000 g. DNA was then eluted in 32 µl Qiagen EB buffer, and the concentration measured on a Qubit2.0 following the manufacturer’s protocol. All DNA was then converted to Illumina libraries using the method as outlined previously (Meyer and Kircher 2010; Pedersen et al. 2016) and sequenced on an Illumina HiSeq4000 platform 80 cycles, single-read.

### DNA Preparation of the Tooth and Petrous Bone

Cementum-enriched material (∼140 mg) was sampled from a single well-preserved molar (SJArg-1-Tooth) and the inner dense material from the petrous bone (SJArg-3-Petrous) and extracted for aDNA following standard protocol (Damgaard et al. 2015; Hansen et al. 2017). DNA libraries were hereafter constructed as according to Meyer and Kircher (2010), using unique dual indexing, and sequenced on a HiSeq4000 platform (Illumina) 80 cycles single read.

### Bioinformatic Processing

After sequencing, all reads were demultiplexed and trimmed using AdapterRemoval v2 (Schubert et al. 2016) and a total of 285 million reads between 10 million and 108 million reads for the five different samples and 0.5 million for the extraction blank were hereafter mapped to the human reference genome build 37 using BWA align using default parameters (Li and Durbin 2009). Reads with mapping quality lower than 25 were discarded using SAMtools view (-25) and followed by all PCR duplicates removed using SAMtools rmdup (Li and Durbin 2009). For each individual, we called pseudo-haploid genotypes by randomly sampling a single read at every SNP site in the reference data set. We called an “*N*” whenever a sampled base gave rise to a triallelic site.

### Ancient DNA Authentication

We examined the fragment length distributions and the base substitution patterns using mapDamage (Jónsson et al. 2013) and estimated the mtDNA quality using contamMix (Fu et al. 2013) (following Moreno-Mayar et al. [2018]). Damage and fragmentation estimates for all samples are found in supplementary figures S11–S23, Supplementary Material online.

### Population Structure Analyses

The broad relationships between ancient and present-day genomes were investigated using MDS applied to the identity-by-state-distance matrix (bammds: 10.1093/bioinformatics/btu410), and PCA, where ancient low-depth data were projected onto the reference data set (Patterson et al. 2006).

### Mitochondrial DNA and Y-Chromosome Haplotyping

Haploid variants were called using the endoCaller program implemented in Schmutzi60 and only variants with a posterior probability exceeding 50 on the PHRED scale (probability of error: 1/100,000) were retained. We then used Haplogrep v2.261 to determine the mtDNA haplogroup, specifying PhyloTree (build 17) as the reference phylogeny. Y-chromosome variant calling and placement were performed following Pinotti et al. (2019) and according to the International Society of Genetic Genealogy 2020 Y-DNA Haplogroup tree.

### Environmental DNA and Analysis

We next performed a metagenomic analysis by taxonomically classifying all reads following the Holi pipeline (Pedersen et al. 2016), excluding reads classified as human or louse. All taxa with 50 or more reads were hereafter extracted and parsed through mapDamage2.0 for DNA damage estimates. We then filtered out all taxa with C->T frequencies less than (-0.02 mor more) than found in the human and louse reads. All taxa and read counts were hereafter parsed to R and plotted using ggplot2 (supplementary fig. S9, Supplementary Material online).

### Louse DNA Mitochondrial Haplotyping

We mapped reads that were classified as lice using bowtie2 v.2.3.2 (Langmead and Salzberg 2012) to two consensus sequences that we obtained from a downloaded reference data set of human head and body lice for the mitochondrial gene cytochrome *b* and COI from GenBank (accession numbers, supplementary table S9, Supplementary Material online) with the software Geneious Prime v.2020.0.5(Biomatters, Ltd., Auckland, New Zealand). We then quality filtered the resultant bam files specifying a minimum mapping quality of 25 using SAMtools (Li and Durbin 2009; Li et al. 2009) and built a consensus for each sample with angsd (Korneliussen et al. 2014), specifying a minimum read depth of 5, a base quality threshold of 25 and removing duplicates. Haplotype minimum spanning networks were generated using PopART (Leigh and Bryant 2015).

## Supplementary Material

Supplementary data are available at *Molecular Biology and Evolution* online.

## Supplementary Material

msab351_Supplementary_Data

## References

[msab351-B1] Alexander JO. 1984. Arthropods and human skin. Suffolk (United Kingdom): Springer-Verlag.

[msab351-B2] Allen JM , Light JE, Perotti MA, Braig HR, Reed DL. 2009. Mutational meltdown in primary endosymbionts: selection limits Muller’s ratchet. PLoS One 4(3):e4969. 19305500 10.1371/journal.pone.0004969 PMC2654755

[msab351-B3] Allen JM , Reed DL, Perotti MA, Braig HR. 2007. Evolutionary relationships of “*Candidatus* Riesia spp.”, endosymbiotic Enterobacteriaceae living within hematophagous primate lice. Appl Environ Microbiol. 73(5):1659–1664. 17220259 10.1128/AEM.01877-06 PMC1828778

[msab351-B4] Amanzougaghene N , Fenollar F, Davoust B, Djossou F, Ashfaq M, Bitam I, Raoult D, Mediannikov O. 2019. Mitochondrial diversity and phylogeographic analysis of *Pediculus humanus* reveals a new Amazonian clade “F”. Infect Genet Evol. 70:1–8. 30769089 10.1016/j.meegid.2019.02.006

[msab351-B5] Araujo A , Ferreira LF, Guidon N, Freire NMD, Reinhard KJ, Dittmar K. 2000. Ten thousand years of head lice infection. Parasitol Today. 16(7):269–269. 10858638 10.1016/s0169-4758(00)01694-x

[msab351-B6] Arriaza B , Cartmell LL, Moragas C, Nerlich AG, Salo W, Madden M, Aufderheide C. 2008. The bioarchaeological value of human mummies without provenience. Chungará 40(1):55–65.

[msab351-B7] Arriaza B , Orellana NC, Barbosa HS, Menna-Barreto RFS, Araujo A, Standen V. 2012. Severe head lice infestation in an Andean mummy of Arica, Chile. J Parasitol. 98(2):433–436. 22010860 10.1645/GE-2903.1

[msab351-B8] Arriaza B , Standen VG, Heukelbach J, Cassman V, Olivares F. 2014. Head combs for delousing in ancient Arican populations: scratching for the evidence. Chungará 46(4):693–706.

[msab351-B9] Barbieri C , Barquera R, Arias L, Sandoval JR, Acosta O, Zurita C, Aguilar-Campos A, Tito-Alvarez AM, Serrano-Osuna R, Gray RD, et al 2019. The current genomic landscape of western South America: Andes, Amazonia, and Pacific Coast. Mol Biol Evol. 36(12):2698–2713. 31350885 10.1093/molbev/msz174 PMC6878948

[msab351-B10] Bronk Ramsey C. 2017. Methods for summarizing radiocarbon datasets. Radiocarbon 59(6):1809–1833.

[msab351-B11] Burkhart CN , Arbogast J, Smythe P, Burkhart CG. 1999. Histochemical analysis of the nit of *Pediculus humanus capitis* (Anoplura: Pediculidae). J Med Entomol. 36(4):530–532. 10467785 10.1093/jmedent/36.4.530

[msab351-B12] Burkhart CN , Burkhart CG. 2005. Head lice: scientific assessment of the nit sheath with clinical ramifications and therapeutic options. J Am Acad Dermatol. 53(1):129–133. 15965432 10.1016/j.jaad.2005.01.134

[msab351-B13] Bustamante MG , Cruz FW, Vuille M, Apaéstegui J, Strikis N, Panizo G, Novello FV, Deininger M, Sifeddine A, Cheng H, et al 2016. Holocene changes in monsoon precipitation in the Andes of NE Peru based on δ18O speleothem records. Quatern Sc Rev. 146:274–287.

[msab351-B14] Busvine JR. 1948. The head and body races of *Pediculus humanus* L. Parasitology 39(1–2):1–16. 18876872 10.1017/s0031182000083505

[msab351-B15] Buxton PA. 1940. Temperatures lethal to the louse. Br Med J. 1(4130):341. 20782978 10.1136/bmj.1.4130.341 PMC2176476

[msab351-B16] Capasso L , Di Tota G. 1998. Lice buried under the ashes of Herculaneum. Lancet 351(9107):992. 9734976

[msab351-B17] Cason C , Monasta L, Zanotta N, Campisciano G, Maestri I, Tommasino M, Pawlita M, Villani S, Comar M, Delbue S. 2018. Antibody response to polyomavirus primary infection: high seroprevalence of Merkel cell polyomavirus and lymphoid tissue involvement. J Neurovirol. 24(3):314–322. 29330826 10.1007/s13365-017-0612-2

[msab351-B18] Castro ESMA , Nunes K, Lemes RB, Mas-Sandoval A, Guerra Amorim CE, Krieger JE, Mill JG, Salzano FM, Bortolini MC, Pereira ADC, et al 2020. Genomic insight into the origins and dispersal of the Brazilian coastal natives. Proc Natl Acad Sci U S A. 117(5):2372–2377. 31932419 10.1073/pnas.1909075117 PMC7007564

[msab351-B19] Claw KG , Lippert D, Bardill J, Cordova A, Fox K, Yracheta JM, Bader AC, Bolnick DA, Malhi RS, TallBear K, et al 2017. Chaco Canyon dig unearths ethical concerns. Hum Biol. 89(3):177–180. 29745246 10.13110/humanbiology.89.3.01 PMC5951383

[msab351-B20] Coutinho A , Valverde G, Fehren-Schmitz L, Cooper A, Barreto Romero MI, Espinoza IF, Llamas B, Haak W. 2014. AmericaPlex26: a SNaPshot multiplex system for genotyping the main human mitochondrial founder lineages of the Americas. PLoS One 9(3):e93292. 24671218 10.1371/journal.pone.0093292 PMC3966882

[msab351-B21] Curry CJ , Davis BW, Bertola LD, White PA, Murphy WJ, Derr JN. 2021. Spatiotemporal genetic diversity of lions reveals the influence of habitat fragmentation across Africa. Mol Biol Evol. 38(1):48–57. 32667997 10.1093/molbev/msaa174 PMC8480188

[msab351-B22] Damgaard PB , Margaryan A, Schroeder H, Orlando L, Willerslev E, Allentoft ME. 2015. Improving access to endogenous DNA in ancient bones and teeth. Sci Rep. 5:11184. 26081994 10.1038/srep11184 PMC4472031

[msab351-B23] de la Cruz KD , Ribbeck R, Daugschies A. 2003. Palaeoparasitological analysis of guinea pig mumies of the Chiribaya Culture, Moquegua Valley, Peru. Berl Münch Tierärztl Wochenschr. 116(1–2):45–49. 12592929

[msab351-B24] Dittmar K. 2009. Old parasites for a new world: the future of paleoparasitological research. A review. J Parasitol. 95(2):365–371. 18702568 10.1645/GE-1676.1

[msab351-B25] Dittmar K. 2014. Palaeoparasitology and ancient DNA. In: Ferreira LF, Reinhard KJ, Araújo A, editors. Foundations of Paleoparasitology. Rio de Janeiro (Brazil): FIOCRUZ. p. 277–288.

[msab351-B26] Dittmar K , Mamat U, Whiting M, Goldmann T, Reinhard K, Guillen S. 2003. Techniques of DNA-studies on prehispanic ectoparasites (*Pulex* sp., Pulicidae, Siphonaptera) from Animal mummies of the Chiribaya culture, southern Peru. Mem Inst Oswaldo Cruz. 98(Suppl 1):53–58. 12687763 10.1590/s0074-02762003000900010

[msab351-B27] Durden LA , Kessler SE, Boundenga L, Ngoubangoye B, Tsoumbou TA, Moussadji-Kinga CI, Halbwax M, Setchell JM, Nichols J, Greiman SE. 2020. A new npecies of sucking louse from the mandrill from Gabon with a review of host associations and geographical distributions, and identification keys to members of the genus *Pedicinus* (Phthiraptera: Anoplura: Pedicinidae). J Parasitol. 106(2):221–232. 32164028 10.1645/19-170

[msab351-B28] Dutra JMF , Alves AD, Pessanha T, Rachid R, de Souza W, Linardi PM, Ferreira LF, de Souza SM, Araujo A. 2014. Prehistorical *Pediculus humanus capitis* infestation: quantitative data and low vacuum scanning microscopy. Rev Inst Med Trop Sao Paulo. 56(2):115–119. 24626412 10.1590/S0036-46652014000200005 PMC4085847

[msab351-B29] Eberhard M. 2002. Bird collections - an essential resource for collecting ectoparasites, in particular chewing lice. Bonn Zool Beitr. 51(2/3):131–135.

[msab351-B30] Eichler W. 1971. Shaking of bird skins as a Mallophaga collecting method. Angew Parasitol. 12(1):38–52. 5153138

[msab351-B31] Espeland M , Irestedt M, Johanson KA, Akerlund M, Bergh JE, Kallersjo M. 2010. Dichlorvos exposure impedes extraction and amplification of DNA from insects in museum collections. Front Zool. 7:2. 20148102 10.1186/1742-9994-7-2 PMC2819063

[msab351-B32] Ewing HE. 1924. Lice from human mummies. Science 60(1556):389–390. 17836983 10.1126/science.60.1556.389

[msab351-B33] Ewing HE. 1926. A revision of the American lice of the genus *Pediculus*, together with a consideration of the significance of their geographical and host distribution. Proc US Nat Museum. 68(2620):1–30.

[msab351-B34] Ewing HE. 1933. The taxonomy of the anopluran genus *Pediculus* Linnaeus. Proc Biol Soc Wash. 46:167–174.

[msab351-B35] Ewing HE. 1938. The sucking lice of American monkeys. J Parasitol. 24(1):13–33.

[msab351-B36] Feldman M , Harbeck M, Keller M, Spyrou MA, Rott A, Trautmann B, Scholz HC, Paffgen B, Peters J, McCormick M, et al 2016. A high-coverage *Yersinia pestis* genome from a sixth-century Justinianic plague victim. Mol Biol Evol. 33(11):2911–2923. 27578768 10.1093/molbev/msw170 PMC5062324

[msab351-B37] Feng H , Shuda M, Chang Y, Moore PS. 2008. Clonal integration of a polyomavirus in human Merkel cell carcinoma. Science 319(5866):1096–1100. 18202256 10.1126/science.1152586 PMC2740911

[msab351-B38] Ferris GF. 1951. The sucking lice. Mem Pac Coast Entomol Soc. 1:1–320.

[msab351-B39] Fox K , Hawks J. 2019. Use ancient remains more wisely. Nature 572(7771):581–583. 31462783 10.1038/d41586-019-02516-5

[msab351-B40] Fu Q , Mittnik A, Johnson PLF, Bos K, Lari M, Bollongino R, Sun C, Giemsch L, Schmitz R, Burger J, et al 2013. A revised timescale for human evolution based on ancient mitochondrial genomes. Curr Biol. 23(7):553–559. 23523248 10.1016/j.cub.2013.02.044 PMC5036973

[msab351-B41] Gambier M. 1977. La Cultura de Ansilta. In: Gambier M, editor. La Cultura de Ansilta. San Juan (Argentina): Instituto de Investigaciones Arqueológicas y Museo, Universidad Nacional de San Juan. p. 3–166.

[msab351-B42] Gazda MA , Toomey MB, Araujo PM, Lopes RJ, Afonso S, Myers CA, Serres K, Kiser PD, Hill GE, Corbo JC, et al 2020. Genetic basis of de novo appearance of carotenoid ornamentation in bare parts of canaries. Mol Biol Evol. 37(5):1317–1328. 31930402 10.1093/molbev/msaa006

[msab351-B43] Geigl EM , Grange T. 2018. Ancient DNA: the quest for the best. Mol Ecol Resour. 18(6):1185–1187. 30375193 10.1111/1755-0998.12931

[msab351-B44] Gilbert MT , Tomsho LP, Rendulic S, Packard M, Drautz DI, Sher A, Tikhonov A, Dalen L, Kuznetsova T, Kosintsev P, et al 2007. Whole-genome shotgun sequencing of mitochondria from ancient hair shafts. Science 317(5846):1927–1930. 17901335 10.1126/science.1146971

[msab351-B45] Glocke I , Meyer M. 2017. Extending the spectrum of DNA sequences retrieved from ancient bones and teeth. Genome Res. 27(7):1230–1237. 28408382 10.1101/gr.219675.116 PMC5495074

[msab351-B46] Gomez-Carballa A , Moreno F, Alvarez-Iglesias V, Martinon-Torres F, Garcia-Magarinos M, Pantoja-Astudillo JA, Aguirre-Morales E, Bustos P, Salas A. 2016. Revealing latitudinal patterns of mitochondrial DNA diversity in Chileans. Forensic Sci Int Genet. 20:81–88. 26517175 10.1016/j.fsigen.2015.10.002

[msab351-B47] Hansen HB , Damgaard PB, Margaryan A, Stenderup J, Lynnerup N, Willerslev E, Allentoft ME. 2017. Comparing ancient DNA preservation in petrous bone and tooth cementum. PLoS One 12(1):e0170940. 28129388 10.1371/journal.pone.0170940 PMC5271384

[msab351-B48] Harris DN , Song W, Shetty AC, Levano KS, Caceres O, Padilla C, Borda V, Tarazona D, Trujillo O, Sanchez C, et al 2018. Evolutionary genomic dynamics of Peruvians before, during, and after the Inca Empire. Proc Natl Acad Sci U S A. 115(28):E6526–E6535. 29946025 10.1073/pnas.1720798115 PMC6048481

[msab351-B49] Hofreiter M , Poinar HN, Spaulding WG, Bauer K, Martin PS, Possnert G, Paabo S. 2000. A molecular analysis of ground sloth diet through the last glaciation. Mol Ecol. 9(12):1975–1984. 11123610 10.1046/j.1365-294x.2000.01106.x

[msab351-B50] Huchet JB , Callou C, Lichtenberg R, Dunand F. 2013. The dog mummy, the ticks and the louse fly: archaeological report of severe ectoparasitosis in Ancient Egypt. Int J Paleopathol. 3(3):165–175. 29539451 10.1016/j.ijpp.2013.07.001

[msab351-B51] Jensen TZT , Niemann J, Iversen KH, Fotakis AK, Gopalakrishnan S, Vagene AJ, Pedersen MW, Sinding MS, Ellegaard MR, Allentoft ME, et al 2019. A 5700 year-old human genome and oral microbiome from chewed birch pitch. Nat Commun. 10(1):5520. 31848342 10.1038/s41467-019-13549-9 PMC6917805

[msab351-B52] Jónsson H , Ginolhac A, Schubert M, Johnson PL, Orlando L. 2013. mapDamage2.0: fast approximate Bayesian estimates of ancient DNA damage parameters. Bioinformatics 29(13):1682–1684. 23613487 10.1093/bioinformatics/btt193 PMC3694634

[msab351-B53] Karachaliou M , Waterboer T, Casabonne D, Chalkiadaki G, Roumeliotaki T, Michel A, Stiakaki E, Chatzi L, Pawlita M, Kogevinas M, et al 2016. The natural history of human polyomaviruses and herpesviruses in early life–The Rhea Birth Cohort in Greece. Am J Epidemiol. 183(7):671–679. 26968942 10.1093/aje/kwv281

[msab351-B54] Kirkness EF , Haas BJ, Sun W, Braig HR, Perotti MA, Clark JM, Lee SH, Robertson HM, Kennedy RC, Elhaik E, et al 2010. Genome sequences of the human body louse and its primary endosymbiont provide insights into the permanent parasitic lifestyle. Proc Natl Acad Sci U S A. 107(27):12168–12173. 20566863 10.1073/pnas.1003379107 PMC2901460

[msab351-B55] Kittler R , Kayser M, Stoneking M. 2003. Molecular evolution of *Pediculus humanus* and the origin of clothing. Curr Biol. 13(16):1414–1417. 12932325 10.1016/s0960-9822(03)00507-4

[msab351-B56] Korneliussen TS , Albrechtsen A, Nielsen R. 2014. ANGSD: analysis of next generation sequencing data. BMC Bioinformatics. 15:356. 25420514 10.1186/s12859-014-0356-4 PMC4248462

[msab351-B57] Koungoulos L , Contos P. 2019. Global expansion of the Australian biting louse *Heterodoxus spiniger* facilitated by human transport of dog (*Canis familiaris*), and implications for prehistoric cultural interaction in Australasia. Environ Archaeol. 24:1–14.

[msab351-B58] Kusuma P , Cox MP, Pierron D, Razafindrazaka H, Brucato N, Tonasso L, Suryadi HL, Letellier T, Sudoyo H, Ricaut FX. 2015. Mitochondrial DNA and the Y chromosome suggest the settlement of Madagascar by Indonesian sea nomad populations. BMC Genomics. 16:191. 25880430 10.1186/s12864-015-1394-7 PMC4373124

[msab351-B59] Lambiase S , Perotti MA. 2019. Using human head lice to unravel neglect and cause of death. Parasitology 146(5):678–684. 30526723 10.1017/S0031182018002007

[msab351-B60] Lang JD. 1975. Biology and control of the head louse, *Pediculus humanus capitis* (Anoplura: Pediculidae), in a semi-arid urban area [Doctor in Philosophy]. Tucson (AZ): University of Arizona.

[msab351-B61] Langmead B , Salzberg SL. 2012. Fast gapped-read alignment with Bowtie 2. Nat Methods. 9(4):357–359. 22388286 10.1038/nmeth.1923 PMC3322381

[msab351-B62] Lazaridis I , Patterson N, Mittnik A, Renaud G, Mallick S, Kirsanow K, Sudmant PH, Schraiber JG, Castellano S, Lipson M, et al 2014. Ancient human genomes suggest three ancestral populations for present-day Europeans. Nature 513(7518):409–413. 25230663 10.1038/nature13673 PMC4170574

[msab351-B63] Leeson HS. 1941. The effect of temperature upon the hatching of the eggs of *Pediculus humanus corporis* de Geer (Anoplura). Parasitology 33(2):243–249.

[msab351-B64] Leigh JW , Bryant D. 2015. POPART: full-feature software for haplotype network construction. Methods Ecol Evol. 6(9):1110–1116.

[msab351-B65] Li H , Durbin R. 2009. Fast and accurate short read alignment with Burrows-Wheeler transform. Bioinformatics 25(14):1754–1760. 19451168 10.1093/bioinformatics/btp324 PMC2705234

[msab351-B66] Li H , Handsaker B, Wysoker A, Fennell T, Ruan J, Homer N, Marth G, Abecasis G, Durbin R; 1000 Genome Project Data Processing Subgroup. 2009. The sequence alignment/map format and SAMtools. Bioinformatics 25(16):2078–2079. 19505943 10.1093/bioinformatics/btp352 PMC2723002

[msab351-B67] Linardi PM , De Maria M, Botelho JR, Cunha HC, Ferreira JB. 1988. Prevalence of nits and lice in samples of cut hair from floors of barbershops and beauty parlors in Belo Horizante, Minas Gerais State, Brazil. Mem Inst Oswaldo Cruz. 83(4):471–474. 10.1590/s0074-02761988000400013 3271944

[msab351-B68] Llamas B , Fehren-Schmitz L, Valverde G, Soubrier J, Mallick S, Rohland N, Nordenfelt S, Valdiosera C, Richards SM, Rohrlach A, et al 2016. Ancient mitochondrial DNA provides high-resolution time scale of the peopling of the Americas. Sci Adv. 2(4):e1501385. 27051878 10.1126/sciadv.1501385 PMC4820370

[msab351-B69] Lord WD , DiZinno JA, Wilson MR, Budowle B, Taplin D, Meinking TL. 1998. Isolation, amplification, and sequencing of human mitochondrial DNA obtained from human crab louse, *Pthirus pubis* (L.), blood meals. J Forensic Sci. 43(5):1097–1100. 9729835

[msab351-B70] Luisi P , Garcia A, Berros JM, Motti JMB, Demarchi DA, Alfaro E, Aquilano E, Arguelles C, Avena S, Bailliet G, et al 2020. Fine-scale genomic analyses of admixed individuals reveal unrecognized genetic ancestry components in Argentina. PLoS One 15(7):e0233808. 32673320 10.1371/journal.pone.0233808 PMC7365470

[msab351-B71] Lynnerup N. 2015. The Thule Inuit mummies from Greenland. Anat Rec. 298(6):1001–1006. 10.1002/ar.23131 25998634

[msab351-B72] Makarewicz C , Marom N, Bar-Oz G. 2017. Ensure equal access to ancient DNA. Nature 548(7666):158. 10.1038/548158a 28796208

[msab351-B73] Malaspinas AS , Tange O, Moreno-Mayar JV, Rasmussen M, DeGiorgio M, Wang Y, Valdiosera CE, Politis G, Willerslev E, Nielsen R. 2014. bammds: a tool for assessing the ancestry of low-depth whole-genome data using multidimensional scaling (MDS). Bioinformatics 30(20):2962–2964. 24974206 10.1093/bioinformatics/btu410 PMC4184259

[msab351-B74] Mansilla J , Bosch P, Menéndez MT, Pijoan C, Flores C, López M. D C, Lima E, Leboreiro I. 2011. Archaeological and comtemporary human hair composition and morphology. Chungará 43(2):293–302.

[msab351-B75] Martel-Jantin C , Pedergnana V, Nicol JTJ, Leblond V, Trégouët D-A, Tortevoye P, Plancoulaine S, Coursaget P, Touzé A, Abel L, et al 2013. Merkel cell polyomavirus infection occurs during early childhood and is transmitted between siblings. J Clin Virol. 58(1):288–291. 23829968 10.1016/j.jcv.2013.06.004

[msab351-B76] Massilani D , Skov L, Hajdinjak M, Gunchinsuren B, Tseveendorj D, Yi S, Lee J, Nagel S, Nickel B, Deviese T, et al 2020. Denisovan ancestry and population history of early East Asians. Science 370(6516):579–583. 33122380 10.1126/science.abc1166

[msab351-B77] Maunder JW. 1983. The appreciation of lice. Proc R Inst G Br. 55:1–32.

[msab351-B78] Mendisco F , Keyser C, Seldes V, Nielsen AE, Russo MG, Crubézy E, Ludes B. 2018. An insight into the burial practices of the late pre-Hispanic Los Amarillos community (northwestern Argentina) through the study of ancient DNA. J Archaeol Res. 91:12–19.

[msab351-B79] Meyer M , Kircher M. 2010. Illumina sequencing library preparation for highly multiplexed target capture and sequencing. Cold Spring Harb Protoc. 2010(6):pdb prot5448. 20516186 10.1101/pdb.prot5448

[msab351-B80] Michieli CT. 1983. Los Huarpes protohistóricos [The proto-historic Huarpes]. San Juan (Argentina): Inst. de Investigaciones Arqueológicas y Museo.

[msab351-B81] Moreno-Mayar JV , Potter BA, Vinner L, Steinrucken M, Rasmussen S, Terhorst J, Kamm JA, Albrechtsen A, Malaspinas AS, Sikora M, et al 2018. Terminal Pleistocene Alaskan genome reveals first founding population of Native Americans. Nature 553(7687):203–207. 29323294 10.1038/nature25173

[msab351-B82] Mumcuoglu KY , Gallili N, Reshef A, Brauner P, Grant H. 2004. Use of human lice in forensic entomology. J Med Entomol. 41(4):803–806. 15311479 10.1603/0022-2585-41.4.803

[msab351-B83] Nakatsuka N , Lazaridis I, Barbieri C, Skoglund P, Rohland N, Mallick S, Posth C, Harkins-Kinkaid K, Ferry M, Harney E, et al 2020. A paleogenomic reconstruction of the deep population history of the Andes. Cell 181(5):1131–1145.e1121. 32386546 10.1016/j.cell.2020.04.015 PMC7304944

[msab351-B84] Neelam S , Hayes PR, Zhang Q, Dickinson RB, Lele TP. 2016. Vertical uniformity of cells and nuclei in epithelial monolayers. Sci Rep. 6:19689. 26795751 10.1038/srep19689 PMC4726213

[msab351-B85] Nieves-Colón MA , Pestle WJ, Reynolds AW, Llamas B, de la Fuente C, Fowler K, Skerry KM, Crespo-Torres E, Bustamante CD, Stone AC. 2020. Ancient DNA reconstructs the genetic legacies of precontact Puerto Rico communities. Mol Biol Evol. 37(3):611–626. 31710665 10.1093/molbev/msz267

[msab351-B86] O’Rourke DH , Hayes MG, Carlyle SW. 2000. Ancient DNA studies in physical anthropology. Annu Rev Anthropol. 29(1):217–242.

[msab351-B87] Ozga AT , Nieves-Colon MA, Honap TP, Sankaranarayanan K, Hofman CA, Milner GR, Lewis CM Jr, Stone AC, Warinner C. 2016. Successful enrichment and recovery of whole mitochondrial genomes from ancient human dental calculus. Am J Phys Anthropol. 160(2):220–228. 26989998 10.1002/ajpa.22960 PMC4866892

[msab351-B88] Palsdottir AH , Blauer A, Rannamae E, Boessenkool S, Hallsson JH. 2019. Not a limitless resource: ethics and guidelines for destructive sampling of archaeofaunal remains. R Soc Open Sci. 6(10):191059. 31824712 10.1098/rsos.191059 PMC6837180

[msab351-B89] Panagiotakopulu E , Higham T, Sarpaki A, Buckland P, Doumas C. 2013. Ancient pests: the season of the Santorini Minoan volcanic eruption and a date from insect chitin. Naturwissenschaften 100(7):683–689. 23793358 10.1007/s00114-013-1068-8

[msab351-B90] Panagiotakopulu E , Skidmore P, Buckland P. 2007. Fossil insect evidence for the end of the Western Settlement in Norse Greenland. Naturwissenschaften 94(4):300–306. 17216429 10.1007/s00114-006-0199-6

[msab351-B91] Park JK , Han YJ, Lee JH, Joo SW, Kim JH, Lee SH, Park S. 2019. Characterization of the human head louse nit sheath reveals proteins with adhesive property that show no resemblance to known proteins. Sci Rep. 9(1):48. 30631086 10.1038/s41598-018-36913-z PMC6328571

[msab351-B92] Patterson N , Price AL, Reich D. 2006. Population structure and eigenanalysis. PLoS Genet. 2(12):e190. 17194218 10.1371/journal.pgen.0020190 PMC1713260

[msab351-B93] Pedersen MW , Ruter A, Schweger C, Friebe H, Staff RA, Kjeldsen KK, Mendoza MLZ, Beaudoin AB, Zutter C, Larsen NK, et al 2016. Postglacial viability and colonization in North America's ice-free corridor. Nature 537(7618):45–49. 27509852 10.1038/nature19085

[msab351-B94] Perotti MA , Allen JM, Reed DL, Braig HR. 2007. Host-symbiont interactions of the primary endosymbiont of human head and body lice. FASEB J. 21(4):1058–1066. 17227954 10.1096/fj.06-6808com

[msab351-B95] Perotti MA , Catalá SS, Ormeño A. D V, Zelazowska M, Biliński SM, Braig HR. 2004. The sex ratio distortion in the human head louse is conserved over time. BMC Genet. 5:10. 15140268 10.1186/1471-2156-5-10 PMC428572

[msab351-B96] Perotti MA , Clarke HK, Turner BD, Braig HR. 2006. Rickettsia as obligate and mycetomic bacteria. FASEB J. 20(13):2372–2374. 17012243 10.1096/fj.06-5870fje

[msab351-B97] Perotti MA , Kirkness EF, Reed DL, Braig HR. 2008. Endosymbionts of lice. In: B Kostas, T Miller, editors. Insect symbiosis. Vol. III. New York: CRC. p. 205–219.

[msab351-B98] Pietropaolo V , Prezioso C, Moens U. 2020. Merkel cell polyomavirus and Merkel cell carcinoma. Cancers 12(7):1774. 10.3390/cancers12071774 PMC7407210 32635198

[msab351-B99] Pilli E , Vai S, Caruso MG, D’Errico G, Berti A, Caramelli D. 2018. Neither femur nor tooth: petrous bone for identifying archaeological bone samples via forensic approach. Forensic Sci Int. 283:144–149. 29301114 10.1016/j.forsciint.2017.12.023

[msab351-B100] Pinhasi R , Fernandes DM, Sirak K, Cheronet O. 2019. Isolating the human cochlea to generate bone powder for ancient DNA analysis. Nat Protoc. 14(4):1194–1205. 30842617 10.1038/s41596-019-0137-7

[msab351-B101] Pinotti T , Bergström A, Geppert M, Bawn M, Ohasi D, Shi W, Lacerda DR, Solli A, Norstedt J, Reed K, et al 2019. Y chromosome sequences reveal a short Beringian standstill, rapid expansion, and early population structure of Native American founders. Curr Biol. 29(1):149–157. 30581024 10.1016/j.cub.2018.11.029

[msab351-B102] Poinar HN , Schwarz C, Qi J, Shapiro B, Macphee RD, Buigues B, Tikhonov A, Huson DH, Tomsho LP, Auch A, et al 2006. Metagenomics to paleogenomics: large-scale sequencing of mammoth DNA. Science 311(5759):392–394. 16368896 10.1126/science.1123360

[msab351-B103] Ponce de Leon MS , Koesbardiati T, Weissmann JD, Milella M, Reyna-Blanco CS, Suwa G, Kondo O, Malaspinas AS, White TD, Zollikofer CPE. 2018. Human bony labyrinth is an indicator of population history and dispersal from Africa. Proc Natl Acad Sci U S A. 115(16):4128–4133. 29610337 10.1073/pnas.1717873115 PMC5910833

[msab351-B104] Posth C , Nakatsuka N, Lazaridis I, Skoglund P, Mallick S, Lamnidis TC, Rohland N, Nagele K, Adamski N, Bertolini E, et al 2018. Reconstructing the deep population history of Central and South America. Cell 175(5):1185–1197.e1122. 30415837 10.1016/j.cell.2018.10.027 PMC6327247

[msab351-B105] Postillone MB , Fuchs ML, Crespo CM, Russo MH, Varela HH, Carnese FR, Avena SA, Dejean CB. 2017. Linajes maternos en muestras antiguas de la Puna Jujeña. Comparación con estudios de la región centro-sur andina [Maternal lineages in ancient samples from Puna Jujeña. Comparisons with studies in the Central-South Andean region]. Rev Argent Antropol Biol. 19:7–22.

[msab351-B106] Postillone MB , Martinez G, Flensborg G, Dejean CB. 2020. First analysis of mitochondrial lineages from the eastern Pampa-Patagonia transition during the final late Holocene. Am J Phys Anthropol. 171(4):659–670. 32017021 10.1002/ajpa.24016

[msab351-B107] Prendergast ME , Sawchuk E. 2018. Boots on the ground in Africa's ancient DNA ‘revolution’: archaeological perspectives on ethics and best practices. Antiquity 92(363):803–815.

[msab351-B108] Raoult D , Reed DL, Dittmar K, Kirchman JJ, Rolain JM, Guillen S, Light JE. 2008. Molecular identification of lice from pre-Columbian mummies. J Infect Dis. 197(4):535–543. 18254682 10.1086/526520

[msab351-B109] Reed DL , Light JE, Allen JM, Kirchman JJ. 2007. Pair of lice lost or parasites regained: the evolutionary history of anthropoid primate lice. BMC Biol. 5:7. 17343749 10.1186/1741-7007-5-7 PMC1828715

[msab351-B110] Reed DL , Smith VS, Hammond SL, Rogers AR, Clayton DH. 2004. Genetic analysis of lice supports direct contact between modern and archaic humans. PLoS Biol. 2(11):e340. 15502871 10.1371/journal.pbio.0020340 PMC521174

[msab351-B111] Reimer PJ , Austin WEN, Bard E, Bayliss A, Blackwell PG, Bronk Ramsey C, Butzin M, Cheng H, Edwards RL, Friedrich M, et al 2020. The IntCal20 northern hemisphere radiocarbon age calibration curve (0–55 cal kBP). Radiocarbon 62(4):725–757.

[msab351-B112] Reinhard KJ , Buikstra J. 2003. Louse infestation of the Chiribaya culture, Southern Peru: variation in prevalence by age and sex. Mem Inst Oswaldo Cruz. 98(Suppl 1):173–179. 12687779 10.1590/s0074-02762003000900026

[msab351-B113] Rohland N , Glocke I, Aximu-Petri A, Meyer M. 2018. Extraction of highly degraded DNA from ancient bones, teeth and sediments for high-throughput sequencing. Nat Protoc. 13(11):2447–2461. 30323185 10.1038/s41596-018-0050-5

[msab351-B114] Russo MG , Gheggi MS, Avena SA, Dejean CB, Cremonte MB. 2017. Mitochondrial lineages in samples of Esquina de la Huajra (Jujuy, Argentina). Contributions to the study of the Inka occupation in the region and to the origin of its inhabitants. Rev Argent Antropol Biol. 19(1):1–15.

[msab351-B115] Sakalauskaitė-Juodeikienė E , Jatužis D, Kaubrys S. 2018. Plica polonica: from national plague to death of the disease in the nineteenth-century Vilnius. Indian J Dermatol Venereol Leprol. 84(4):510–514. 29798937 10.4103/ijdvl.IJDVL_673_17

[msab351-B116] Sawchuk E , Prendergast ME. 2019. Ancient DNA is a powerful tool for studying the past – when archaeologists and geneticists work together. The Conversation 2019:111127.

[msab351-B117] Scheib CL , Li H, Desai T, Link V, Kendall C, Dewar G, Griffith PW, Mörseburg A, Johnson JR, Potter A, et al 2018. Ancient human parallel lineages within North America contributed to a coastal expansion. Science 360(6392):1024–1027. 29853687 10.1126/science.aar6851

[msab351-B118] Schubert M , Lindgreen S, Orlando L. 2016. AdapterRemoval v2: rapid adapter trimming, identification, and read merging. BMC Res Notes. 9:88. 26868221 10.1186/s13104-016-1900-2 PMC4751634

[msab351-B119] Shapiro B , Hofreiter M. 2012. Ancient DNA. Methods and protocols. New York: Humana Press.

[msab351-B120] Silva Noelli F. 2008. The Tupi expansion. In: Silverman H, WIsbell WH, editors. The handbook of South American archaeology. New York: Springer. p. 659–670.

[msab351-B121] Simonson TS , Xing J, Barrett R, Jerah E, Loa P, Zhang Y, Watkins WS, Witherspoon DJ, Huff CD, Woodward S, et al 2011. Ancestry of the Iban is predominantly Southeast Asian: genetic evidence from autosomal, mitochondrial, and Y chromosomes. PLoS One 6(1):e16338. 21305013 10.1371/journal.pone.0016338 PMC3031551

[msab351-B122] Sirak K , Fernandes D, Cheronet O, Harney E, Mah M, Mallick S, Rohland N, Adamski N, Broomandkhoshbacht N, Callan K, et al 2020. Human auditory ossicles as an alternative optimal source of ancient DNA. Genome Res. 30(3):427–436. 32098773 10.1101/gr.260141.119 PMC7111520

[msab351-B123] Sirak KA , Fernandes DM, Cheronet O, Novak M, Gamarra B, Balassa T, Bernert Z, Cseki A, Dani J, Gallina JZ, et al 2017. A minimally-invasive method for sampling human petrous bones from the cranial base for ancient DNA analysis. Biotechniques 62(6):283–289. 28625158 10.2144/000114558

[msab351-B124] Skoglund P , Storå J, Götherström A, Jakobsson M. 2013. Accurate sex identification of ancient human remains using DNA shotgun sequencing. J Archaeol Sci. 40(12):4477–4482.

[msab351-B125] Slon V , Hopfe C, Weiß CL, Mafessoni F, de la Rasilla M, Lalueza-Fox C, Rosas A, Soressi M, Knul MV, Miller R, et al 2017. Neandertal and Denisovan DNA from Pleistocene sediments. Science 356(6338):605–608. 28450384 10.1126/science.aam9695

[msab351-B126] Taboada-Echalar P , Alvarez-Iglesias V, Heinz T, Vidal-Bralo L, Gomez-Carballa A, Catelli L, Pardo-Seco J, Pastoriza A, Carracedo A, Torres-Balanza A, et al 2013. The genetic legacy of the pre-colonial period in contemporary Bolivians. PLoS One 8(3):e58980. 23527064 10.1371/journal.pone.0058980 PMC3604014

[msab351-B127] Tridico SR , Rigby P, Kirkbride KP, Haile J, Bunce M. 2014. Megafaunal split ends: microscopical characterisation of hair structure and function in extinct woolly mammoth and woolly rhino. Quatern Sci Rev. 83:68–75.

[msab351-B128] van der Meijden E , Bialasiewicz S, Rockett RJ, Tozer SJ, Sloots TP, Feltkamp MC. 2013. Different serologic behavior of MCPyV, TSPyV, HPyV6, HPyV7 and HPyV9 polyomaviruses found on the skin. PLoS One 8(11):e81078. 24278381 10.1371/journal.pone.0081078 PMC3836759

[msab351-B129] Vidal EA , Moyano TC, Bustos BI, Perez-Palma E, Moraga C, Riveras E, Montecinos A, Azocar L, Soto DC, Vidal M, et al 2019. Whole genome sequence, variant discovery and annotation in Mapuche-Huilliche Native South Americans. Sci Rep. 9(1):2132. 30765821 10.1038/s41598-019-39391-z PMC6376018

[msab351-B130] Wagner JK , Colwell C, Claw KG, Stone AC, Bolnick DA, Hawks J, Brothers KB, Garrison NA. 2020. Fostering responsible research on ancient DNA. Am J Hum Genet. 107(2):183–195. 32763189 10.1016/j.ajhg.2020.06.017 PMC7413888

[msab351-B131] Warinner C , Rodrigues JF, Vyas R, Trachsel C, Shved N, Grossmann J, Radini A, Hancock Y, Tito RY, Fiddyment S, et al 2014. Pathogens and host immunity in the ancient human oral cavity. Nat Genet. 46(4):336–344. 24562188 10.1038/ng.2906 PMC3969750

[msab351-B132] Wen T , Zhaoyong X, Zhijie G, Yeshua X, Jianghua S, Zhiyi G. 1987. Observation on the ancient lice from Loulan. Invest Stud NatMus Hist Nat Shangh. 7:152–155.

[msab351-B133] Wood J. 2018. DNA barcoding of ancient parasites. Parasitology 145(5):646–655. 29557324 10.1017/S0031182018000380

[msab351-B134] Woods R , Barnes I, Brace S, Turvey ST. 2021. Ancient DNA suggests single colonization and within-archipelago diversification of Caribbean caviomorph rodents. Mol Biol Evol. 38(1):84–95. 33035304 10.1093/molbev/msaa189 PMC7783164

[msab351-B135] Zhang D , Xia H, Chen F, Li B, Slon V, Cheng T, Yang R, Jacobs Z, Dai Q, Massilani D, et al 2020. Denisovan DNA in Late Pleistocene sediments from Baishiya Karst Cave on the Tibetan Plateau. Science 370(6516):584–587. 33122381 10.1126/science.abb6320

[msab351-B136] Zias J , Mumcuoglu KY. 1991. Pre-pottery Neolithic B head lice from Nahal Hemar cave. Atiqot 20:167–168.

