# Supplementary material for: Ancient Human Genomes and Environmental DNA from the Cement Attaching 2,000-Year-Old Head Lice Nits
Source: Mol Biol Evol. 2021 Dec 28;39(2):msab351. doi: 10.1093/molbev/msab351 (PMC8829908; doi:10.1093/molbev/msab351)
Supplement: msab351_Supplementary_Data [file msab351_supplementary_data.zip › Answers EditorReviewers R3 MS- MBE-21-0357.R2.docx]

**Answers to Reviewer and Editor, Review3, MBE-21-0357.R2**

Please find our answers below each of the comments

**Editor**

**1)**The comments and recommendations from one expert reviewers are now available for your manuscript. The reviewer is pleased with the improvements and considers the science to be solid, but recommends a thorough proofreading of the piece to avoid mistakes and difficult-to-read sentences. 

Editors generally agree with their concerns and recommendations, which led to a designation of high priority. The recommendation is given assuming that you will be able to satisfactorily address all the reviewers' comments (including my own, as given below). Without a satisfactory response, the priority may decline in the next round of review/consideration. 

I still find the PCA difficult to interpret with outlines in the symbols. A good combination of symbol shapes and colors (without symbol outlines) will make it way easier to interpret. Please change this in the new version.

*Answer*

We followed the recommendation of the Editor and made a new Figure 4 (PCA) with a good combination of symbol shapes and colors (without symbol outlines). We also arranged the legend of the 2^nd^ panel accordingly, for more clarity.

The last uploaded versions of the manuscript include a thorough review of the text by the English native co-authors (e.g. Stuart Black), considering the request of proof-reading the article and all the comments of Reviewer 1.

**Reviewers' comments: 
Reviewer: 1**
Comments to the Author 
**2)** In terms of the science, the authors have satisfactorily addressed my comments on the previous version. I recommend the article for publication. 

However, despite two rounds of revision, there remain poorly worded sentences and instances of incorrect use of punctuation marks in the manuscript. I recommend another thorough proof-reading of the article, since I am sure I did not manage to catch all such instances (see below). Specifically, I request the authors to pay attention to their use of commas in place of semi-colons, and vice-versa. Also, the authors should scan the manuscript for sentences beginning with unnecessary instances of words such as “while”.

*Answer*

We apologise for this and hope that the latest thorough revision of the text by the two English native co-authors (from Oxford and Reading Universities), who proof-read it is now satisfactory. All corrections, rewording and changes are found in the ‘marked’ file in red color.

**3)** Line 91: Reword - Nits are individually “attached”.

*Answer:* corrected as requested by the Reviewer

**4)** Lines 98-99: Reword – “we hypothesized that nit cement is able to trap and preserve host DNA.”

*Answer:* reworded as requested by the Reviewer

**5)** Line 112: It is unclear what OUMNH stands for.

*Answer:* reworded: … Anoplura collection from the Oxford University Museum of Natural History (OUMNH)…

**6)** Line 199: Remove the word “while”. The sentence should read “We found a mean coverage on the mitochondrial genome of …”

*Answer:* corrected

**7)** Lines 253-255: Change these sentences to read as follows - “We next sought to determine Y chromosomal haplogroup. While the coverage for Dyak-Louse and SJArg-1-Nit were found to be too low for calling the haplotypes, SJArg-2-Nit (Y-coverage 0.038x) was characterised as Q1b1a1a1 or Q-M3 (M848)…”

*Answer:* sentences changed

**8)** Line 330: Reword – “All our South American individuals were found to belong to mitochondrial haplogroup A2, consistent with previous studies…”

*Answer:* sentence reworded
