# Supplementary material for: Ancient Human Genomes and Environmental DNA from the Cement Attaching 2,000-Year-Old Head Lice Nits
Source: Mol Biol Evol. 2021 Dec 28;39(2):msab351. doi: 10.1093/molbev/msab351 (PMC8829908; doi:10.1093/molbev/msab351)
Supplement: msab351_Supplementary_Data [file msab351_supplementary_data.zip › Supplementary Material aHost genomes revised S.pdf]

Supplementary Material

Ancient human genomes and environmental DNA from the cement attaching 2,000 year-old head lice nits

Mikkel W. Pedersen[1*], Catia Antunes[2#], Binia De Cahsan[1#], J. Víctor Moreno-Mayar[1], Martin Sikora[1], Lasse Vinner[1], Darren Mann[3], Pavel B. Klimov[4,5], Stuart Black[6], Catalina Teresa Michieli[7], Henk R. Braig[4,8#] and M. Alejandra Perotti[2#*]

[1] GLOBE Institute, Faculty of Health and Medical Science, University of Copenhagen, Denmark.

[2] Ecology and Evolutionary Biology Section, School of Biological Sciences, University of Reading, Reading, United Kingdom.

[3] Oxford University Museum of Natural History, Oxford, United Kingdom.

[4] School of Natural Sciences, Bangor University, Bangor, Wales, United Kingdom.

[5] Department of Ecology and Evolutionary Biology, University of Michigan, Museum of Zoology, Ann Arbor, 48103, USA.

[6] Department of Geography and Environmental Science, Wager Building, University of Reading, Reading, United Kingdom.

[7] Instituto de Investigaciones Arqueológicas y Museo "Prof. Mariano Gambier", Universidad Nacional de San Juan, San Juan, Argentina.

[8] Institute and Museum of Natural Sciences, Faculty of Exact, Physical and Natural Sciences, National University of San Juan, San Juan, J5400DNQ, Argentina

*Corresponding authors

M. Alejandra Perotti, m.a.perotti@reading.ac.uk

Mikkel Winther Pedersen mwpedersen@sund.ku.dk

CONTENTS

Information of samples and permits granted 3

Sheaths/cement measurements, quality of hair 3

SUPPLEMENTARY FIGURES

Figure S1. Quality of hair scales 4
Figure S2. Location of SJArg-1-Tooth 5
Figure S3. Contamination estimates for SJArg-1-Tooth 6
Figure S4. Contamination estimates for SJArg-3-Petrous 7
Figure S5. Contamination estimates for SJArg-1-Nit 8
Figure S6. Contamination estimates for SJArg-2-Nit 9
Figure S7. Contamination estimates for Dyak-Louse 10
Figure S8. Human chromosome sex determination 11
Figure S9. Principal component analysis 12
Figure S10. Relative proportion of reads taxonomically classified… 13
Figure S11. Damage/fragmentation estimates. Dyak-Louse/whole human genome 14
Figure S12. Damage/fragmentation estimates. Dyak-Louse/mt human genome 15
Figure S13. Damage/fragmentation estimates. Dyak-Louse/Pediculus scaffold 16
Figure S14. Damage/fragmentation estimates. SJArg-1-Nit/whole human genome 17
Figure S15. Damage/fragmentation estimates. SJArg-1-Nit/ mt human genome 18
Figure S16. Damage/fragmentation estimates. SJArg-1-Nit/ Pediculus scaffold 19
Figure S17. Damage/fragmentation estimates. SJArg-2-Nit/whole human genome 20
Figure S18. Damage/fragmentation estimates. SJArg-2-Nit/ mt human genome 21
Figure S19. Damage/fragmentation estimates. SJArg-2-Nit/ Pediculus scaffold 22
Figure S20. Damage/fragmentation estimates. SJArg-1-Tooth/whole human genome 23
Figure S21. Damage/fragmentation estimates. SJArg-1-Tooth/mt human genome 24
Figure S22. Damage/fragmentation estimates. SJArg-3-Petrous/whole human genome 25
Figure S23. Damage/fragmentation estimates. SJArg-3-Petrous/mt human genome 26
Figure S24. Distribution of reads assigned by Holi to Merkel cell polyomavirus 26

SUPPLEMENTARY TABLES

Table S1. Radiocarbon dating (C^{14}) 27
Table S2. List of ancient human specimens 28
Table S3. Length of nit cement 29
Table S4. Summary statistics of length of cement by age 30
Table S5. Distance of nits from root 31
Table S6. Summary statistics of distance from scalp (mm) by mummy 32
Table S7. Genetic metadata 33
Table S8. Y Chromosomal haplogroup 34

REFERENCES 35

Information of samples and permits granted.

Permits/Certificates for sampling, transport, destructive sampling and genetic analyses Work using the tooth and bone specimens of fragmented skeletons SJ-Arg-1-Tooth and SJ-Arg-3-Petrous, and nits (SJArg-2-Nit and SJArg-4 to 7 Nit) from Argentinian specimens follow a collaboration agreement between Dra. Catalina Teresa Michieli, Director of Instituto de Investigaciones Arqueológicas y Museo "Prof. Mariano Gambier" UNSJ and Dr. M. Alejandra Perotti and the following permits were issued:

A-Permit No 0354 October 5 2017, for studies of cultural heritage, issued by Ministerio de Cultura de la Provincia de San Juan, Argentina.
B-Certificate of registration No 1200-1823-17 (2017), by the Ministerio de Turismo y Cultura, Dirección de Patrimonio Cultural y Natural.
C-Certificates for transport of specimens within Argentina and abroad were also obtained: Transit Permit No 0431 (2017) and Approval Transfer No R056 (2017) of the Secretaría de Ambiente y desarrollo Sustentable, Subsecretaria de Conservación y Areas Protegidas, Direccion de Areas Protegidas, Gobierno de la Provincia de San Juan.
D-Work on Chilean nits from specimens SJ-Chi-8-Nit and SJ-Chi-9-Nit follows approval by Dr. Pilar Alliende, Manager of Collections, Museo Chileno de Arte Precolombino-March-2017.
E-Approval for destructive sampling of Dayak lice from the Denny collection was requested in 2014 and granted in 2017 by the Head of Collections, Oxford University Museum of Natural History in accordance with the OUMNH Destructive Sampling Policy-2016.

Information on radiocarbon dating of specimens is given in table S1. Information of each samples/specimen is listed in table S2.

Sheaths/cement measurements, quality of hair

Measurements of length of cement and quality of hair scales were collected from nit sheaths of eight mummies of different ages provided by the Argentinian and Chilean museums, spanning ~1800 years, from approx. 300 BP to 2100 BP (Table S3). The analysis included mummies SJ-Arg-1-Nit and SJ-Arg-2-Nit, which are part of the genetic studies. Measurements were done using Zen 3.1. (Karl Zeiss) on live and captured images of scanned sets (in 2D), taken at 200X and 400X magnification, in an inverted confocal microscope, Zeiss LSM 710.
For information of the environmental conditions at the time before death, the measures of shaft distance from nit to root were collected from three mummies SJArg-1, SJArg-2 and SJArg-4, as the colder the temperature the closer to the scalp the louse lays its eggs (Buxton 1940; Leeson 1941b, a; Busvine 1948; Maunder 1983). The distribution of nits on hair, especially the distance from scalp, was studied on three ancient hosts during sampling, SJ-Arg-1-Nit (young adult), SJ-Arg-2-Nit (young adult) and SJ-Arg-4-Nit (mature/elder). Most nits contained dead louse larvae (approx. 70%), and were located closer than expected to the scalp. The hair roots were supported by very dry, dusted skin, at an unusual minimum distance of 2 mm (Table S4). These measures were collected when sampling *in situ*, at the Gambier Institute.
Data collected were i)distance of nits from scalp and ii)length of cement cover, per mummy or per age. Summary statistics and results of analyses of both sets are included in Tables S3 to S6. Normality tests were performed to assess data distribution within each and also for all mummies; ANOVA applied to cement length by age groups, and post-hoc analyses compared individual ages and mummies; for linear correlation (bivariate) of length of cement vs age of mummy, Pearson coefficient 'r' was applied (Hammer et al. 2001).

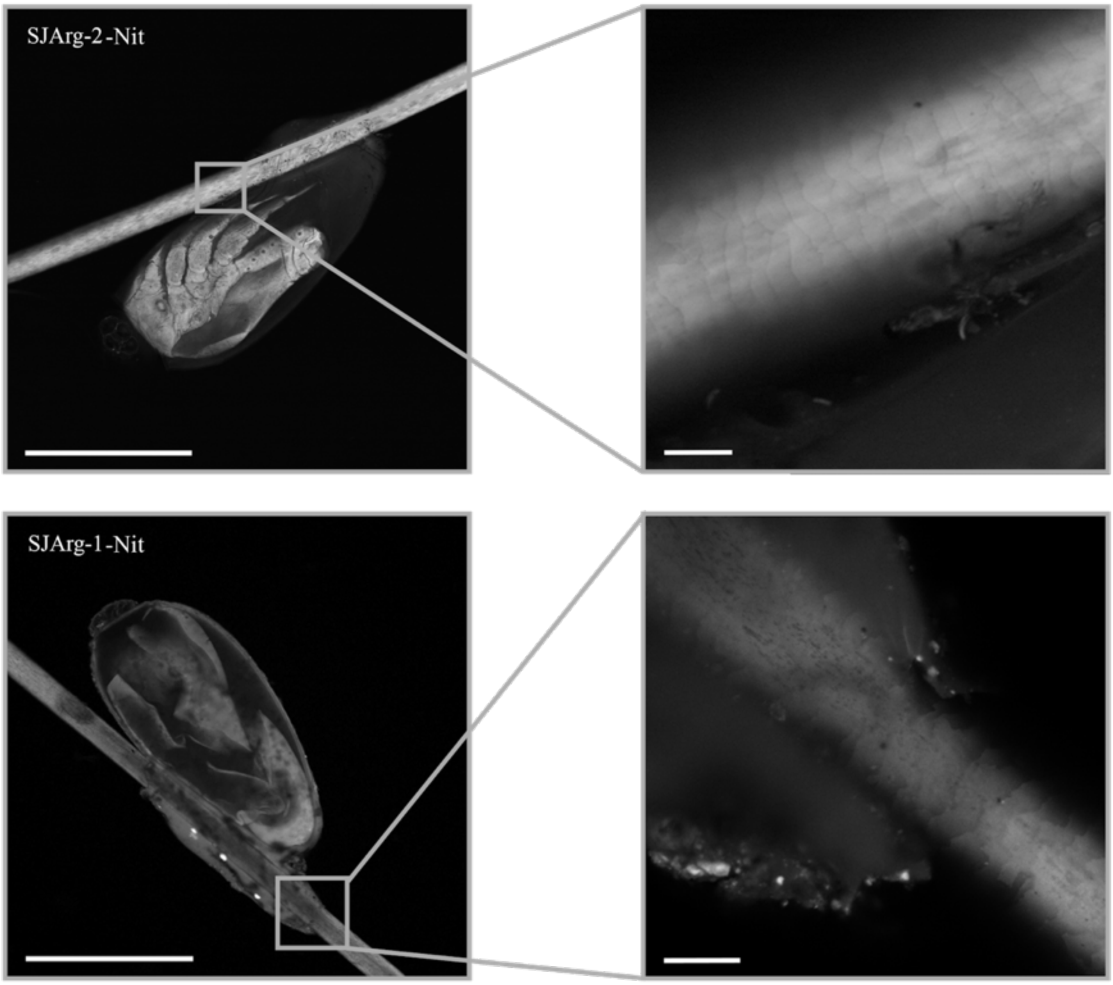

Figure S1. Quality of hair scales. Sheath samples of the two mummy specimens used in the whole, wide genome analyses showing the preservation of the hair scales, fluorescence confocal microscopy. Magnified sections of each sheath on the right hand side, with the detail of the shape of scales. Specimen SJArg-1-Nit shows weak preservation with broken and uneven distribution of scales, while SJArg-2-Nit, despite being older by ~500 years, is in better shape. Note some nuclei trapped in the cement tube of SJArg-1-Nit. Bars 500 and 25 µm.

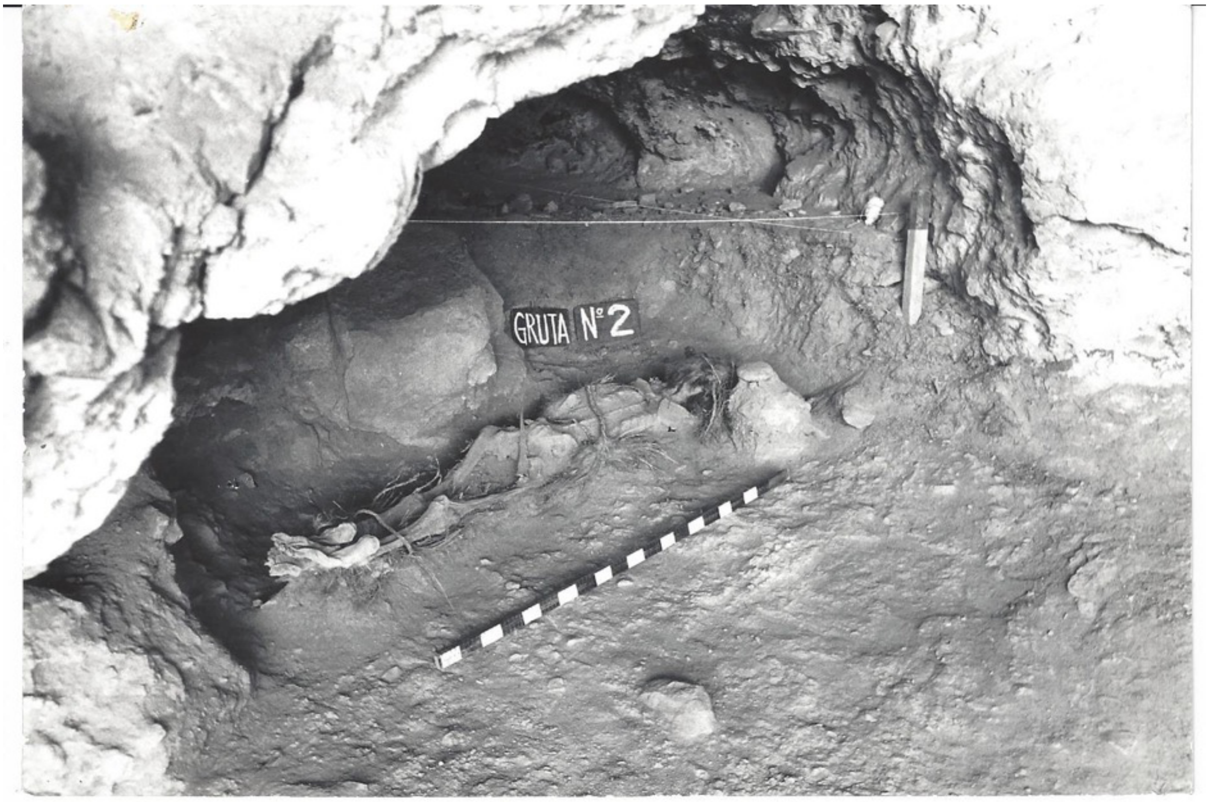

Figure S2. Location of SJArg-1-Tooth at the atrium, terrace of the cave. Original photograph provided by the Instituto de Investigaciones Arqueológicas y Museo “Prof. Mariano Gambier” UNSJ.

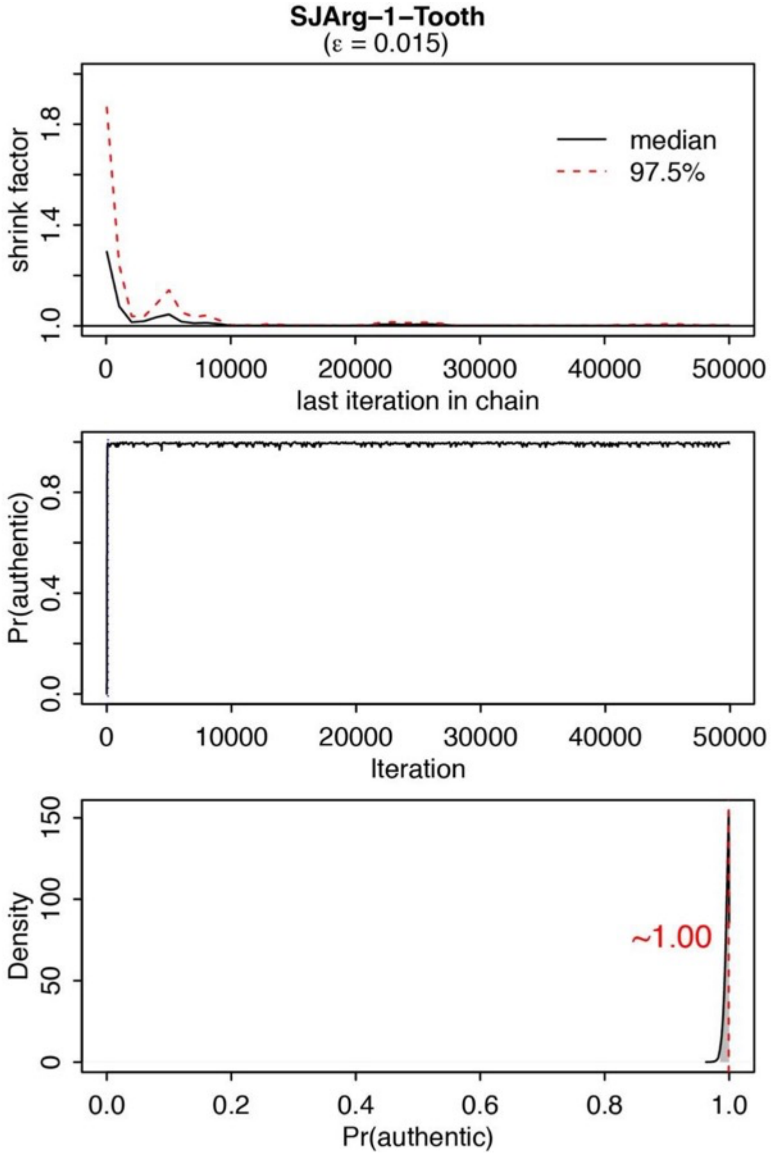

Figure S3. Mitochondrial contamination estimation diagnostics for the SJArg-1-Tooth sample. We used contamMix to estimate the amount of human mtDNA contamination for each sample. For each point estimate reported in the text, we assess the convergence of the MCMC estimation by plotting the Gelman-Rubin diagnostic (top panel) and the estimated posterior authentic proportion (middle panel) after each MCMC iteration. In the bottom panel, we show the estimated posterior density for the authentic proportion, together with the point estimate.

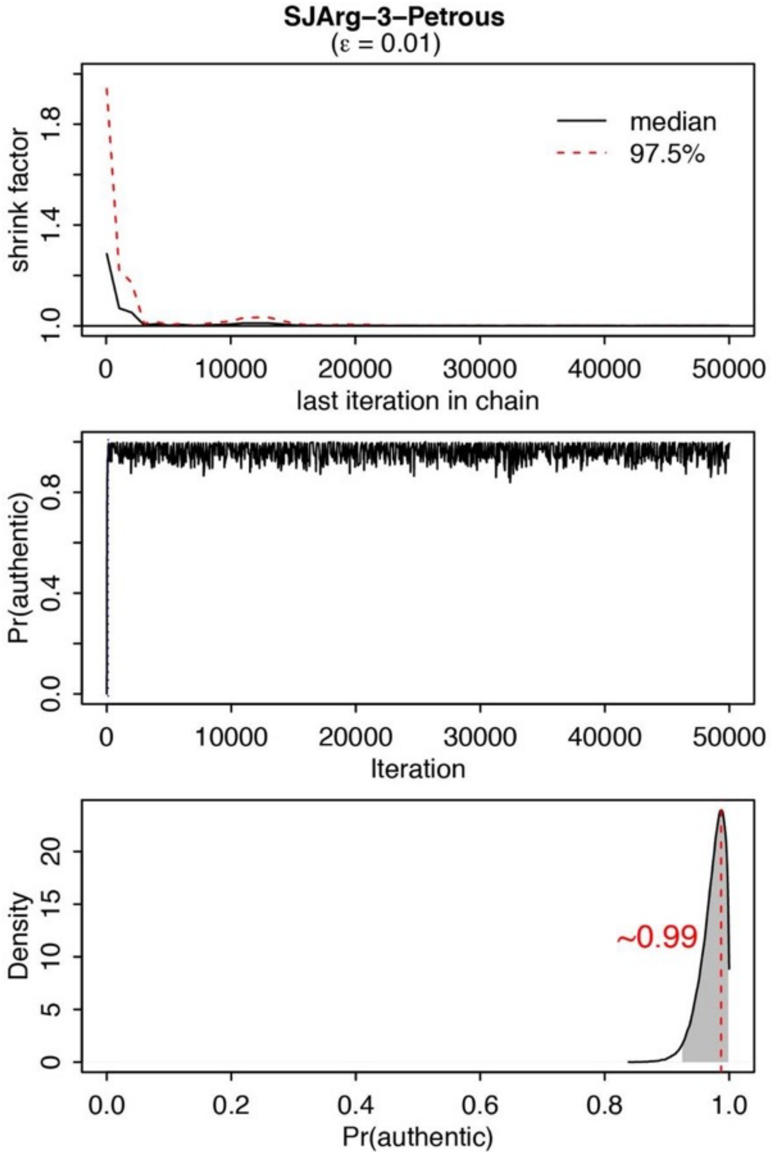

Figure S4. Mitochondrial contamination estimation diagnostics for the SJArg-3-Petrous sample. We used contamMix to estimate the amount of human mtDNA contamination for each sample. For each point estimate reported in the text, we assess the convergence of the MCMC estimation by plotting the Gelman-Rubin diagnostic (top panel) and the estimated posterior authentic proportion (middle panel) after each MCMC iteration. In the bottom panel, we show the estimated posterior density for the authentic proportion, together with the point estimate.

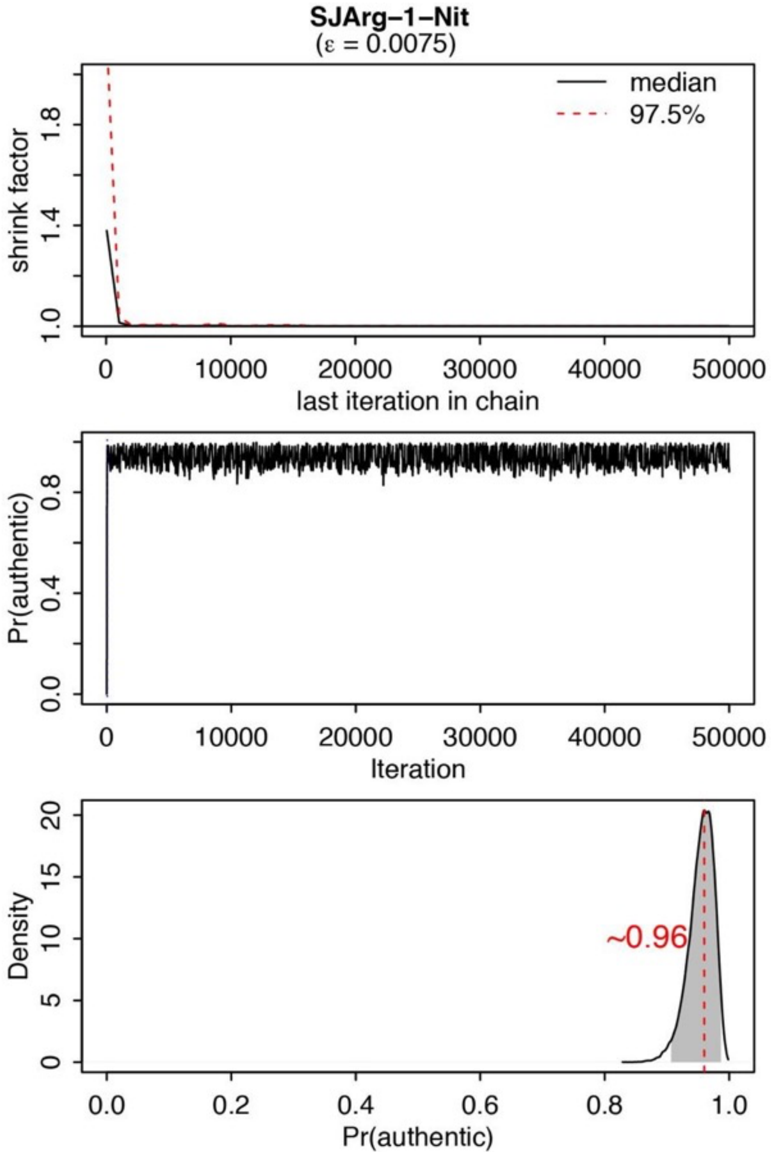

Figure S5. Mitochondrial contamination estimation diagnostics for the SJArg-1-Nit sample. We used contamMix to estimate the amount of human mtDNA contamination for each sample. For each point estimate reported in the text, we assess the convergence of the MCMC estimation by plotting the Gelman-Rubin diagnostic (top panel) and the estimated posterior authentic proportion (middle panel) after each MCMC iteration. In the bottom panel, we show the estimated posterior density for the authentic proportion, together with the point estimate.

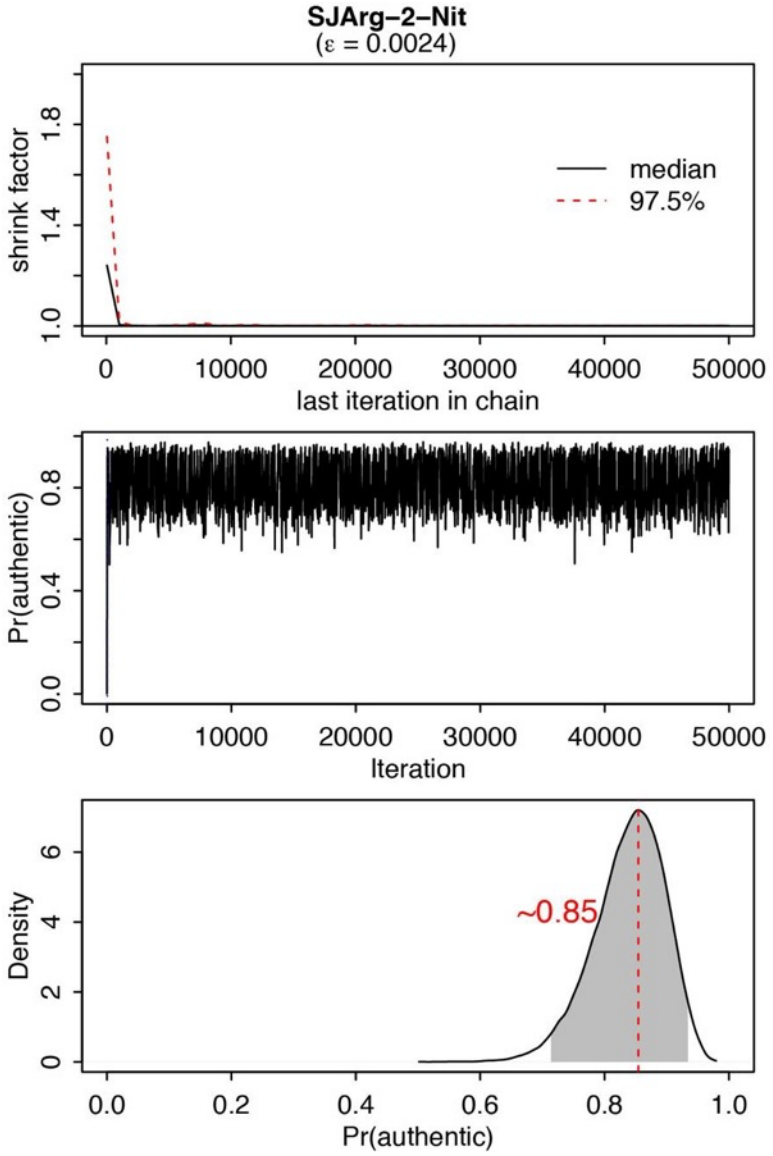

Figure S6. Mitochondrial contamination estimation diagnostics for the SJArg-2-Nit sample. We used contamMix to estimate the amount of human mtDNA contamination for each sample. For each point estimate reported in the text, we assess the convergence of the MCMC estimation by plotting the Gelman-Rubin diagnostic (top panel) and the estimated posterior authentic proportion (middle panel) after each MCMC iteration. In the bottom panel, we show the estimated posterior density for the authentic proportion, together with the point estimate.

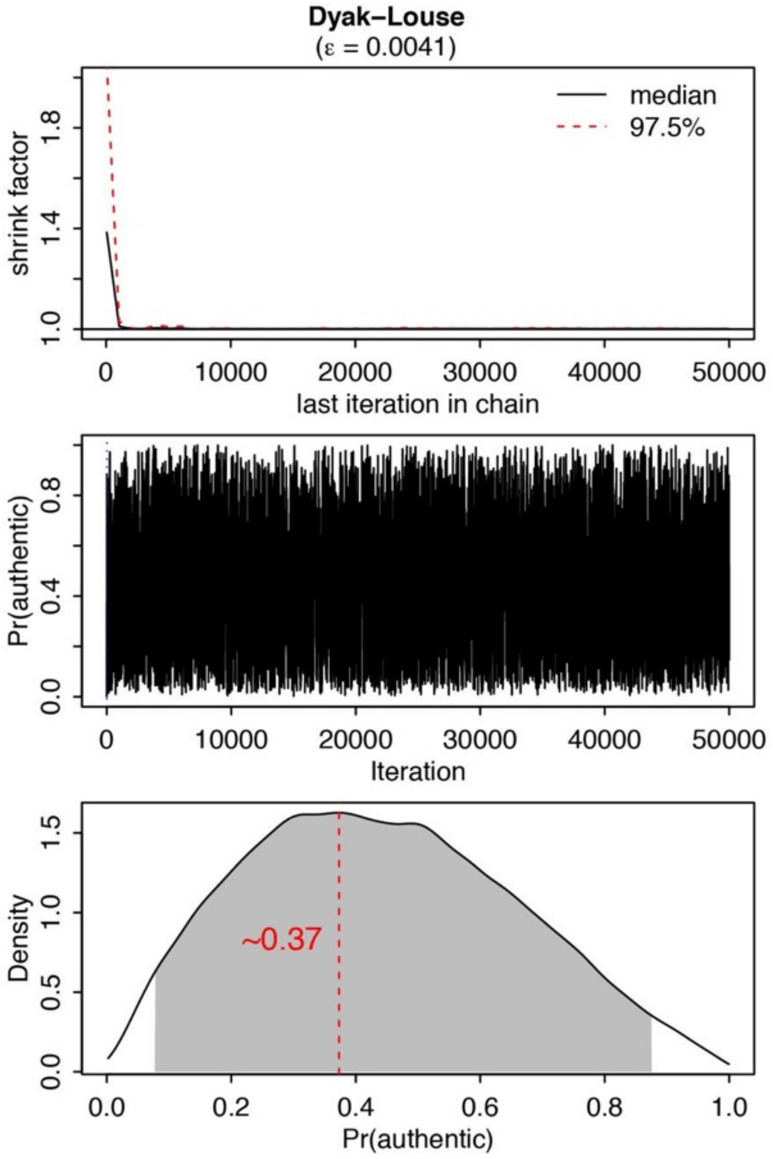

Figure S7. Mitochondrial contamination estimation diagnostics for the Dyak-Louse. We used contamMix to estimate the amount of human mtDNA contamination for each sample. For each point estimate reported in the text, we assess the convergence of the MCMC estimation by plotting the Gelman-Rubin diagnostic (top panel) and the estimated posterior authentic proportion (middle panel) after each MCMC iteration. In the bottom panel, we show the estimated posterior density for the authentic proportion, together with the point estimate.

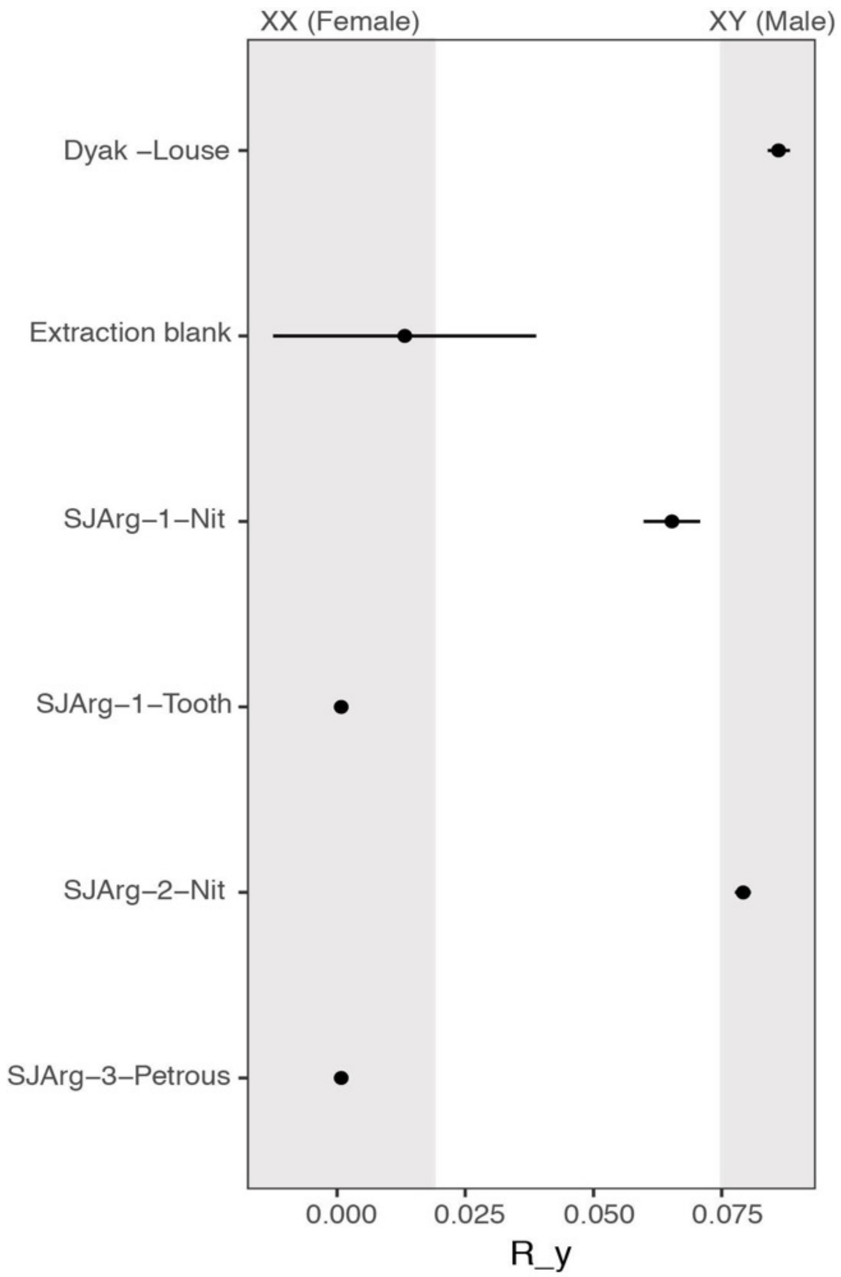

Figure S8. Human chromosome sex determination. Observed fraction of Y chromosome alignments compared to the total number of alignments to the X and Y chromosome. The fraction sequences aligned to the Y-chromosome is expressed as a ratio of the total number of sequences aligned to either sex chromosome (R_Y). Error bars represent the 95% confidence intervals.

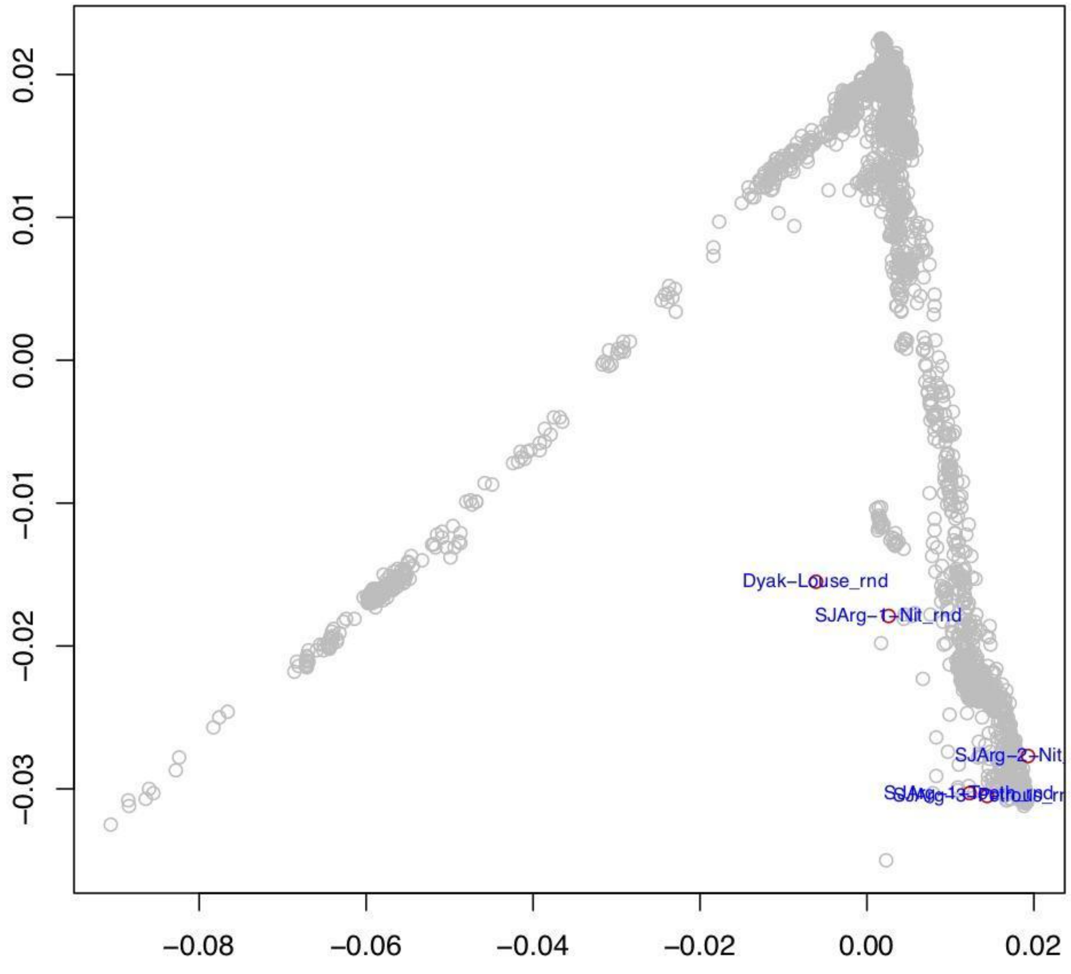

Figure S9. Principal components analysis (PCA) with the ancient low-coverage samples projected onto a genotyped reference dataset (REF).

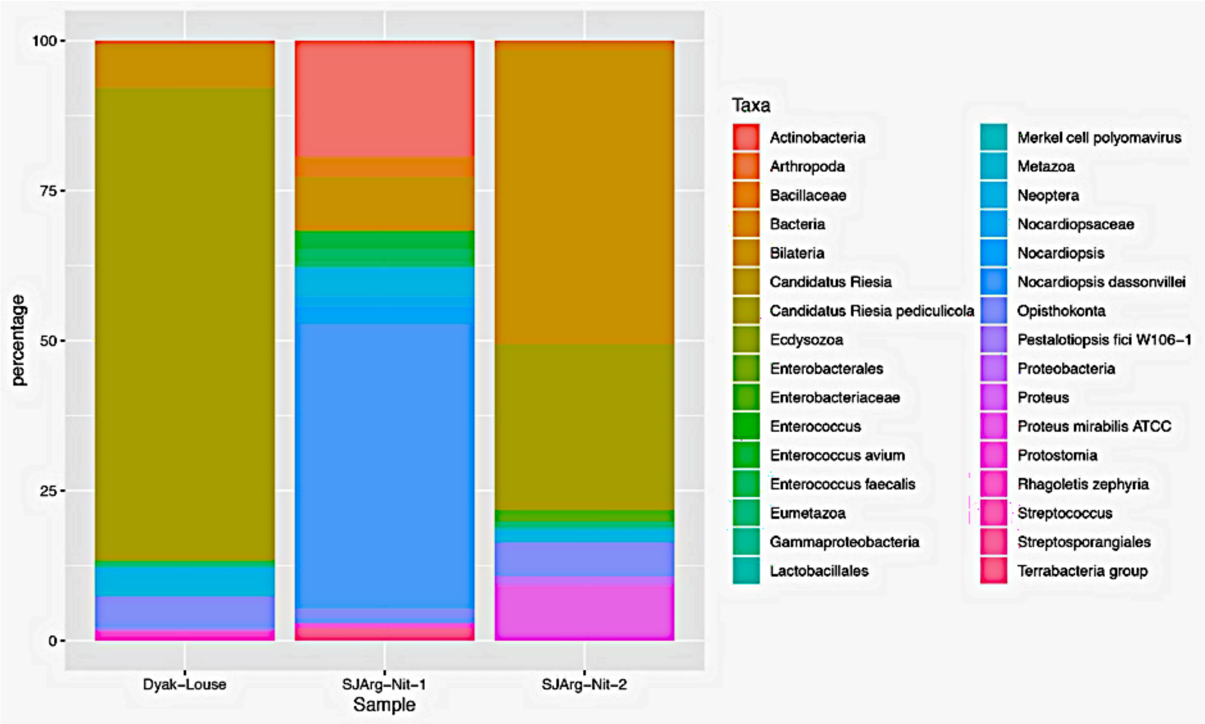

Figure S10. Relative proportion of reads taxonomically classified to other organisms than human and louse. All reads are presented at the taxonomic level classified, filtering for taxa < 50 reads and with DNA damage less than for the human and louse.

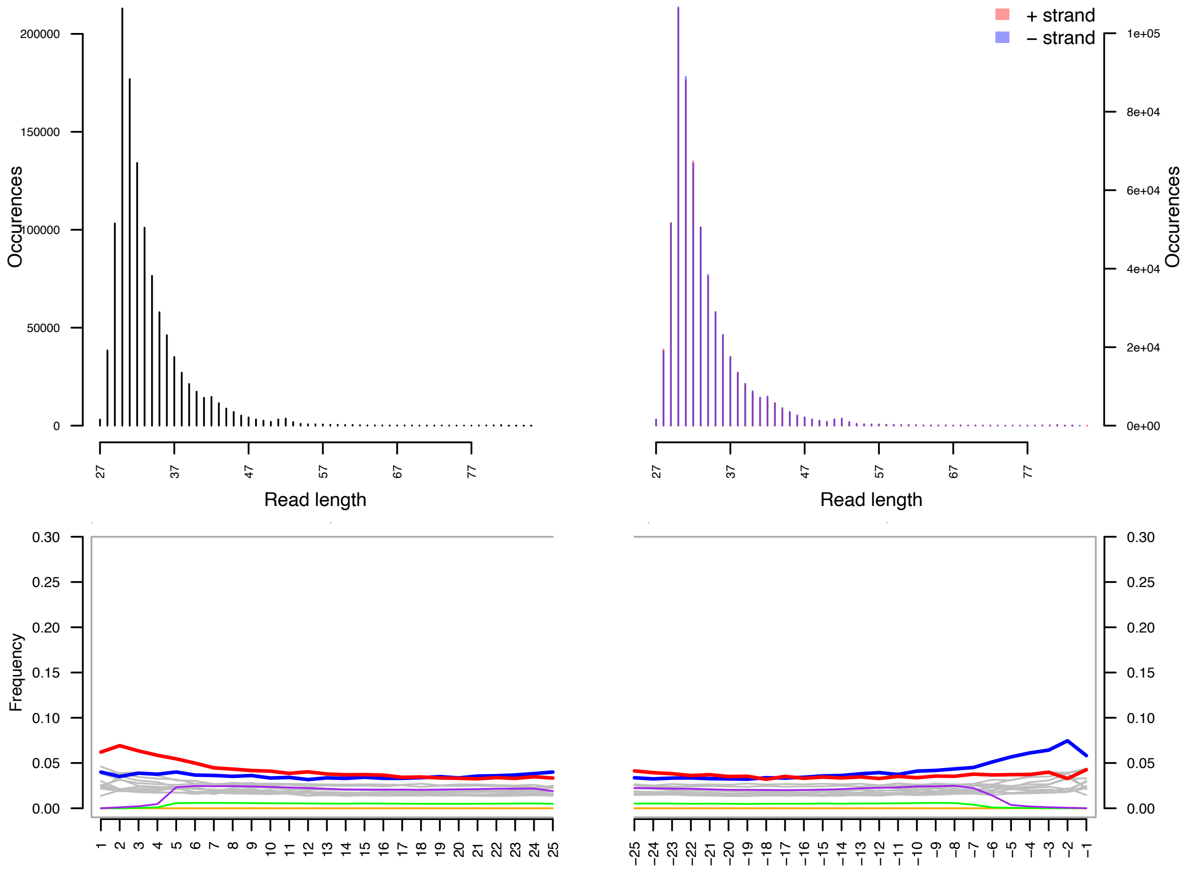

Figure S11. Damage and fragmentation estimates for reads aligned to the whole human genome (Hs37d5) for the Dyak-Louse. Single-end length distribution (top left panel) and per strand (top right panel). The bottom two plots are the positions' specific substitutions from the 5" (left) and the 3" end (right). The following color codes are used in the bottom plots: **Red**: C to T substitutions. **Blue**: G to A substitutions. **Grey**: All other substitutions. **Orange**: Soft-clipped bases. **Green**: Deletions relative to the reference. **Purple**: Insertions relative to the reference.

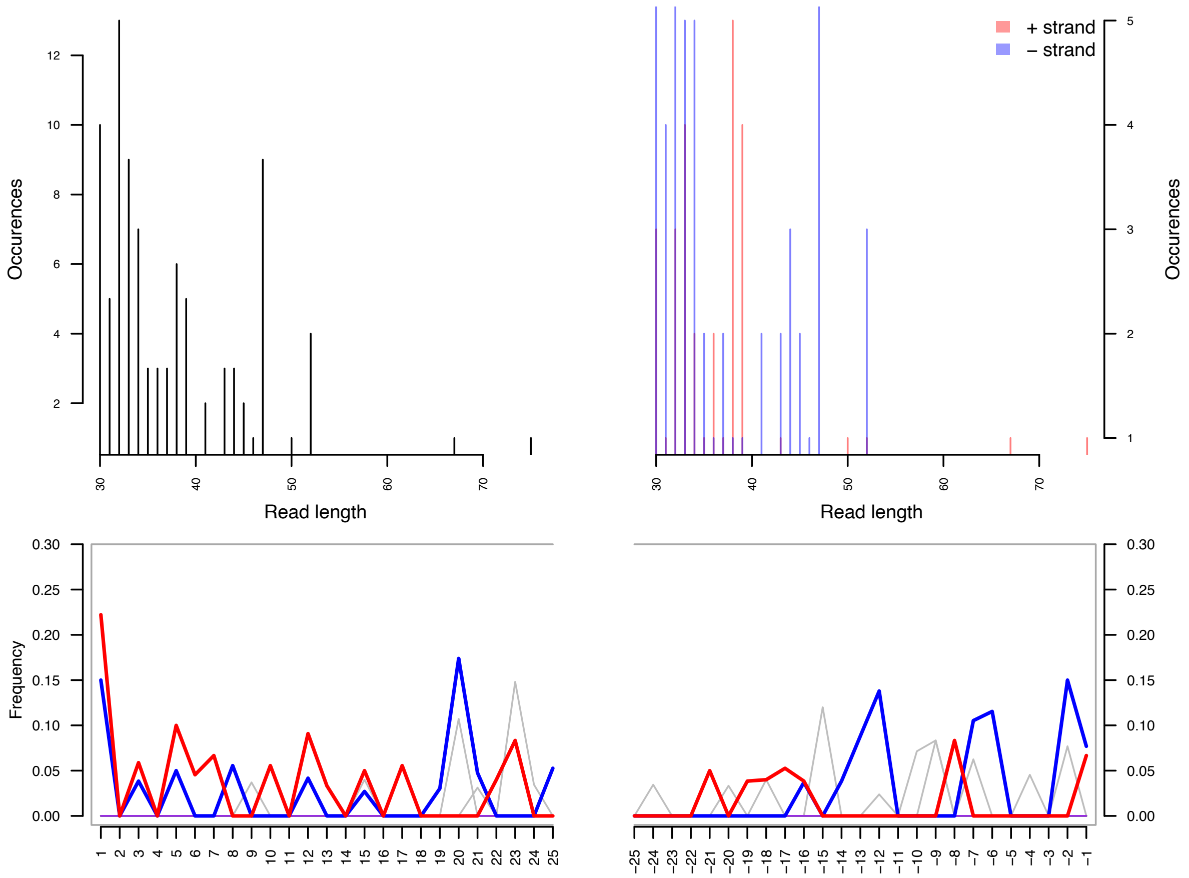

Figure S12. Damage and fragmentation estimates for reads aligned to the mitochondrial genome (rCRS) for the Dyak-Louse. The plots are very noisy due to too low number of reads. Single-end length distribution (top left panel) and per strand (top right panel). The bottom two plots are the positions' specific substitutions from the 5" (left) and the 3" end (right). The following color codes are used in the bottom plots: **Red**: C to T substitutions. **Blue**: G to A substitutions. **Grey**: All other substitutions. **Orange**: Soft-clipped bases. **Green**: Deletions relative to the reference. **Purple**: Insertions relative to the reference.

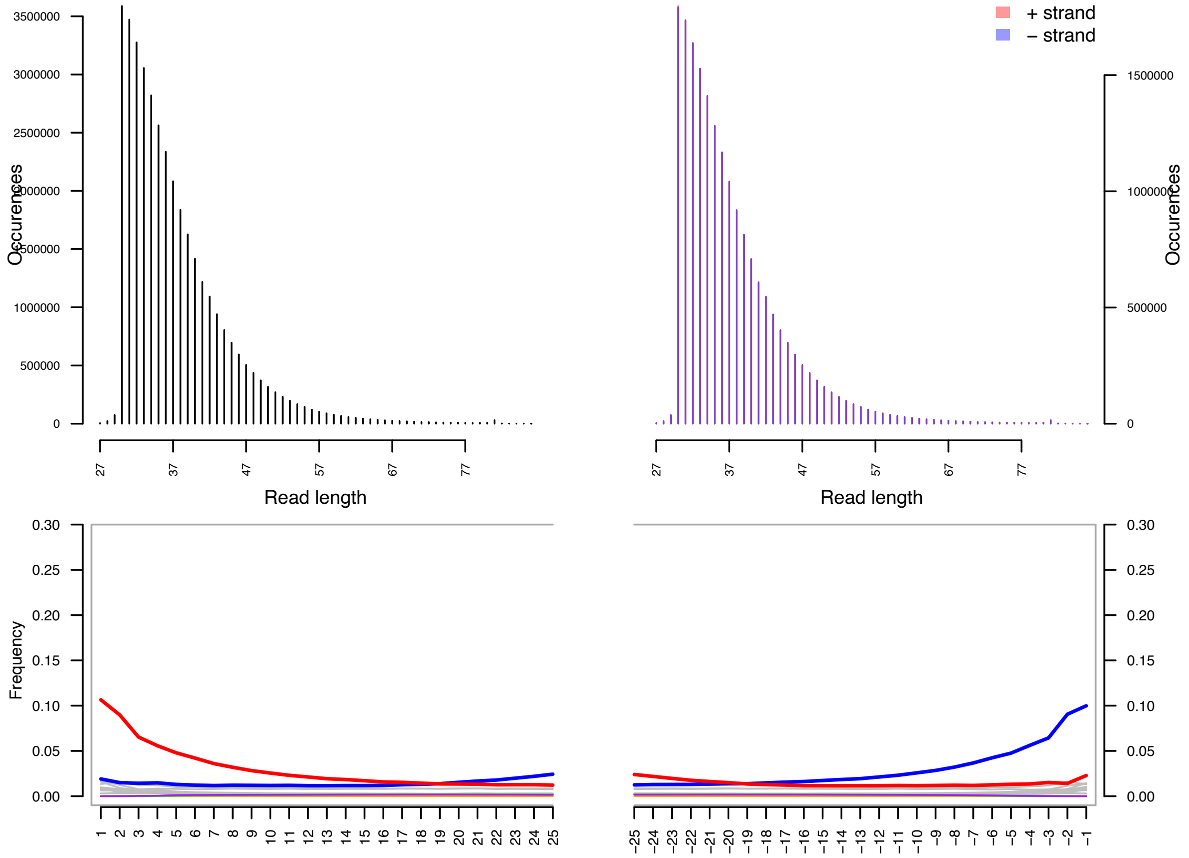

Figure S13. Damage and fragmentation estimates for reads aligned to the *Pediculus humanus corporis* scaffold (PRJNA16223) for the Dyak-Louse. Single-end length distribution (top left panel) and per strand (top right panel). The bottom two plots are the positions' specific substitutions from the 5" (left) and the 3" end (right). The following color codes are used in the bottom plots: **Red**: C to T substitutions. **Blue**: G to A substitutions. **Grey**: All other substitutions. **Orange**: Soft-clipped bases. **Green**: Deletions relative to the reference. **Purple**: Insertions relative to the reference.

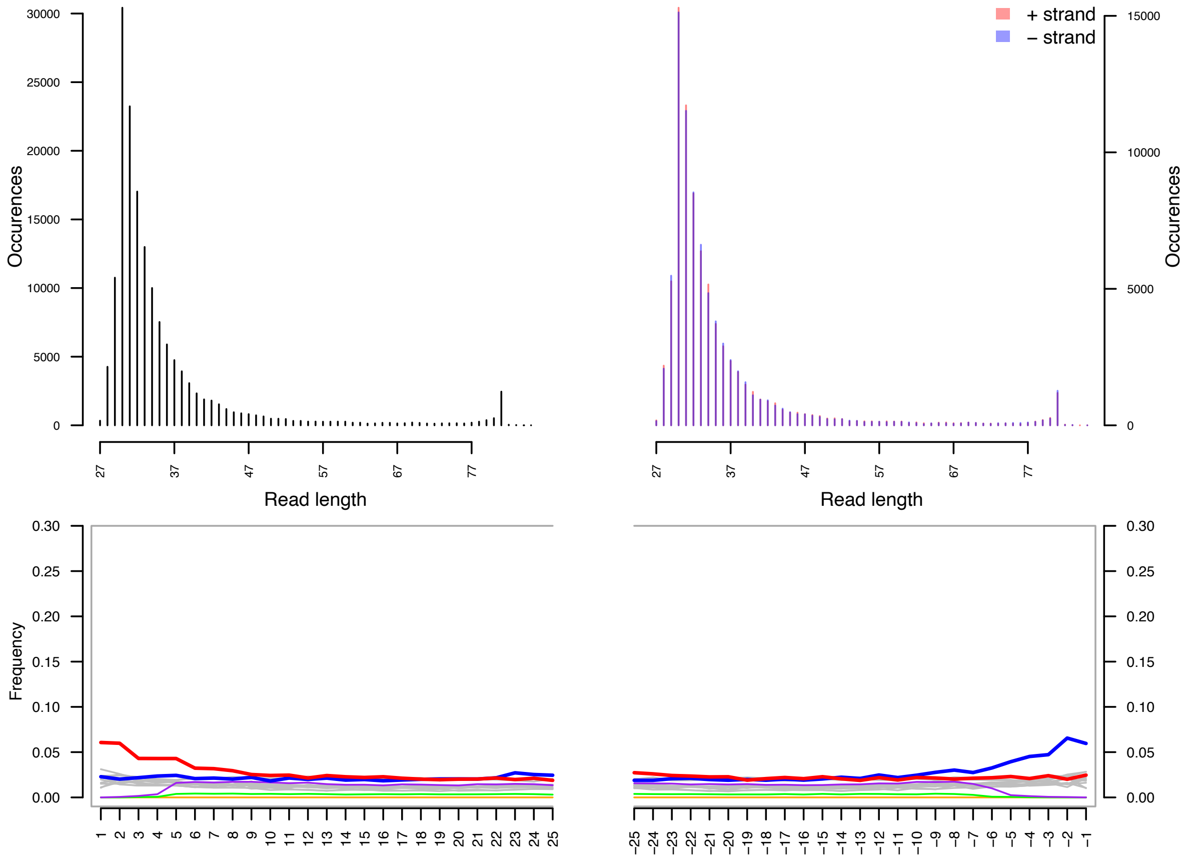

Figure S14. Damage and fragmentation estimates for reads aligned to the whole human genome (Hs37d5) for the SJArg-1-Nit. Single-end length distribution (top left panel) and per strand (top right panel). The bottom two plots are the positions' specific substitutions from the 5" (left) and the 3" end (right). The following color codes are used in the bottom plots: **Red**: C to T substitutions. **Blue**: G to A substitutions. **Grey**: All other substitutions. **Orange**: Soft-clipped bases. **Green**: Deletions relative to the reference. **Purple**: Insertions relative to the reference.

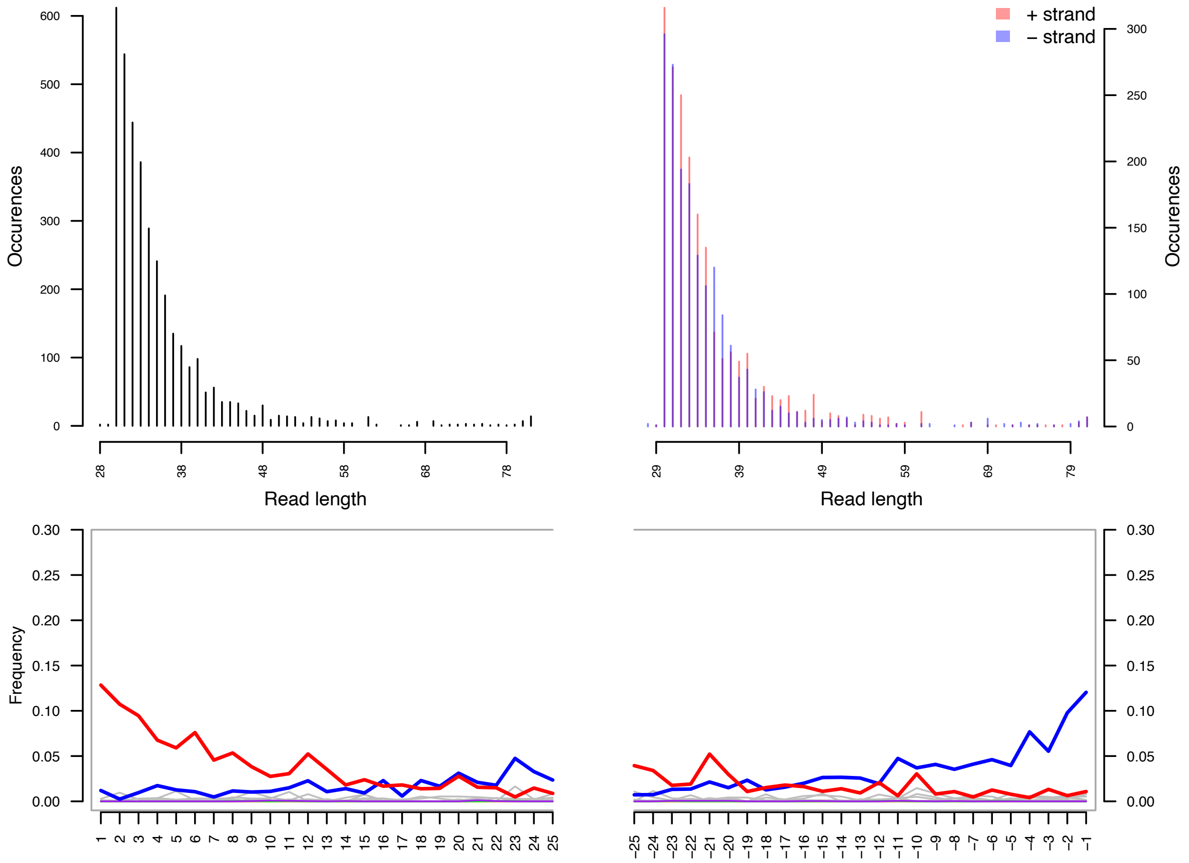

Figure S15. Damage and fragmentation estimates for reads aligned to the mitochondrial genome (rCRS) for the SJArg-1-Nit. Single-end length distribution (top left panel) and per strand (top right panel). The bottom two plots are the positions' specific substitutions from the 5" (left) and the 3" end (right). The following color codes are used in the bottom plots: **Red**: C to T substitutions. **Blue**: G to A substitutions. **Grey**: All other substitutions. **Orange**: Soft-clipped bases. **Green**: Deletions relative to the reference. **Purple**: Insertions relative to the reference.

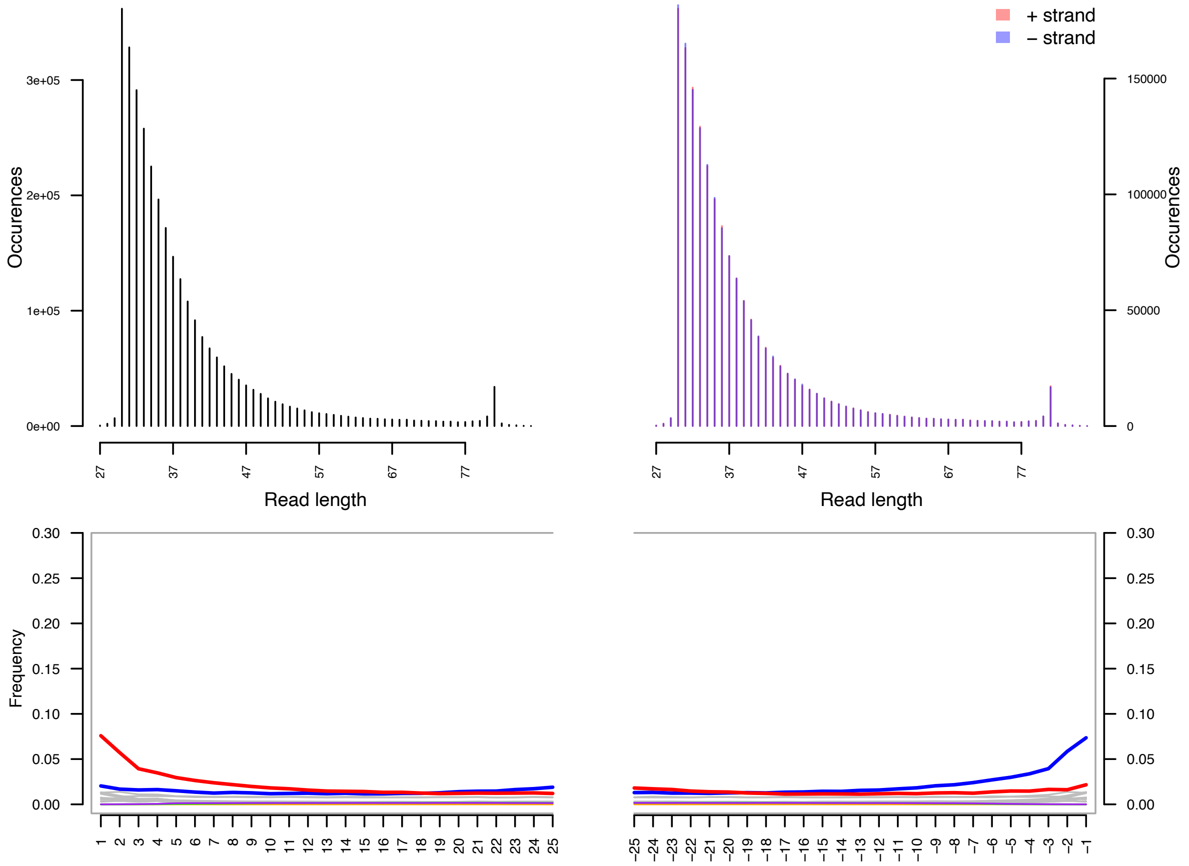

Figure S16. Damage and fragmentation estimates for reads aligned to the *Pediculus humanus corporis* scaffold (PRJNA16223) for the SJArg-1-Nit. Single-end length distribution (top left panel) and per strand (top right panel). The bottom two plots are the positions' specific substitutions from the 5" (left) and the 3" end (right). The following color codes are used in the bottom plots: **Red**: C to T substitutions. **Blue**: G to A substitutions. **Grey**: All other substitutions. **Orange**: Soft-clipped bases. **Green**: Deletions relative to the reference. **Purple**: Insertions relative to the reference.

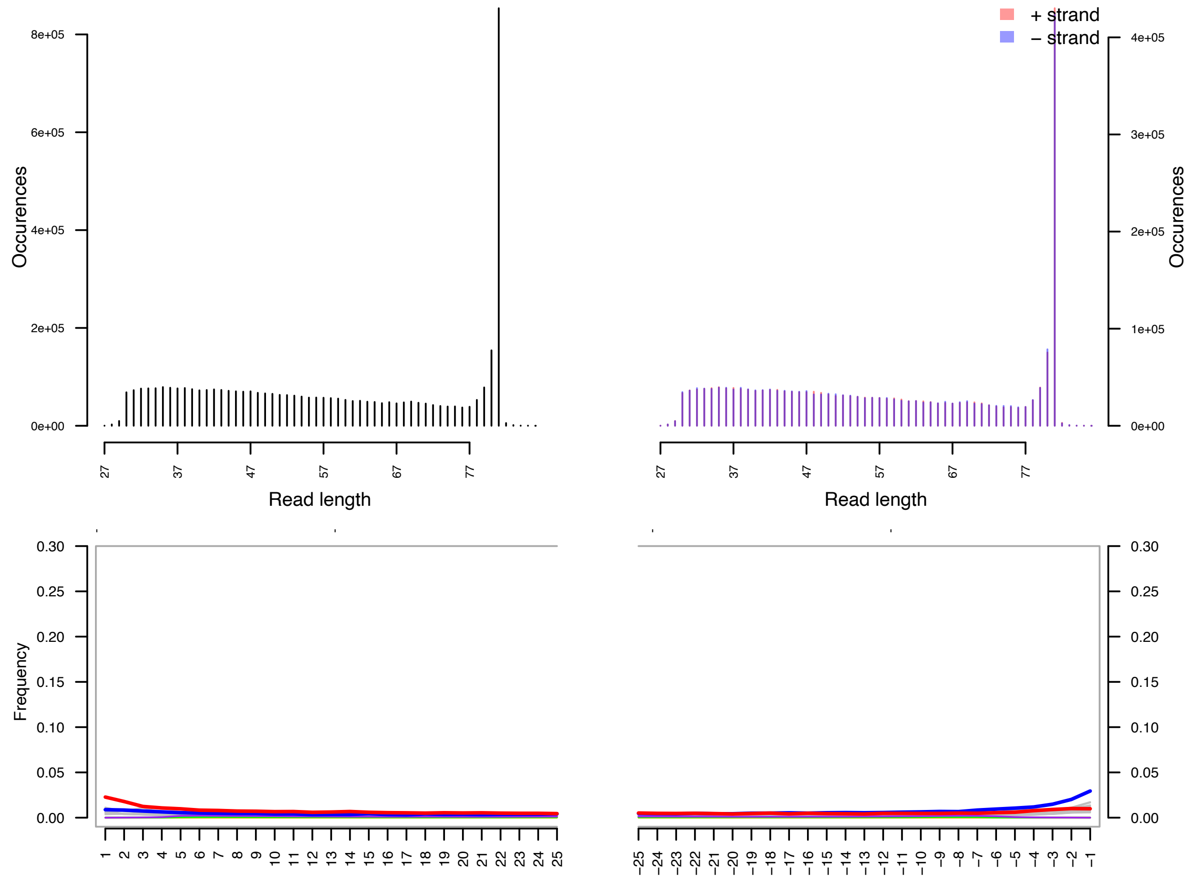

Figure S17. Damage and fragmentation estimates for reads aligned to the whole human genome (Hs37d5) for the SJArg-2-Nit. Single-end length distribution (top left panel) and per strand (top right panel). The bottom two plots are the positions' specific substitutions from the 5" (left) and the 3" end (right). The following color codes are used in the bottom plots: **Red**: C to T substitutions. **Blue**: G to A substitutions. **Grey**: All other substitutions. **Orange**: Soft-clipped bases. **Green**: Deletions relative to the reference. **Purple**: Insertions relative to the reference.

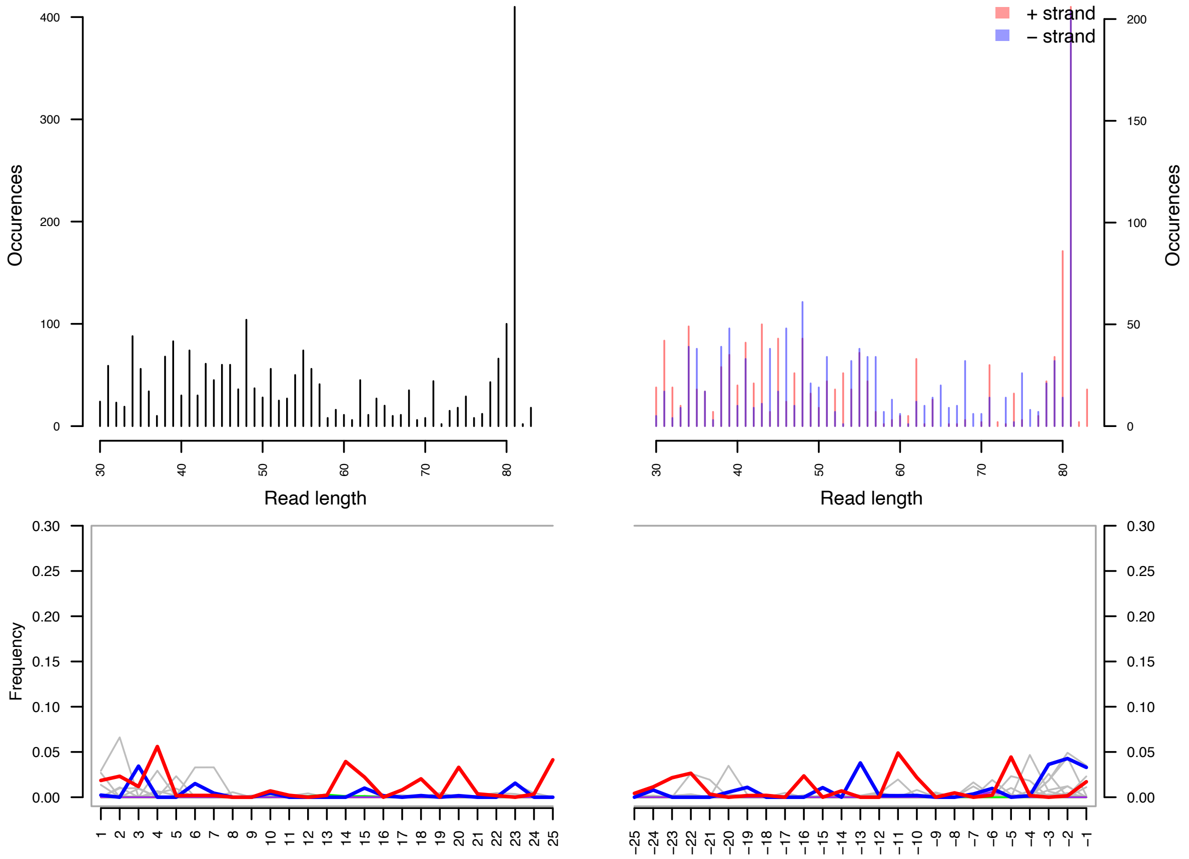

Figure S18. Damage and fragmentation estimates for reads aligned to the mitochondrial genome (rCRS) for the SJArg-2-Nit. Single-end length distribution (top left panel) and per strand (top right panel). The bottom two plots are the positions' specific substitutions from the 5" (left) and the 3" end (right). The following color codes are used in the bottom plots: **Red**: C to T substitutions. **Blue**: G to A substitutions. **Grey**: All other substitutions. **Orange**: Soft-clipped bases. **Green**: Deletions relative to the reference. **Purple**: Insertions relative to the reference.

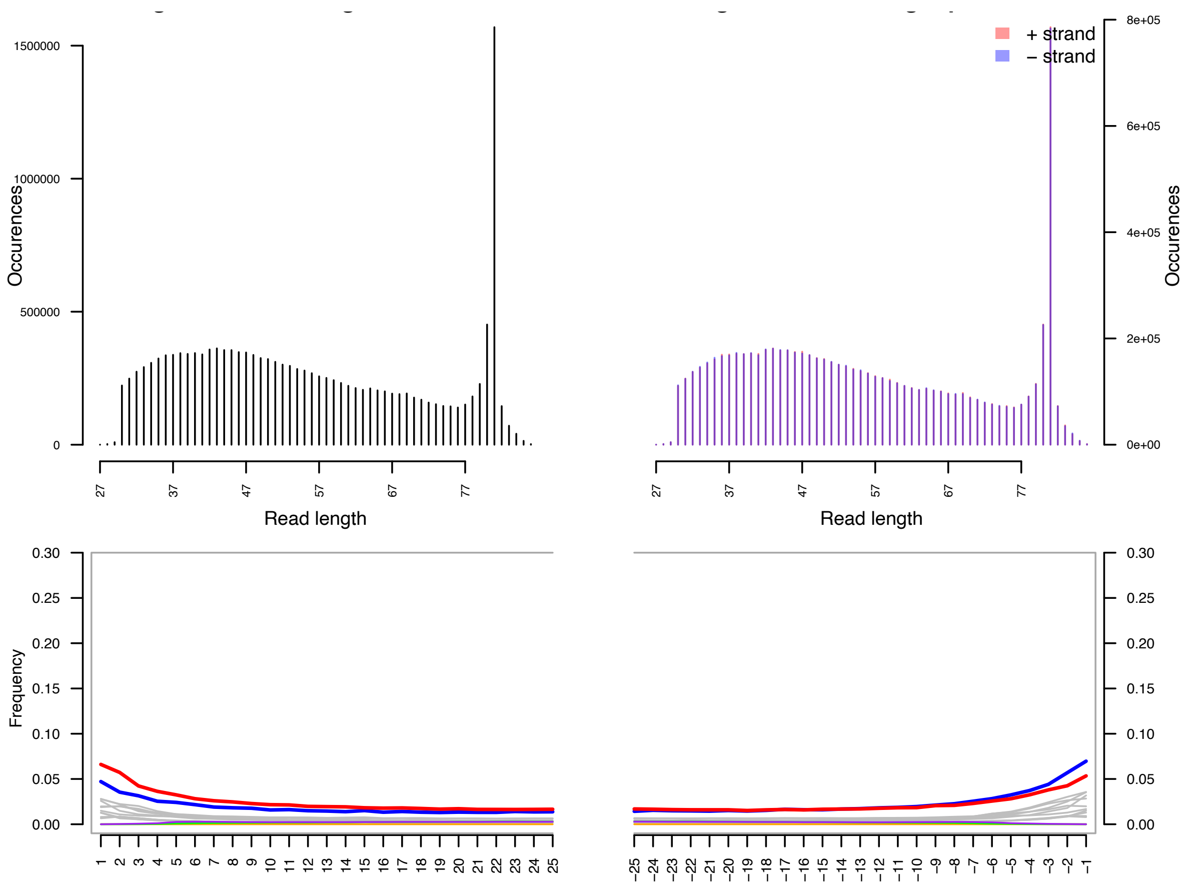

Figure S19. Damage and fragmentation estimates for reads aligned to the *Pediculus humanus corporis* scaffold (PRJNA16223) for the SJArg-2-Nit. The plots are very noisy due to too low number of reads. Single-end length distribution (top left panel) and per strand (top right panel). The bottom two plots are the positions' specific substitutions from the 5" (left) and the 3" end (right). The following color codes are used in the bottom plots: **Red**: C to T substitutions. **Blue**: G to A substitutions. **Grey**: All other substitutions. **Orange**: Soft-clipped bases. **Green**: Deletions relative to the reference. **Purple**: Insertions relative to the reference.

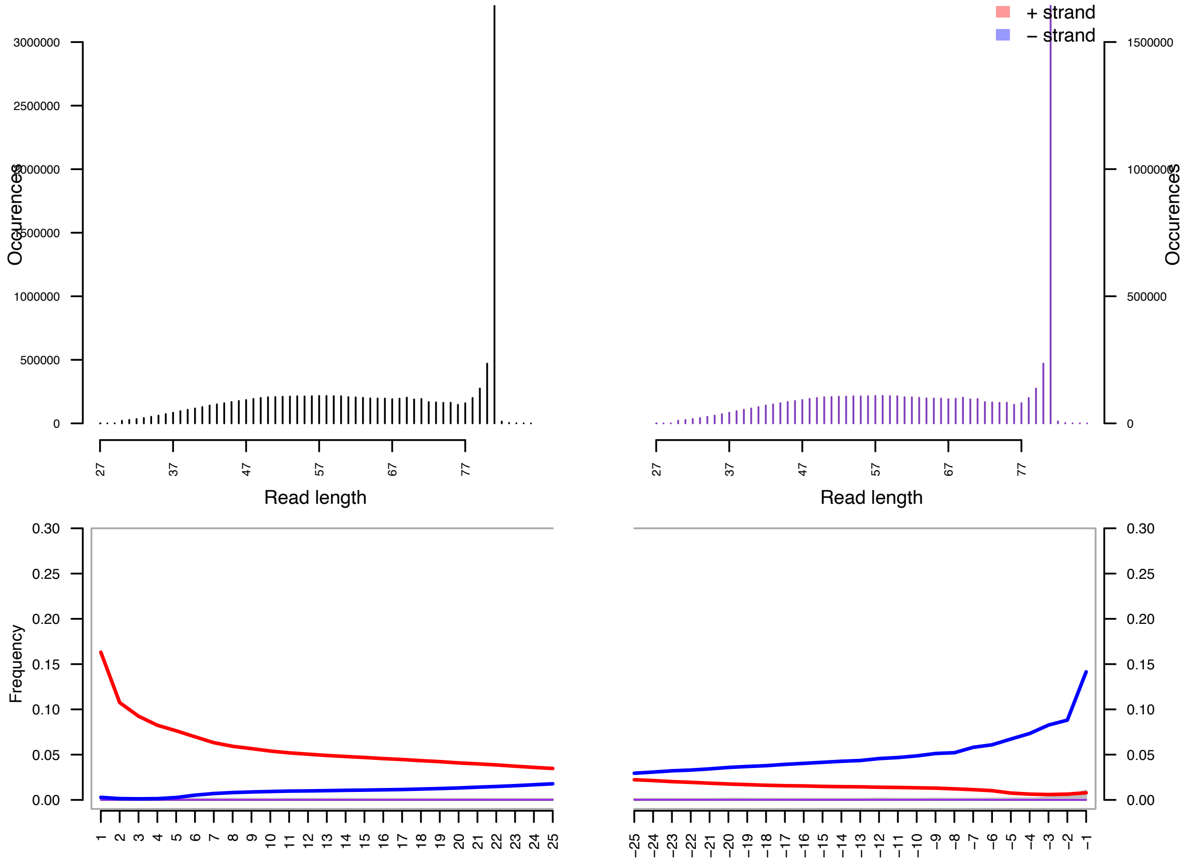

Figure S20. Damage and fragmentation estimates for reads aligned to the whole human genome (Hs37d5) for the SJArg-1-Tooth. Single-end length distribution (top left panel) and per strand (top right panel). The bottom two plots are the positions' specific substitutions from the 5" (left) and the 3" end (right). The following color codes are used in the bottom plots: **Red**: C to T substitutions. **Blue**: G to A substitutions. **Grey**: All other substitutions. **Orange**: Soft-clipped bases. **Green**: Deletions relative to the reference. **Purple**: Insertions relative to the reference.

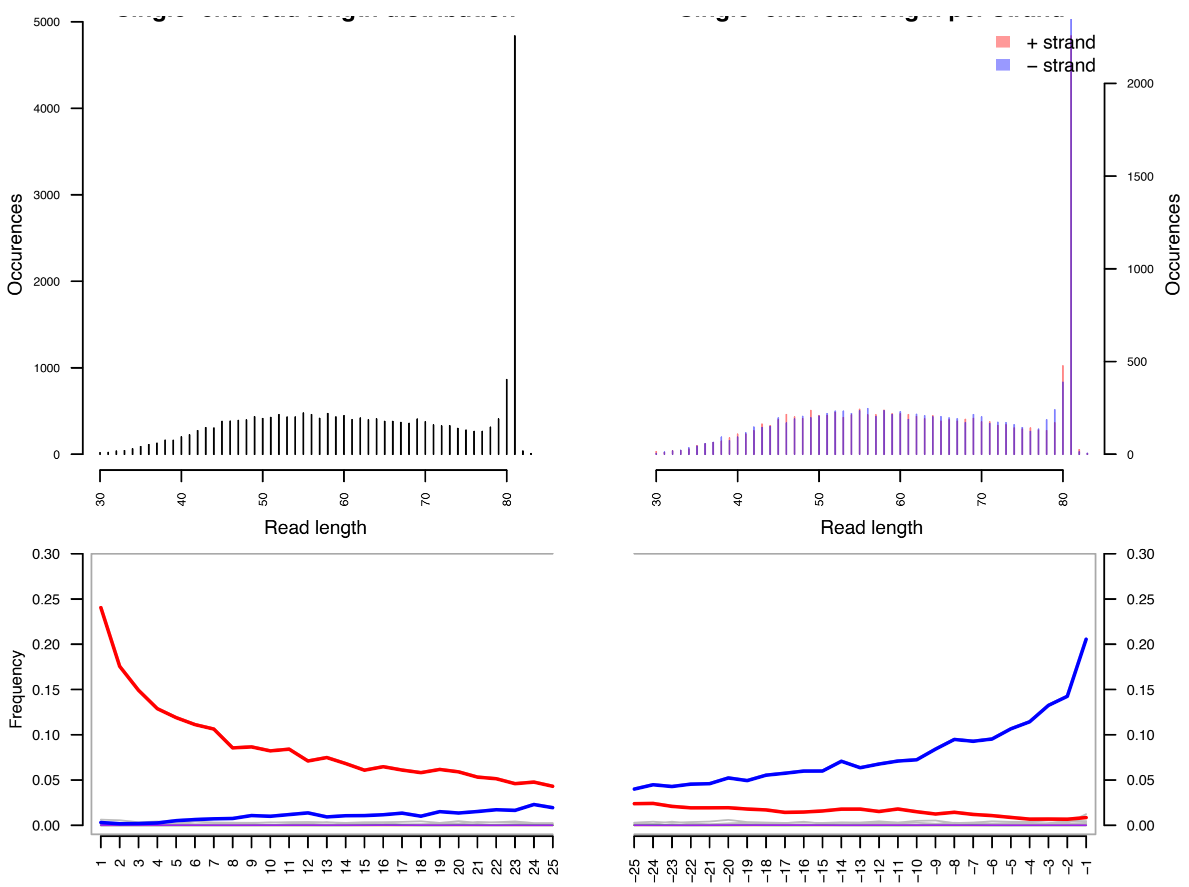

Figure S21. Damage and fragmentation estimates for reads aligned to the mitochondrial genome (rCRS) for the SJArg-1-Tooth. Single-end length distribution (top left panel) and per strand (top right panel). The bottom two plots are the positions' specific substitutions from the 5" (left) and the 3" end (right). The following color codes are used in the bottom plots: **Red**: C to T substitutions. **Blue**: G to A substitutions. **Grey**: All other substitutions. **Orange**: Soft-clipped bases. **Green**: Deletions relative to the reference. **Purple**: Insertions relative to the reference.

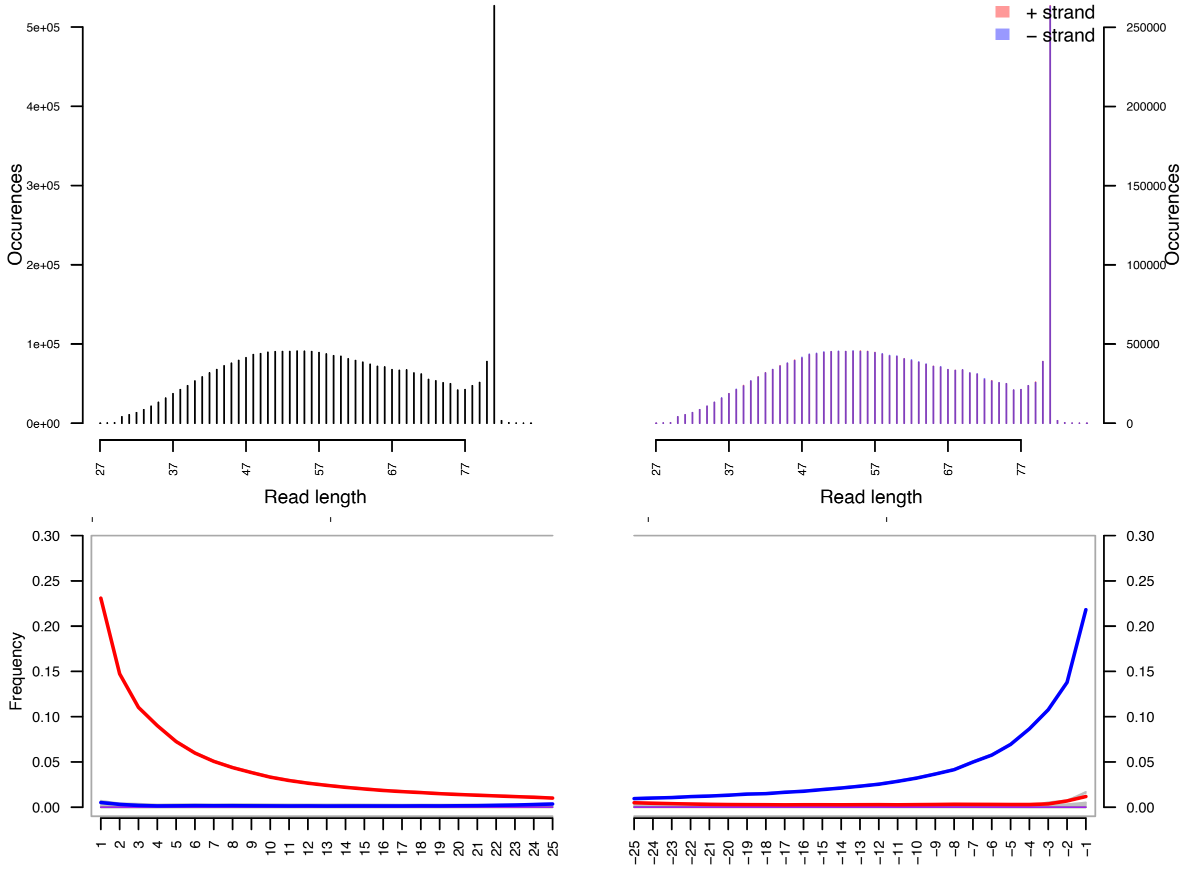

Figure S22. Damage and fragmentation estimates for reads aligned to the whole human genome (Hs37d5) for the SJArg-3-Petrous. Single-end length distribution (top left panel) and per strand (top right panel). The bottom two plots are the positions' specific substitutions from the 5" (left) and the 3" end (right). The following color codes are used in the bottom plots: **Red**: C to T substitutions. **Blue**: G to A substitutions. **Grey**: All other substitutions. **Orange**: Soft-clipped bases. **Green**: Deletions relative to the reference. **Purple**: Insertions relative to the reference.

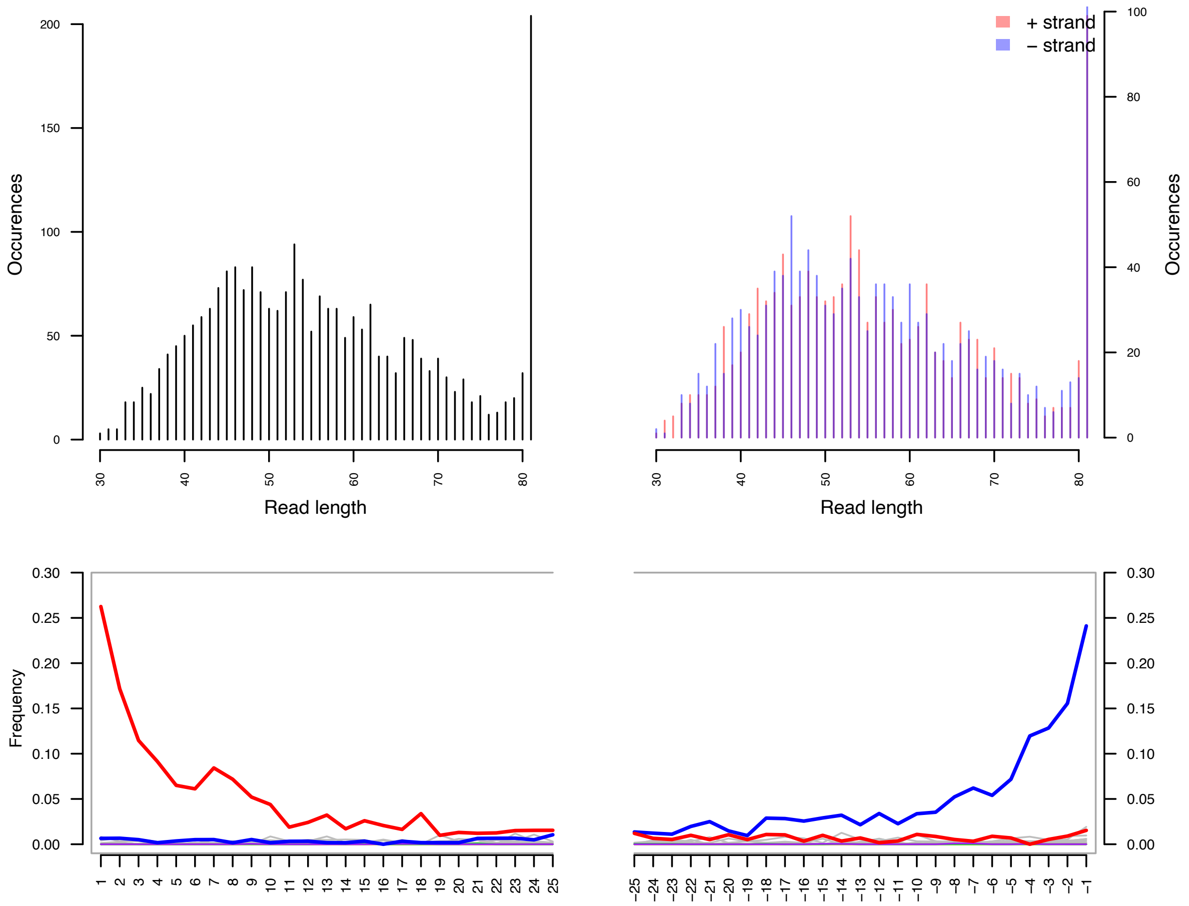

Figure S23. Damage and fragmentation estimates for reads aligned to the mitochondrial genome (rCRS) for the SJArg-3-Petrous. Single-end length distribution (top left panel) and per strand (top right panel). The bottom two plots are the positions' specific substitutions from the 5" (left) and the 3" end (right). The following color codes are used in the bottom plots: **Red**: C to T substitutions. **Blue**: G to A substitutions. **Grey**: All other substitutions. **Orange**: Soft-clipped bases. **Green**: Deletions relative to the reference. **Purple**: Insertions relative to the reference.

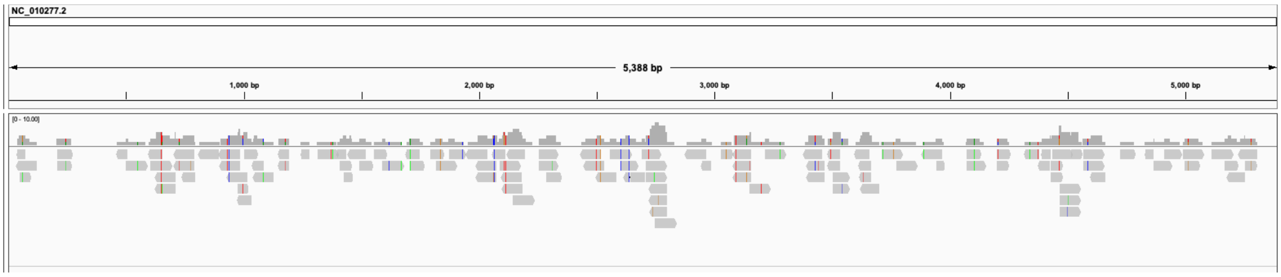

Figure S24. Distribution of the 115 reads uniquely assigned by Holi to Merkel cell polyomavirus plotted using IGV, here realigned against the complete reference genome (NC_010277.2, Merkel cell polyomavirus isolate R17b). Reads cover 74% of the genome corresponding to 3929 bases of the 5387 of the complete genome and has a mean depth of 1.4x.

Table S1. Radiocarbon dating (C^{14}) of specimens used in this study from Argentinian and Chilean institutions. Years BP as provided/estimated by the institutions and museums of origin.

**Code This work**	**Code Catalogue Museums**	**Code dating f/museums**	**Radiocarbon Age (years BP)**	**Median calibrated Radiocarbon ages (cal. B.P.) with 95.4% probability**	**Institution dating and sample type: sediments or remains**	**Source**
SJArg-1-Nit	2131	SAM0014 human hair	1568 ± 24	1461 (1389-1520)	SUERC-81423: Hair sample	This work
SJArg-2-Nit	589	GaK 5522	1990 ± 140	1934 (1689-2314)	Gakushuin University Japan Sediments	Instituto de Investigaciones Arqueológicas y Museo “Prof. Mariano Gambier” UNSJ (Gambier 1977)
SJArg-4-Nit	588	GaK 4809	1280 ± 90	1192 (975-1345)	Gakushuin University Japan Sediments	
SJArg-1-Tooth	2131B	GaK 4522	1580 ± 60	1461 (1345-1586)	Gakushuin University Japan Sediments	
SJArg-3-Petrous	2138	Beta-51832	1110 ± 50	1018 (926-1175)	Beta Analytic Miami, US Sediments	
SJArg-5-Nit	1937	Beta-161362*	590 ± 40	601 (530-650)	Beta Analytic Miami, US Sediments nearby burial	
SJArg-6-Nit	1228	Beta-107203*	880 ± 50	785 (690-911)	Beta Analytic Miami, US Sediments	
SJArg-7-Nit	1603	Beta-161362*	590 ± 40	601 (530-650)	Beta Analytic Miami, US Sediments nearby burial	
Chi-8-Nit	2587	Unknown	~300		Not dated – Estimated by similarity to cultural enclave	Museo Chileno de Arte Precolombino
Chi-9-Nit	T-458 6	Unknown	~400		Not dated – Estimated by cultural association	

*Datings provided by the source, already calibrated

Table S2. List of ancient human specimens included in this study, originating in Argentina and Chile museums.

Code assigned for this work	Original Code Gambier Institute	Material used, studies, this work	Permits**
SJArg-1-Tooth	2131	tooth for aDNA	Yes<br>A-B-C
SJArg-3-Petrous	2138	Petrous bone for aDNA	Yes<br>A-B-C
SJ-Arg-1-Nit§	2131B	6 nits for aDNA; 5 nits for cytogenetics, length of cement; 10 nits for root distance*	Yes<br>A-B-C
SJArg-2-Nit§	589	6 nits for aDNA; 7 nits for cytogenetics, length of cement; 14 nits for root distance*	Yes<br>A-B-C
SJArg-4-Nit§	588	8 for cytogenetics, length of cement; 20 nits for root distance*	Yes<br>A-B-C
SJArg-5-Nit§	1937	3 nits for cytogenetics, length of cement	Yes<br>A-B-C
SJArg-6-Nit§	1228	2 nits for cytogenetics, length of cement	Yes<br>A-B-C
SJArg-7-Nit§	1603	3 nits for cytogenetics, length of cement	Yes<br>A-B-C
Chi-8-Nit§	2587	5 nits for cytogenetics, length of cement	Yes<br>D
Chi-9-Nit§	T-458 6	3 nits for cytogenetics, length of cement	Yes<br>D
Dyak/Dayak-Lice§	Dyak (Lme?)# Sarawak	2 lice	Yes<br>E

*Measurements of distance between nit to scalp/hair-root were carried out *in situ*, on the mummies head.
#The specimen label in the collection was handwritten and some parts of the label illegible
§All lice and nits were identified as *Pediculus* Linnaeus by M. Alejandra Perotti
**Ethical approvals/Permits/Certificates A to E are listed in SI Note 1, page 3.

Table S3. Length of nit cement covering hair (µm) by mummy and age of mummy (Years BP). Summary statistics at the bottom of the table. Cal Years BP included here used the original uncalibrated ages provided and estimated by the museums.

Mummy Code	Nit No	Cement Length (µm)	Cal/Years BP
SJArg-1-Nit	10.4 stack 001	399.00	1461.00
SJArg-1-Nit	10.1 stack 001	468.00	1461.00
SJArg-1-Nit	10.11 stack 001	520.00	1461.00
SJArg-1-Nit	10.12 stack 001	536.00	1461.00
SJArg-1-Nit	10.7 stack 001	621.00	1461.00
SJArg-2-Nit	5.6 stack 001	385.00	1934.00
SJArg-2-Nit	5.3 stack 003	389.00	1934.00
SJArg-2-Nit	5.8 stack 001	438.00	1934.00
SJArg-2-Nit	5.7 stack 001	439.00	1934.00
SJArg-2-Nit	5.2 stack 001	477.00	1934.00
SJArg-2-Nit	5.10 stack 001	477.00	1934.00
SJArg-4-Nit	4.15 stack 001	433.00	1192.00
SJArg-4-Nit	4.7 stack 001	443.00	1192.00
SJArg-4-Nit	4.5 stack 001	473.00	1192.00
SJArg-4-Nit	4.12 stack 001	481.00	1192.00
SJArg-4-Nit	4.8 stack 001	511.00	1192.00
SJArg-4-Nit	4.4 stack 001	525.00	1192.00
SJArg-4-Nit	4.2 Stack	530.00	1192.00
SJArg-4-Nit	4.13 stack 001	559.00	1192.00
SJArg-5-Nit	12.1 stack 001	449.00	601.00
SJArg-5-Nit	12.3 10x	726.00	601.00
SJArg-5-Nit	12.2 stack 001	824.00	601.00
SJArg-6-Nit	7.1 stack 001	581.00	785.00
SJArg-6-Nit	7.4 stack 001	769.00	785.00
SJArg-7-Nit	8.1 stack 001	552.00	601.00
SJArg-7-Nit	8.3 stack 001	560.00	601.00
SJArg-7-Nit	8.2 stack 001	761.00	601.00
Chi-8-Nit	35.3 stack 001	474.00	300.00*
Chi-8-Nit	35.4 stack 001	662.00	300.00*
Chi-8-Nit	35.8 stack 001	747.00	300.00*
Chi-8-Nit	35.9 stack 001	821.00	300.00*
Chi-8-Nit	35.6 stack 001	841.00	300.00*
Chi-9-NIT	45.5 stack 001	677.00	400.00*
Chi-9-NIT	45.3 stack 001	682.00	400.00*
Chi-9-NIT	45.2 stack 001	736.00	400.00*

*Not directly dated, years BP estimated by cultural association

Table S4. Summary statistics of length of cement by age

Age (Cal/years BP)	300†	400•	601*	785	1192†	1461†	1934*†•
N	5	3	6	2	8	5	6
Min	474	677	449	581	433	399	385
Max	841	736	824	769	559	621	477
Mean	709	698	645	675	494	508	434
Std. Dev.	149	32	146	133	44	82	40

*†• Symbols denoting significantly different lengths between ages. Tukey's pairwise mean comparisons, $P<0.01$.

Table S5. Distance of nits from root (mm) by mummy (and age of mummy). Summary statistics at the bottom of the table. Years BP listed as provided/estimated by the museums.

ID Mummy - Code	Nit specimen	Distance f/root (mm)	Cal/years BP
SJArg-1-Nit	1	3	1461.00
SJArg-1-Nit	2	3	1461.00
SJArg-1-Nit	3	7	1461.00
SJArg-1-Nit	4	6	1461.00
SJArg-1-Nit	5	3	1461.00
SJArg-1-Nit	6	4	1461.00
SJArg-1-Nit	7	3	1461.00
SJArg-1-Nit	8	8	1461.00
SJArg-1-Nit	9	5	1461.00
SJArg-1-Nit	10	5	1461.00
SJArg-2-Nit	1	5	1934.00
SJArg-2-Nit	2	10	1934.00
SJArg-2-Nit	3	8	1934.00
SJArg-2-Nit	4	9	1934.00
SJArg-2-Nit	5	7	1934.00
SJArg-2-Nit	6	9	1934.00
SJArg-2-Nit	7	7	1934.00
SJArg-2-Nit	8	10	1934.00
SJArg-2-Nit	9	3	1934.00
SJArg-2-Nit	10	4	1934.00
SJArg-2-Nit	11	6	1934.00
SJArg-2-Nit	12	6	1934.00
SJArg-2-Nit	13	7	1934.00
SJArg-2-Nit	14	5	1934.00
SJArg-4-Nit	1	3	1192.00
SJArg-4-Nit	2	4	1192.00
SJArg-4-Nit	3	2	1192.00
SJArg-4-Nit	4	12	1192.00
SJArg-4-Nit	5	3	1192.00
SJArg-4-Nit	6	3	1192.00
SJArg-4-Nit	7	3	1192.00
SJArg-4-Nit	8	3	1192.00
SJArg-4-Nit	9	2	1192.00
SJArg-4-Nit	10	2	1192.00
SJArg-4-Nit	11	5	1192.00
SJArg-4-Nit	12	4	1192.00
SJArg-4-Nit	13	4	1192.00
SJArg-4-Nit	14	9	1192.00
SJArg-4-Nit	15	5	1192.00
SJArg-4-Nit	16	4	1192.00
SJArg-4-Nit	17	6	1192.00
SJArg-4-Nit	18	6	1192.00
SJArg-4-Nit	19	2	1192.00
SJArg-4-Nit	20	3	1192.00

Table S6. Summary statistics of distance from root (mm) by mummy

	SJArg-4-Nit†	SJArg-2-Nit*	SJArg-1-Nit*
**N**	20	10	14
**Min**	2	3	3
**Max**	12	8	10
**Mean**	4.2	4.7	6.8
**Std. Dev.**	6.3	3.3	4.7
**Median**	3.5	4.5	7

Symbols denoting Normally distributed* and not-Normally distributed† distance from root (Shapiro-Wilkinson test)

Table S7. Genetic metadata

SampleID	Dyak-Louse	SJArg-1-Nit	SJArg-1-Tooth	SJArg-2-Nit	SJArg-3-Petrous	Extraction blank
Total number of trimmed reads (q>29 and bp > 29)	40458463	10146734	108235505	37343964	88189394	485321
Number of reads to human (Hs37d5) with aln -n 0.01 -o 2 -l 1024 MQ >24 after rmdup	1140195	157074	11945856	4041901	3749070	2773
Number of reads to human mtDNA (rCRS) with aln -n 0.01 -o 2 -l 1024 MQ >24 after rmdup	91	3594	21415	2339	2486	2
Percentage human reads	2.818	1.548	11.037	10.823	4.251	0.571
Weighted mean wgs depth BWA	0.977	0.249	22.605	1.724	7.586	0.004
Breadth of coverage wgs	0.581	0.109	19.954	1.095	6.386	0.003
Weighted mean mtDNA depth BWA	0.1	1.7	29.9	0.8	3.9	0.01
Breadth of coverage mtDNA	13.43	96.32	99.96	75.63	99.32	0.43
DNA damage (human wgs) C->T 5"	0.062	0.061	0.163	0.023	0.231	0.010
DNA damage (human mtDNA) C->T 5"	Too few reads	0.13	0.24	0.02	0.26	Too few reads
Haplogroup	NA	A2+(64)	A2+(64)	A2+(64)+@153	A2+(64)	NA
Total number of reads mapping to Pediculus humanus corporis (PRJNA16223) Bowtie2 end-to-end q>24	26518926	2280723	NA	9883857	NA	NA
Percent reads mapping to Pediculus	65.5	22.5	NA	26.5	NA	NA
Breadth of coverage Pediculus	84.583	40.132	NA	60.780	NA	NA
DNA damage (Pediculus humanus corporis (PRJNA16223)) C->T 5"	0.106	0.076	NA	0.066	NA	NA
Specimen age [y BP]	~170	~1580	~1460	~1950	~1020	NA
Host	Individual from the Dayak culture	South America human mummy	South America human mummy	South America human mummy	South America human mummy	NA
Site location	Sarawak, Malaysia, SE Asia	Ansilta; Calingasta, San Juan, South America	Ansilta; Calingasta, San Juan, South America	Ansilta; Calingasta, San Juan, South America	Cerro Calvario IV, San Juan, South America	NA

Table S8. Y chromosomal haplogroup placement of SJArg-2-Nit.

Haplogroup	Site	SNP_name	SNP_synonym	Reference allele	Alternative allele
BT	21259569	M9311		C	T
K2b2a	17559652	CTS7604	M1235; PF5928;	T	C
Q1b	17625916	CTS7714	M1137	T	G
Q1b1a1a1	21546493	M907	Y775	G	A

REFERENCES

Busvine JR. 1948. The head and body races of *Pediculus humanus* L. Parasitology 39(1-2):1-16.

Buxton PA. 1940. Temperatures lethal to the louse. Br Med J 1:341.

Gambier M. 1977. La Cultura de Ansilta. In: Gambier M, editor. La Cultura de Ansilta. San Juan, Argentina: Inst. Investigaciones Arqueológicas y Museo, Universidad Nacional de San Juan.

Hammer Ø, Harper DAT, Ryan PD. 2001. Paleontological statistics software package for education and data analysis. Palaeontol Elect 4:9pp.

Leeson HS. 1941a. The effect of temperature upon the hatching of the eggs of *Pediculus humanus corporis* de Geer (Anoplura). Parasitology 33(243-249).

Leeson HS. 1941b. The survival of unfed *Pediculus humanus corporis*, De Geer (Anoplura) at different temperatures. Parasitology 32(1):49-51.

Maunder JW. 1983. The appreciation of lice. Proc R Inst G Br 55:1-32.
